# Migration sources and pathways of the pest species *Sogatella furcifera* in Yunnan, China, and across the border inferred from DNA and wind analyses

**DOI:** 10.1002/ece3.6531

**Published:** 2020-07-17

**Authors:** Shao‐Ji Hu, Shan‐Shan Sun, Da‐Ying Fu, Jian‐Ping Lü, Xue‐Ying Wang, Yan‐Ping Yu, Li‐Min Dong, Sui‐Yun Chen, Hui Ye

**Affiliations:** ^1^ Yunnan Key Laboratory of International Rivers and Transboundary Eco‐security Yunnan University Kunming China; ^2^ Institute of International Rivers and Eco‐security Yunnan University Kunming China; ^3^ Department of Atmospheric Sciences School of Resource Environment and Earth Science Yunnan University Kunming China; ^4^ School of Life Sciences Southwest Forest University Kunming China; ^5^ Plant Protection and Quarantine Station of Yunnan Province Kunming China; ^6^ School of Life Sciences Yunnan University Kunming China; ^7^ Biocontrol Engineering Research Centre of Crop Disease and Pest Yunnan University Kunming China; ^8^ Biocontrol Engineering Research Centre of Plant Disease and Pest Yunnan University Kunming China; ^9^ School of Agriculture Yunnan University Kunming China

**Keywords:** genetic connectivity, migration, pest management, planthopper, rice pest

## Abstract

The migration sources and pathways of *Sogatella furcifera* (Horváth) in topologically complex regions like Yunnan, China, and adjacent montane areas have long been a challenging task and a bottleneck in effective pest forecast and control. The present research reinvestigated this issue using a combination of mtDNA and long‐term historical wind field data in an attempt to provide new insights. Genetic analyses showed that the 60 populations of *S. furcufera* collected across Myanmar, Thailand, Laos, Vietnam, Yunnan, Guizhou, and Sichuan lack genetic structure and geographic isolation, while spatial analysis of haplotype and diversity indices discovered geographic relevance between populations. Migration rate analysis combined with high‐resolution 10‐year wind field analysis detected the following migration sources, pathways, and impacted areas which could explain the outbreak pattern in Yunnan. (a) Dominating stepwise northward migrations originated from northern Indochina, southern Yunnan, and central‐eastern Yunnan, impacting their northern areas. (b) Concurring summer–autumn southward (return) migration originated from nearly all latitude belts of Sichuan and Yunnan mainly impacting central and southern Yunnan. (c) Regular eastward and summer–autumn westward migrations across Yunnan. The northward migration reflects the temporal rhythm of gradual outbreaks from the south to the north in a year, while the return migration may explain the repeated or very severe outbreaks in the impacted areas. To form a better pest forecast and control network, attention must also be paid to the northern part of Yunnan to suppress the impact of return migration in summers and autumns.

## Graphical abstract

Molecular and meteorological methods were applied to the migration analysis of *Sogatella furcifera* in Yunnan and across border, identified migration source and pathways. It is also confirmed that return migration could occur in summer and autumn with northerly bursts across the region.

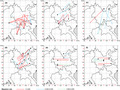

## 1 INTRODUCTION

The white‐backed planthopper, *Sogatella furcifera* (Horváth) (Hemiptera: Delphacidae), is a devastating migratory rice pest in Asia, especially in South China and northern Indochina (Catindig et al., 2009; Cheng, 2015). Planthoppers can migrate as far as 500 km facilitated by air currents (Hu et al., 2014, 2018; Ma et al., 2018). Since 2007, rice production in Yunnan and its bordering provinces in Southwest China has been severely impacted by this pest due to its migratory behavior and serious resulting damage (Gui, Li, Han, Li, & Lü, 2008; Hu, Lu, et al., 2016; Hu, Cheng, et al., 2016; Zhao et al., 2014).

Migration sources and pathways have long been two bottleneck issues in monitoring, forecasting, and controlling rice planthoppers (Hu, 2013; Zhai, 2011). In the past three decades, many methods including air trajectory simulation and insect radar were applied to partly solve these problems (Hu, Lu, et al., 2016; Huang, Cheng, Chen, Wu, & Otuka, 2010; Otuka et al., 2010, 2012; Riley et al., 1991; Riley, Reynolds, & Farrow, 1987). In a recent decade, molecular biology using different markers (including mtDNA *cox1*, *cox2*; intersimple sequence repeats [ISSR], expressive sequence tags [EST], and microsatellite DNA) has also been applied to investigate the population genetics of rice planthoppers at different scales (Jing et al., 2011; Li et al., 2016; Liu, Gui, & Li, 2010; Matsumoto et al., 2013; Nam, Kim, & Lee, 2018; Qiu et al., 2016; Sun, Jiang, Wang, & Hong, 2014; Xie, Guo, Jin, & Wang, 2014). However, due to the high genetic connectivity between *S. furcifera* populations, identifying migration source and migration routes are challenging. Liu et al. (2010) analyzed 47 populations of *S. furcifera* in Yunnan and its adjacent areas, but did not clearly identify the sources and pathways of the insect. To date, only Nam et al. (2018) proposed putative migration sources and routes at a rather large scale across Indochina, South China, to Korea and Japan.

Yunnan province and its surroundings are the southeast marginal regions of the Tibetan Plateau, with complex topological and meteorological conditions (Wang & Zhang, 2002). On the one hand, Yunnan province is under the influence of both the Indian and East Asian Monsoons. One the other hand, the Yunnan–Guizhou Plateau could block westerly and easterly air flow and induce anticyclonic and cyclonic circulations. Therefore, in summer, in addition to the dominating northward and eastward wind fields, periodical bursts of southwestward winds also occur (Shi, Sha, & Liu, 2017). Air flows in almost all directions as well as the unique north–south mountain chain make the migration behavior mode of *S. furcifera* not entirely the same as that in East and South China (Hu, Dong, Wang, Chen, & Ye, 2019; Wu et al., 2017; Zhao et al., 2014). Furthermore, the low‐latitude and low‐altitude area of southern Yunnan bordering with Myanmar, Laos, and Vietnam is one of the very limited number of overwintering areas for *S. furcifera* in China (Hu, Fu, Han, & Ye, 2015; Hu, Liu, et al., 2015). Because of the diverse natural environment in this area, it has been challenging to reveal the migration source and pathways of *S. furcifera* there, although some previous endeavors partly addressed this issue (Li et al., 2016; Shen, 2010; Shen et al., 2011).

The present research aimed at the migration sources and pathways of *S. furcifera* in Yunnan and its adjacent areas, including southern Sichuan and a wide range of northern Indochina. A 60‐population (1,200‐individual) dataset was used to infer the genetic relationship and putative migration pathways among the 60 populations, and wind fields at different temporal scales were also reanalyzed to detect the most likely carrying winds in accordance with the migration pathways inferred from the molecular analyses. The findings of the present research would further elucidate the migration sources and pathways of *S. furcifera* in a topologically and meteorologically complex region, and could be used as a complementary to form a better understanding of the transboundary migration of *S. furcifera* in Southwest China and benefit the formulation of management strategies.

## 2 MATERIALS AND METHODS

### 2.1 Sample collection and preparation

**FIGURE 1:**
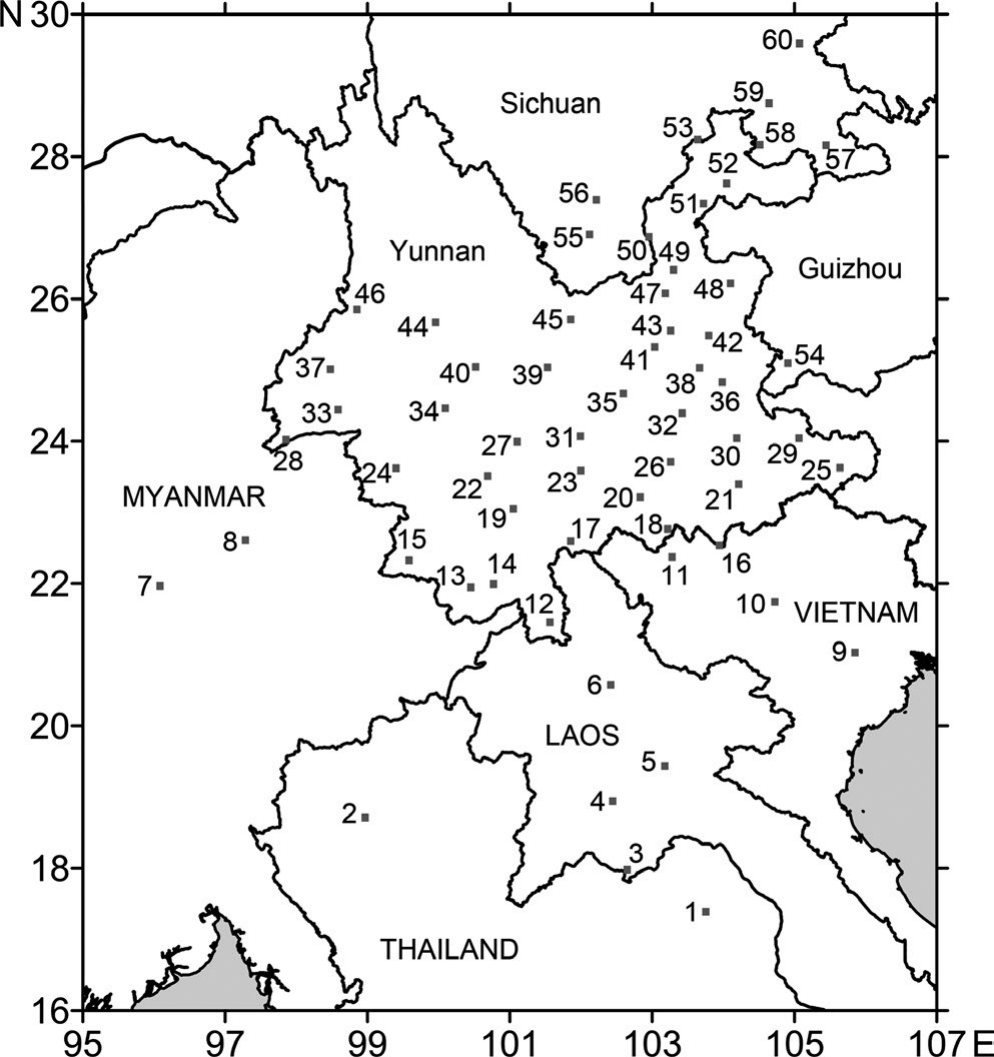
Map of the 60 sampling sites, with numbers corresponding to the sites introduced in Table 1. Map is generated by Surfer 10.0

In total, 60 sampling sites were selected to represent the research range, with 49 sites situated within China and 11 sites distributed in the northern portion of four adjacent Indochinese countries, namely Myanmar, Thailand, Laos, and Vietnam (Table 1, Figure 1), before the Nagoya Protocol of Access and Benefit Sharing (ABS) entered into force in 2014 (UN Convention on Biological Diversity, 2014). Sampling sites in northern Indochina were selected to cover the possible source areas (Sun, Bao, Wu, Lu, & Tuan, 2018; Wu et al., 2018).

**TABLE 1:** Summary information of 60 sampling sites and sample size, arranged in alphabetic order of countries/province names and latitude, groups correspond to those in Figure 2

Code	Population locality	Coordinates	Colleting month	Alt./m	Group
1	Thailand: Sakon Nakhon	17.3930, 103.7550	2012‐Feb.	169	TL
2	Thailand: Chiang Mai	18.7131, 98.9697	2012‐Feb.	300	TL
3	Laos: Vientiane	17.9811, 102.6478	2013‐Jun.	166	LA
4	Laos: Vang Vieng	18.9480, 102.4476	2013‐Jun.	245	LA
5	Laos: Phonsavan	19.4438, 103.1816	2013‐Jun.	1,101	LA
6	Laos: Pak Mong	20.5784, 102.4168	2013‐Jun.	376	LA
7	Myanmar: Mandalay	21.9736, 96.0851	2011‐May	67	MY
8	Myanmar: Hsipaw	22.6127, 97.2881	2011‐May	552	MY
9	Vietnam: Ha Noi	21.0332, 105.8492	2011‐Apr.	9	VN
10	Vietnam: Yen Bai	21.7490, 104.7220	2011‐Apr.	75	VN
11	Vietnam: Lai Chau	22.3780, 103.2820	2011‐Apr.	645	VN
12	Yunnan: Mengla	21.4607, 101.5631	2012‐Feb.	695	C34
13	Yunnan: Menghai	21.9490, 100.4540	2012‐Feb.	1,238	C34
14	Yunnan: Jinghong	21.9997, 100.7721	2012‐Feb.	910	C34
15	Yunnan: Menglian	22.3310, 99.5842	2012‐Feb.	1,106	D3
16	Yunnan: Hekou	22.5380, 103.9420	2012‐Feb.	80	D56
17	Yunnan: Jiangcheng	22.5990, 101.8540	2012‐Feb.	1,100	D4
18	Yunnan: Jinping	22.7699, 103.2220	2012‐Feb.	410	D56
19	Yunnan: Ning'er	23.0570, 101.0520	2012‐Feb.	1,301	D3
20	Yunnan: Yuanyang	23.2200, 102.8330	2012‐Feb.	235	D4
21	Yunnan: Wenshan	23.4006, 104.2167	2012‐Apr.	1,260	D56
22	Yunnan: Jinggu	23.5136, 100.6924	2012‐Mar.	933	D3
23	Yunnan: Yuanjiang	23.5892, 102.0010	2012‐Feb.	420	D4
24	Yunnan: Gengma	23.6240, 99.4040	2012‐Feb.	513	D3
25	Yunnan: Funing	23.6294, 105.6390	2012‐Mar.	970	D56
26	Yunnan: Kaiyuan	23.7137, 103.2591	2012‐Mar.	1,091	D56
27	Yunnan: Zhenyuan	24.0001, 101.1040	2012‐Mar.	1,492	D4
28	Yunnan: Ruili	24.0230, 97.8580	2012‐Feb.	812	E2
29	Yunnan: Guangnan	24.0440, 105.0650	2012‐Mar.	1,545	D56
30	Yunnan: Qiubei	24.0440, 104.1916	2012‐Apr.	1,272	D56
31	Yunnan: Xinping	24.0703, 101.9900	2012‐Apr.	1,086	E4
32	Yunnan: Mile	24.3970, 103.4230	2012‐Apr.	1,204	E5
33	Yunnan: Mangshi	24.4434, 98.5900	2012‐Feb.	892	E2
34	Yunnan: Yunxian	24.4670, 100.0960	2012‐Feb.	1,087	E3
35	Yunnan: Jinning	24.6757, 102.5986	2012‐Jun.	1,907	E4
36	Yunnan: Shizong	24.8341, 103.9883	2012‐Jul.	1,900	E5
37	Yunnan: Tengchong	25.0180, 98.4810	2012‐May	1,564	E2
38	Yunnan: Luliang	25.0383, 103.6697	2012‐Jul.	1,858	E5
39	Yunnan: Chuxiong	25.0453, 101.5288	2012‐Jul.	1,782	E4
40	Yunnan: Nanjian	25.0508, 100.5170	2012‐Jul.	1,780	E3
41	Yunnan: Songming	25.3304, 103.0380	2012‐Jul.	1,908	E4
42	Yunnan: Qujing	25.4879, 103.7975	2012‐Jul.	1,875	E5
43	Yunnan: Xundian	25.5561, 103.2578	2012‐Jul.	1,867	E5
44	Yunnan: Yangbi	25.6712, 99.9560	2012‐Jun.	1,568	E3
45	Yunnan: Yuanmou	25.7143, 101.8594	2012‐May	1,072	E4
46	Yunnan: Lujiangba	25.8543, 98.8529	2012‐Apr.	733	E2
47	Yunnan: Dongchuan	26.0802, 103.1850	2012‐Apr.	1,156	F5
48	Yunnan: Xuanwei	26.2244, 104.0982	2012‐Jul.	2,006	F5
49	Yunnan: Huize	26.4120, 103.3020	2012‐Jul.	2,134	F5
50	Yunnan: Qiaojia	26.8720, 102.9534	2012‐May	809	F5
51	Yunnan: Zhaotong	27.3407, 103.7178	2012‐Jul.	1,910	F5
52	Yunnan: Yiliang	27.6288, 104.0474	2012‐Jul.	618	F5
53	Yunnan: Yongshan	28.2440, 103.6370	2012‐Jul.	835	G56
54	Guizhou: Xingyi	25.1055, 104.9060	2012‐Jul.	1,245	E5
55	Sichuan: Miyi	26.9105, 102.1198	2012‐Jun.	1,095	F4
56	Sichuan: Dechang	27.3947, 102.2126	2012‐Jun.	1,334	F4
57	Sichuan: Xuyong	28.1590, 105.4470	2012‐Jun.	354	G56
58	Sichuan: Junlian	28.1690, 104.5140	2012‐Jun.	492	G56
59	Sichuan: Yibin	28.7544, 104.6420	2012‐Jun.	309	G56
60	Sichuan: Neijiang	29.5926, 105.0732	2012‐Jun.	331	G56

For taxonomical certainty, all samples collected were adult *S. furcifera*, and nymphs were excluded on site. Only the first generation or swarm of *S. furcifera* appearing in a given site of the current year was collected in order to obtain a better genetic profile of this migratory pest and prevent random genetic mixture during subsequent migration. Adult *S. furcifera* were collected by net sweeping from paddies and preserved instantly with absolute ethanol.

All samples were carefully examined under a stereoscope to exclude potential arthropod parasitoids (e.g., Strepsiptera) (Ding, 2006). After quality control screening, samples from different paddies of the same site were randomly mixed and transferred into absolute ethanol and preserved at −40°C until DNA extraction. For each population, 20 individuals were randomly selected from the storage for molecular analysis.

### 2.2 DNA extraction, PCR amplification, and sequencing

Twenty *S. furcifera* adults from each of the 60 sampling locations were used in the present research for DNA extraction. Prior to the extraction, all samples were treated with STE (50 mmol/L NaCl, 100 mmol/L Tris‐HCl, and 2 mmol/L EDTA‐Na_2_, pH = 8.0; BioBasic Inc., Ontario, Canada) by incubating at 37°C for 1 hr to eliminate residual ethanol. Then, each individual *S. furcifera* was homogenized in a labeled 1.5 ml Eppendorf tube. The subsequent digestion and phenol‐chloroform method used in the present study followed Hu et al. (2013), and the extracted DNA was preserved at −40°C in the same laboratory. One microliter of the straight DNA was applied as templates in polymerase chain reactions (PCR).

For all samples, a ~800 bp fragment of the *cox1* gene and a ~1 kb fragment of the *16s* gene were amplified by PCR on a Biometra T‐Professional Standard thermocycler (Biometra GmbH, Göttingen, Germany). The thermal profile consisted of an initial denaturation at 94°C for 3 min; followed by 35 cycles of denaturation at 94°C for 30 s, annealing at 47°C (*cox1*) or 50°C (*16s*) for 1 min, and elongation at 72°C for 2 min; then a final elongation at 72°C for 10 min.

The PCR reaction was applied in a 25 *μ*L system using TaKaRa Ex *Taq* Kit (TaKaRa Biotechnology Co., Ltd., Dalian, China), which contained 2.5 μl of 10× PCR buffer, 2.5 μl of MgCl_2_ (25 mmol/L), 4.0 μl of dNTP mixture (2.5 mmol/L each), 0.25 μl of Ex *Taq* polymerase (5 U/μl), and 0.5 μl of each of forward and reverse primers (20 μmol/L; Sangon Biological Engineering Technology & Services Co. Ltd., Shanghai, China). The primers for the *cox1* fragment were C1‐J‐2183 (alias “Jerry”) (5’‐CAA CAT TTA TTT TGA TTT TTT GG‐3’) and TL2‐N‐3014 (alias “Pat”) (5’‐TCC AAT GCA CTA ATC TGC CAT ATT A‐3’) (Simon et al., 1994), and the primers for the *16s* fragment were L16F (5’‐CCG GTC TGA ACT CAG ATC AT‐3’) and L16R (5’‐ATT TAT TGT ACC TTT TGT ATC AG‐3’) (Song & Liang, 2009).

The PCR products were purified using TaKaRa Agarose Gel Purification Kit (version 2.0) (TaKaRa Biotechnology Co., Ltd., Dalian, China) and sequenced by Sangon Biological Engineering Technology & Services Co., Ltd. (Shanghai, China). Sequencing reactions were carried out in both forward and reverse directions on an ABI Prism 3730xl automatic sequencer (Applied Biosystems, Foster City, CA, USA).

### 2.3 Sequence analysis and genetic indices

Raw sequences were proofread and aligned using Clustal W (Thompson, Higgins, & Gibson, 1994) in BioEdit 7.0.9 (Hall, 1999). The product sequences of *cox1* gene were checked by conceptual translation using the invertebrate mitochondrial criterion in MEGA 6.0 (Tamura, Stecher, Peterson, Filipski, & Kumar, 2013) to exclude the possibility of obtaining *Numts* (nuclear copies of mtDNA fragments) (Bertheau, Schuler, Krumböck, Arthofer, & Stauffer, 2011; Song, Buhay, Whiting, & Crandall, 2008), and both of the *cox1* and the *16s* gene fragments were cross checked against the genomic reference database in NCBI using BLASTn. A number of polymorphic sites and nucleotide composition were analyzed in MEGA 6.0.

Genetic distances between populations were calculated with Kimura's two‐parameter (K2P) model (Kimura, 1980) in MEGA 6.0 with 1,000 iterations. Nei's average number of pairwise differences between populations (Nei & Li, 1979), Nei's average number of pairwise differences within populations, pairwise *F*st values, haplotype diversity (*H*) and nucleotide diversity (*π*) of each population, and the degree of gene flow among populations (*N*_m_) were calculated in Arlequin 3.11 (Excoffier, Laval, & Schneider, 2005). The significance of Nei's average number of pairwise differences between populations and the pairwise *F*_ST_ values were tested by 1,000 iterations for statistical significance, and the optimal gamma shape used in Arlequin 3.11 was estimated by jModelTest 0.1 (Guindon & Gascuel, 2003; Posada, 2008).

Haplotypes were defined by DnaSP 5.0 (Librado & Rozas, 2009) and designated in numeral order, and the ratios of shared and private haplotypes for each population were calculated, respectively. Spatial distribution of all haplotypes mapped in pie charts using ArcMap 10 (Esri Inc., CA, USA). For a better understanding of the haplotype distribution, only shared haplotypes were assigned with different colors while all private haplotypes were intentionally left in white. Two maps were generated from the same dataset with one showing all haplotypes and the other containing the shared haplotypes only. The circle size of each pie chart of the latter map was adjusted in accordance with the total sample size of shared haplotypes.

Molecular diversity indices like haplotype diversity (*H*), nucleotide diversity (*π*), and the ratio of shared haplotypes (*R*_shr_) were also mapped into contours to visualize the spatial pattern of genetic connectivity of the 60 populations of *S. furcifera* across the research range.

### 2.4 Neutrality and IBD analyses

Neutrality tests using Tajima's *D* (Tajima, 1989), Fu's *F*s (Fu, 1997), sum of squared deviations (SSD) (Schneider & Excoffier, 1999), and the raggedness index (RI) (Harpending, 1994) were performed in Arlequin 3.11 on each population with 1,000 iterations to test significance. Significant negative *D* and *F*s values indicate genetic hitchhiking, which might be caused by some events in demographic history (e.g., recent expansion, background selection, or genetic sweeping), while insignificant small SSD and RI values indicate rapid population expansion.

In an attempt to test a null hypothesis and whether geographic distance contributes to the genetic differentiation of *S. furcifera*, an isolation‐by‐distance (IBD) analysis was performed using IBDWS 3.2 (Jensen, Bohonak, & Kelley, 2005). Spherical distances between the 60 sampling sites were measured in ArcGIS 10 with GCS‐WGS‐1984 coordination system and Asia North Albers equal area conic projection. Mantel tests were performed on the genetic distance of pairwise differences (*F*_ST_) against both of the geographic distances as well as the logarithm transformed geographic distances with 10,000 iterations for significance.

### 2.5 Migration and wind analysis

**FIGURE 2:**
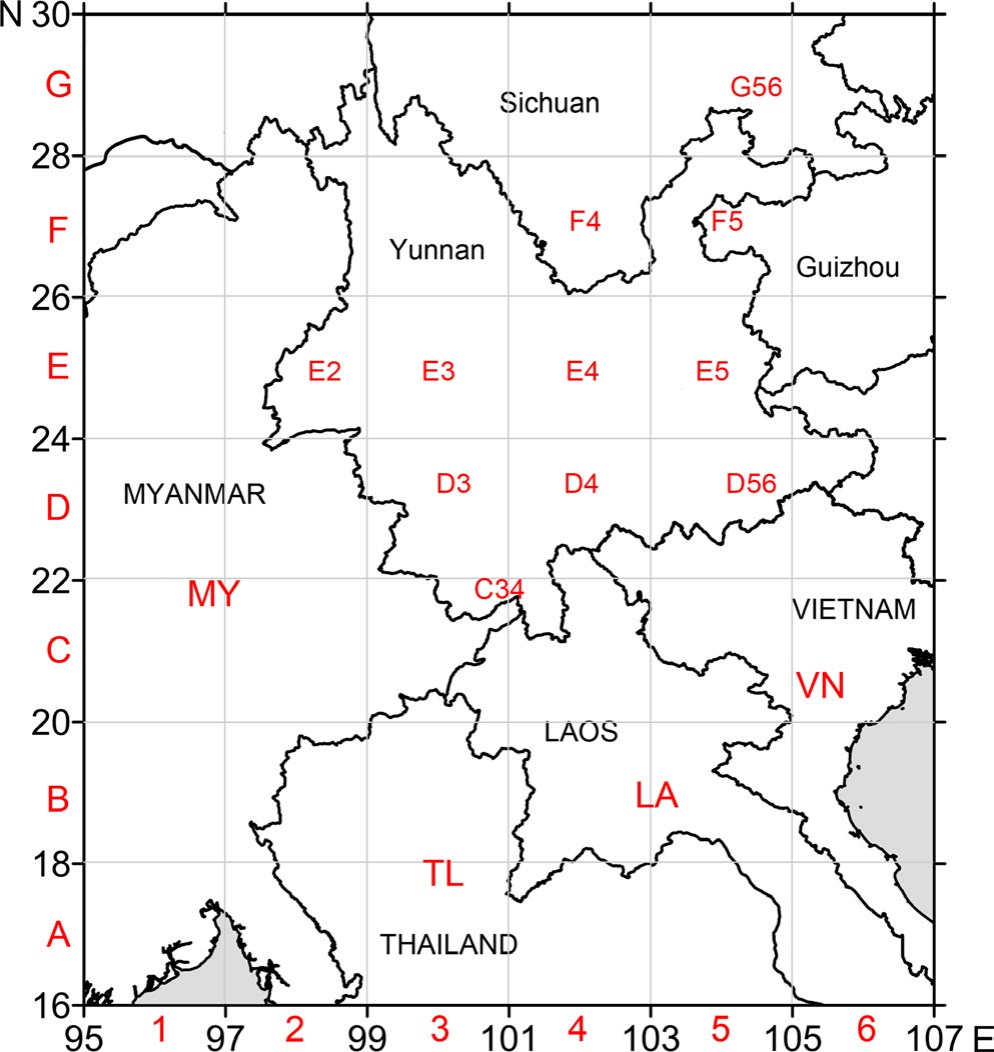
Four Indochinese population groups and 11 domestic population groups used in migration pathways inference. Map is generated by Surfer 10.0

For a better interpretation, the 60 populations were divided into 15 groups by the following criteria: (a) Populations of each Indochinese country across the border constitute an independent group for an easier source identification; (b) populations in China were divided by a two‐degree grid, with those less than half a grid combined into the neighboring ones to form 11 domestic groups (Figure 2). To infer the migration pathways of *S. furcifera* in the study area, migration rates between the 15 population groups were calculated using Migrate 3.5 (Beerli & Felsenstein, 2001) (http://popgen.sc.fsu.edu/oldversions/3.x/3.5/). Groups with large migration rates in between (>1,000) as well as haplotype connections were annotated with an arrow connecting two relevant cells on the map to represent tentative migration pathways.

To determine whether winds facilitate migration pathways detected by previous molecular analysis, wind fields on 850 mb (~1,500 m above sea level [a.s.l.]) and 700 mb (~3,000 m a.s.l.) were plotted using the 30 km resolution ERA5 wind data provided in 2004–2013 by the European Centre for Medium‐Range Weather Forecasts (ECMWF) (Hersbach, 2018) (https://cds.climate.copernicus.eu/). Because the 850 mb wind fields are lower than most elevations of Yunnan Province, our main focus is the wind fields at 700 mb in the present research. Since the wind condition is very complex in the study area, the analyses were performed based on the following reasons and methods: (a) Monthly averaged data were used to reconstruct prevailing winds that determine the main stream migrations that occur frequently (e.g., northward and eastward migrations) (Huang, Chen, Wang, & Lin, 2012; Wei, Zhang, Wen, Kim, & Nam, 2015). (b) The prevailing winds shift from westerly to southwesterly or southerly from June to September, with a reduction in magnitudes and an increase in direction diversity. Since winds at opposite directions within a month would likely cancel each other out in average datasets, the 12‐time slices of the hourly averaged wind fields (on a bihourly basis starts at hour 00) were used to track the direction shifts from June to September in an attempt to detect carrying winds for southward (return) and westward migrations. (c) Rice plantation across the research range differs temporally from the south to the north. In the central and northern parts, rice growing season only starts from late May to late September, when *S. furcifera* infestation occurs (Liu, Yang, Lin, & Kong, 1991). Thus, the present research focuses more on the period from June to September for possible southward (return) migration.

## 3 RESULTS

### 3.1 Spatial pattern of haplotypes and genetic diversity

**FIGURE 3:**
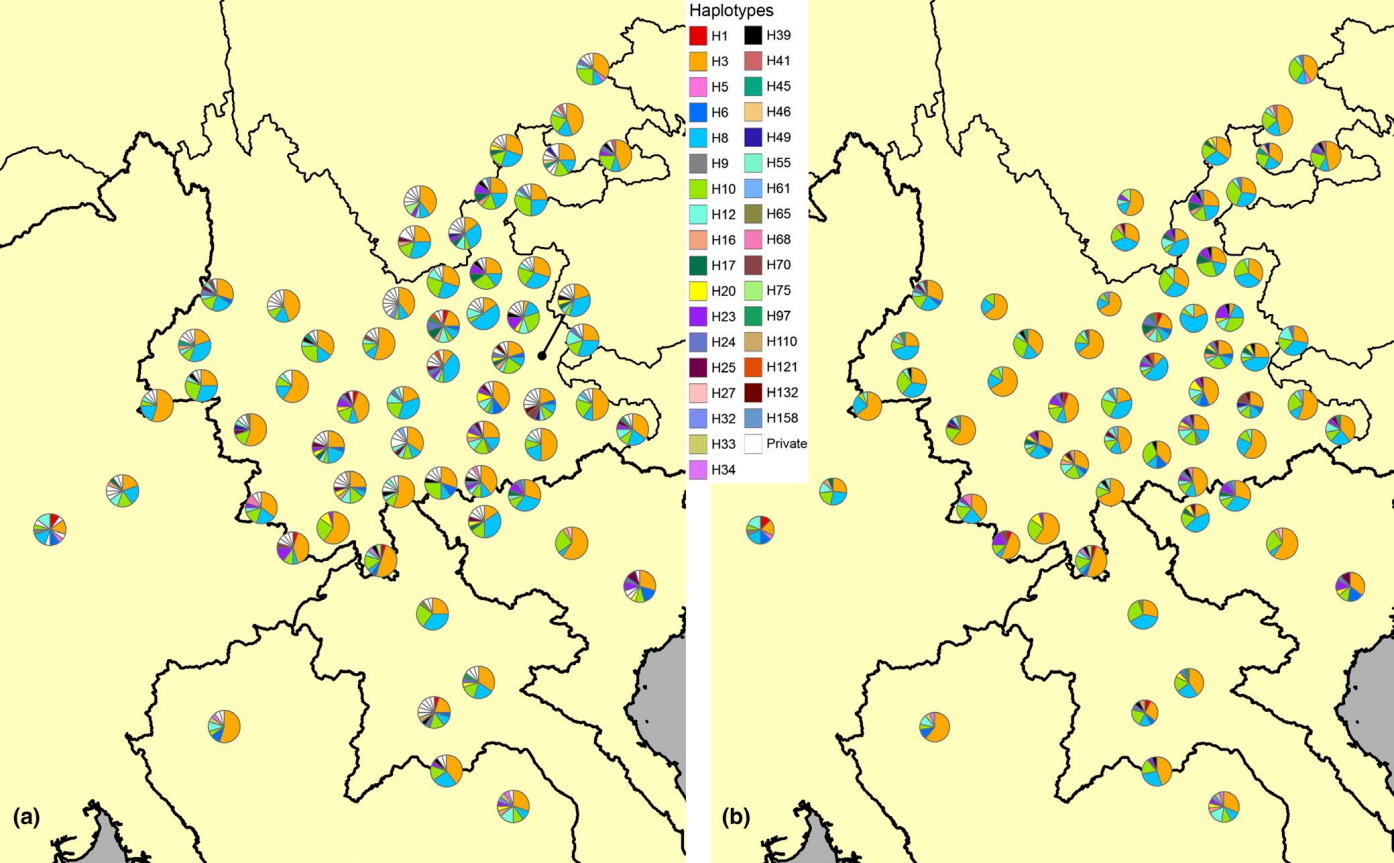
Spatial distribution of (a) all haplotypes and (b) shared haplotypes of the 60 populations of *S. furcifera*, circle sizes correspond to the number of samples. Map and statistics generated by ArcGIS 10.0

In total, 205 haplotypes were identified, with 34 shared haplotypes and 171 private haplotypes, the ratio of shared haplotypes (*R*_shr_) ranged from 0.33 to 1.00, with both of Yuanmou (No. 44) and Yangbi (No. 45) of Yunnan being the minimum and Yen Bai, Vietnam (No. 10), Mengla (No. 12), Jinghong (No. 14), and Hekou (No. 16) of Yunnan all being the maximum (Table S1). The abundance of haplotypes (*h*) in each population ranged from 4 to 14, with Jinghong, Yunnan (No. 14) being the minimum and both of Vang Vieng, Laos (No. 4) and Qiubei, Yunnan (No. 30) being the maximum; most of the remaining populations (78.9%) possessed 7 to 11 haplotypes (Table S1). Among all 34 shared haplotypes, H3, H8, and H10 were three dominant haplotypes found in all of the 60 populations, while the remaining shared haplotypes showed different extent of regional endemism (Figure 3). The details of haplotype diversity (*H*), ratio of shared (*R*_shr_) and private (*R*_prv_) haplotypes, nucleotide diversity (*π*), K2P genetic distance, pairwise *F*st values, and Nei's average differences between populations are listed in Tables S1–S3.

**FIGURE 4:**
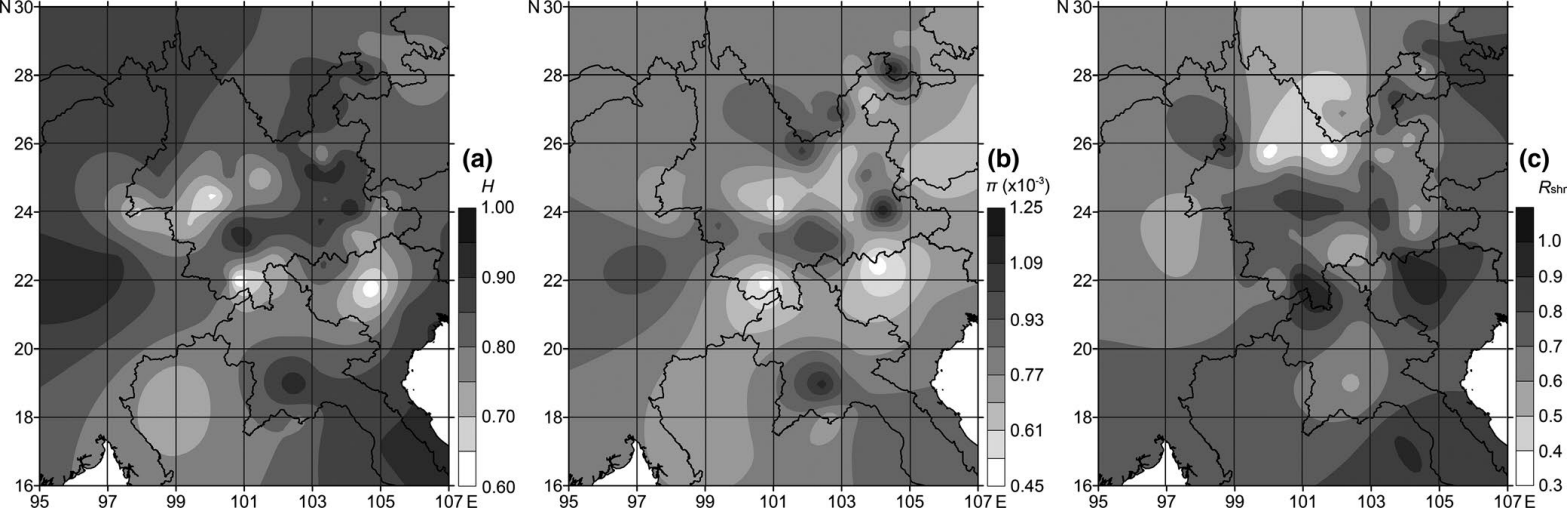
Spatial distribution of (a) haplotype diversity, (b) nucleotide diversity, and (c) the ratio of shared haplotypes of the 60 populations of *S. furcifera*. Maps generated by Surfer 10.0

The spatial distribution analyses of haplotype diversity (*H*) showed a continuous band of higher diversity from southwestern Yunnan to northeast Yunnan and southern Sichuan (D3, D4, D56, E5, E5, F4, F5, and G56), with three high diversity centers. Meanwhile, three low diversity centers were identified in southern Yunnan (C34, D3, and D4), from western to central Yunnan (E2, E3, and E4), and in the junction of northern Vietnam (VN) and southeastern Yunnan (D56) (Figure 4a). For nucleotide diversity (*π*), the spatial distribution showed a roughly similar pattern with that of *H*, while the high diversity band across Yunnan in a southwestern to northeastern direction was interrupted by two lower diversity patches in central and northeastern Yunnan (mainly in E3, E4, and F5) (Figure 4b). In contrast, spatial distribution of the ratio of shared haplotypes (*R*_shr_) showed higher shared ratio in southern Yunnan (C34), northern Vietnam (VN), the southern margin of central Yunnan (mainly E3, E4, E5, and D56), and northeastern Yunnan to southern Sichuan (F5 and G56), while the shared ratio in central Myanmar (MY) and the western corner of Yunnan (E2), northern Laos (LA), central Yunnan to southern Sichuan (E3, E4, and F4), and eastern Yunnan (E5) was lower (Figure 4c).

### 3.2 Neutrality and IBD tests

Neutrality tests with both Tajima's *D* and Fu's *F*s methods showed significant negative values for the entire 60 populations, with small insignificant values SSD and insignificant RI values (Table S4). For each of the 60 populations, our neutrality test obtained 46 negative Tajima's *D* value (76.7%) with only 2 of them being significant (3.3%), while the test obtained 59 negative Fu's *F*s values (98.3%) with 48 of them being significant (80.0%) (Table S4). Most SSD values were insignificantly small, except only one for Ruili (No. 28) being large and significant, all RI values were insignificant (Table S4). The extensive significant negative neutrality test values (especially Fu's *F*s values) coupled with insignificant SSD values and raggedness indices suggested almost all sampled populations of *S. furcifera* underwent a rapid population expansion event.

**FIGURE 5:**
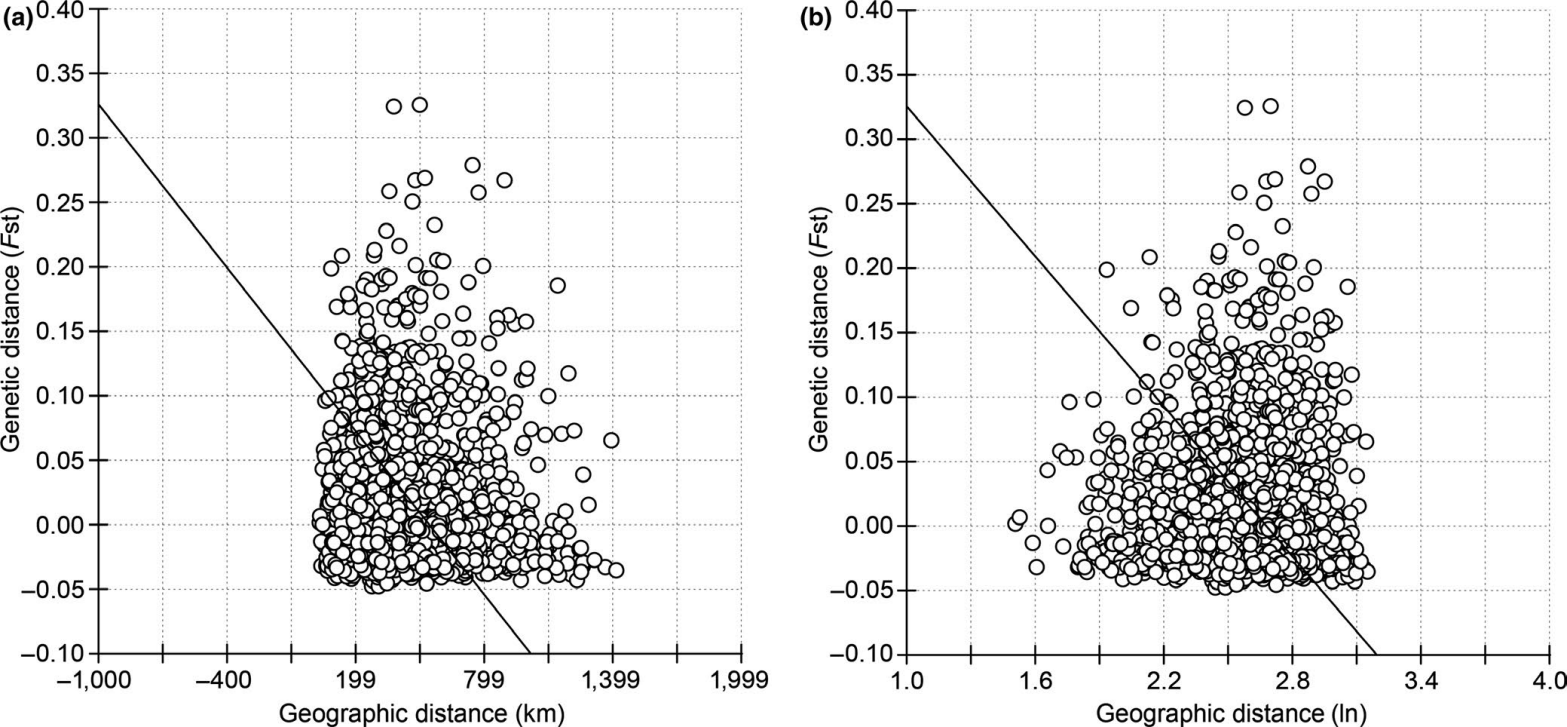
Plots of the IBD tests generated by IBDWS 3.2 and Grapher 8.0, (a) regression of pairwise differences (*F*_ST_) against the geographic distances (Mantel *r* = −.0347, *p* = .680, with 10,000 iterations), and (b) regression of pairwise differences (*F*_ST_) against the logarithm transformed geographic distances (Mantel *r* = −.0051, *p* = .504, with 10,000 iterations). Charts are generated by Grapher 8.0

The IBD test showed that there was no regression correlation between the pairwise differences (*F*_ST_) of the 60 populations of *S. furcifera* and either the geographic distances between the 60 sampling sites or their logarithm transformed values (Figure 5). The uncorrelated result indicated that geographic distances/barriers in Yunnan are not significant enough to introduce genetic differentiations between *S. furcifera* from different places.

### 3.3 Migration pathways

**FIGURE 6:**
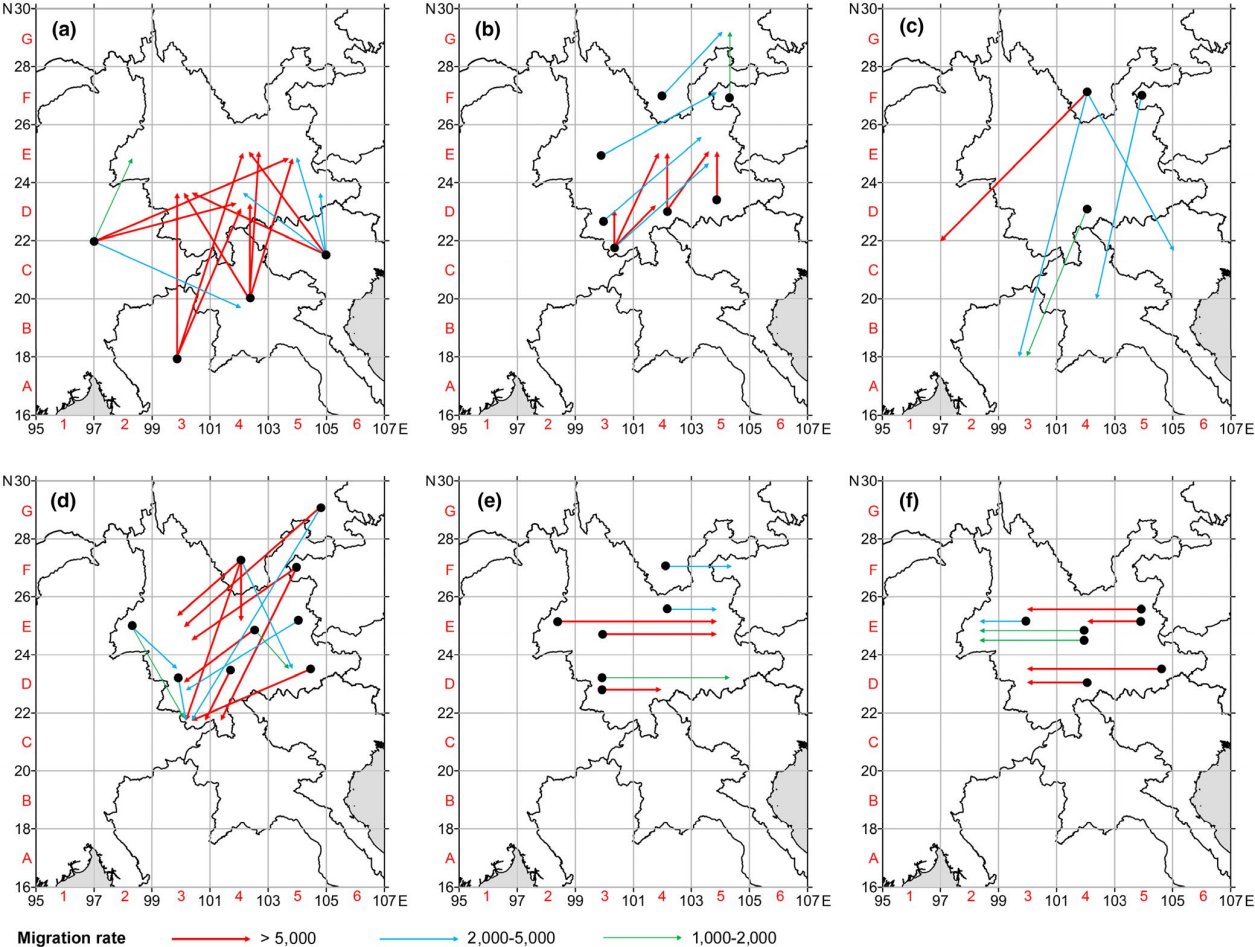
Putative migration pathways of *S. furcifera* in Yunnan and between its neighbors. (a) Northward transboundary migration from Indochinese countries to Yunnan and Sichuan, as well as between Indochinese countries; (b) northward domestic migration within China; (c) southward (return) transboundary migration from Yunnan and Sichuan to Indochinese countries; (d) southward (return) domestic migration within China; (e) eastward migration; and (f) westward migration. Black circles denote source grids without indicating precise positions, and the arrows denote destination grids without indicating precise bearing. Maps generated by Surfer 10.0

Migration analysis identified 16 large immigration rates and six large emigration rates between the four Indochinese countries (Table S5), and it is noteworthy that the frequencies of large migration rates were higher in transboundary northward migration and domestic southward (return) migration than domestic northward migration and transboundary southward (return) migration, respectively. These migration rates were illustrated on the map to reveal the spatial pattern of inferred migration pathways. All illustrated migration pathways were categorized into transboundary northward migration (Figure 6a), domestic northward migration (Figure 6b), transboundary southward (return) migration (Figure 6c), domestic southward (return) migration (Figure 6d), and domestic eastward and westward migrations (Figure 6e,f).

The most influenced transboundary northward migration destination area and the probable source areas are the following: (a) from Myanmar (MY) to western Yunnan (E2), southern Yunnan (D4), and eastern Yunnan (E5); (b) from Thailand (TL) to southern Yunnan (D4), southwestern Yunnan (D3), western Yunnan (E2), and central Yunnan (E4); (c) from Laos (LA) to southern Yunnan (D3 and D4), southwestern Yunnan (D3), central Yunnan (E4), and eastern Yunnan (E5); and (d) from Vietnam (VN) to southeastern Yunnan (D56), southern Yunnan (D3 and D4), eastern Yunnan (E5), and southwestern Yunnan (D3) (Figure 6a).

Most transboundary southward (return) migration to Indochinese countries occurred: (a) from southern Sichuan (F4 and G56) to Myanmar (MY), Thailand (TL), and Vietnam (VN); (b) from northeastern Yunnan (F5) to Laos (LA); (c) from western Yunnan (E2 and E3) to Myanmar (MY) and Vietnam (VN); and (d) from southern Yunnan (D4) to Thailand (TL) (Figure 6c).

Domestic migration happens in four directions, namely northward, eastward, southward (return), and westward. Most northward migration originated in the southern bordering areas in Yunnan (C34, D3, D4, and D56) and destined to the central and eastern parts (E4 and E5) in a northeast direction. Similarly, those originated in the west‐central (E3 and E4) area destined to the northeastern part of Yunnan and the southern part of Sichuan (F5 and G56) in the same direction (Figure 6b). Most southward (return) migration occurred from southern Sichuan and the northeastern part of Yunnan (F4, F5, and G56) to western, central and southeastern parts of Yunnan (E2, E3, E4, and G56), and again to the southern parts of Yunnan (C34, D3, and D4) (Figure 6d). The eastward migration occurred less frequently, only a few pathways inferred from southwestern Yunnan (D3 and D4) to southeastern Yunnan (D56), from western‐central Yunnan (E2, E3, and E4) to eastern Yunnan (E5), and from southern Sichuan (F4) to northeastern Yunnan (F5 and G56) (Figure 6e). The westward migration roughly mirrored the eastward migration (Figure 6f).

### 3.4 Carrying winds

**FIGURE 7:**
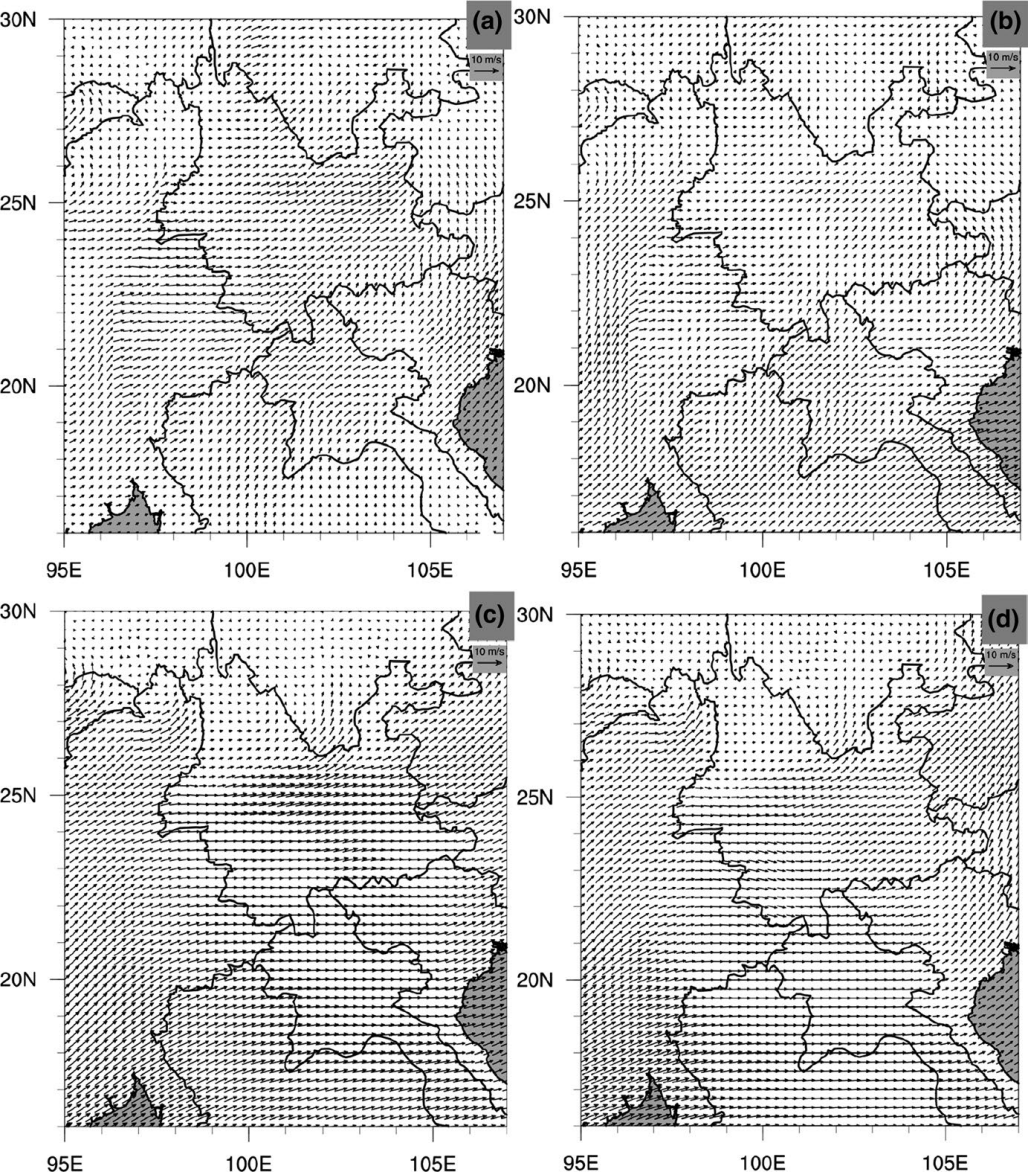
Examples of wind fields carrying northward and eastward migrations of *S. furcifera*. Monthly prevailing southwesterly and southerly winds favoring northward migration (850 mb) averaged across the hour 06 of each day in March (a) and May (b) from 2004 to 2013. Monthly regularly occurring westerly winds supporting eastward migration in summer (700 mb) averaged across the hour 00 of each day in June (c) and July (d) from 2004 to 2013

**FIGURE 8:**
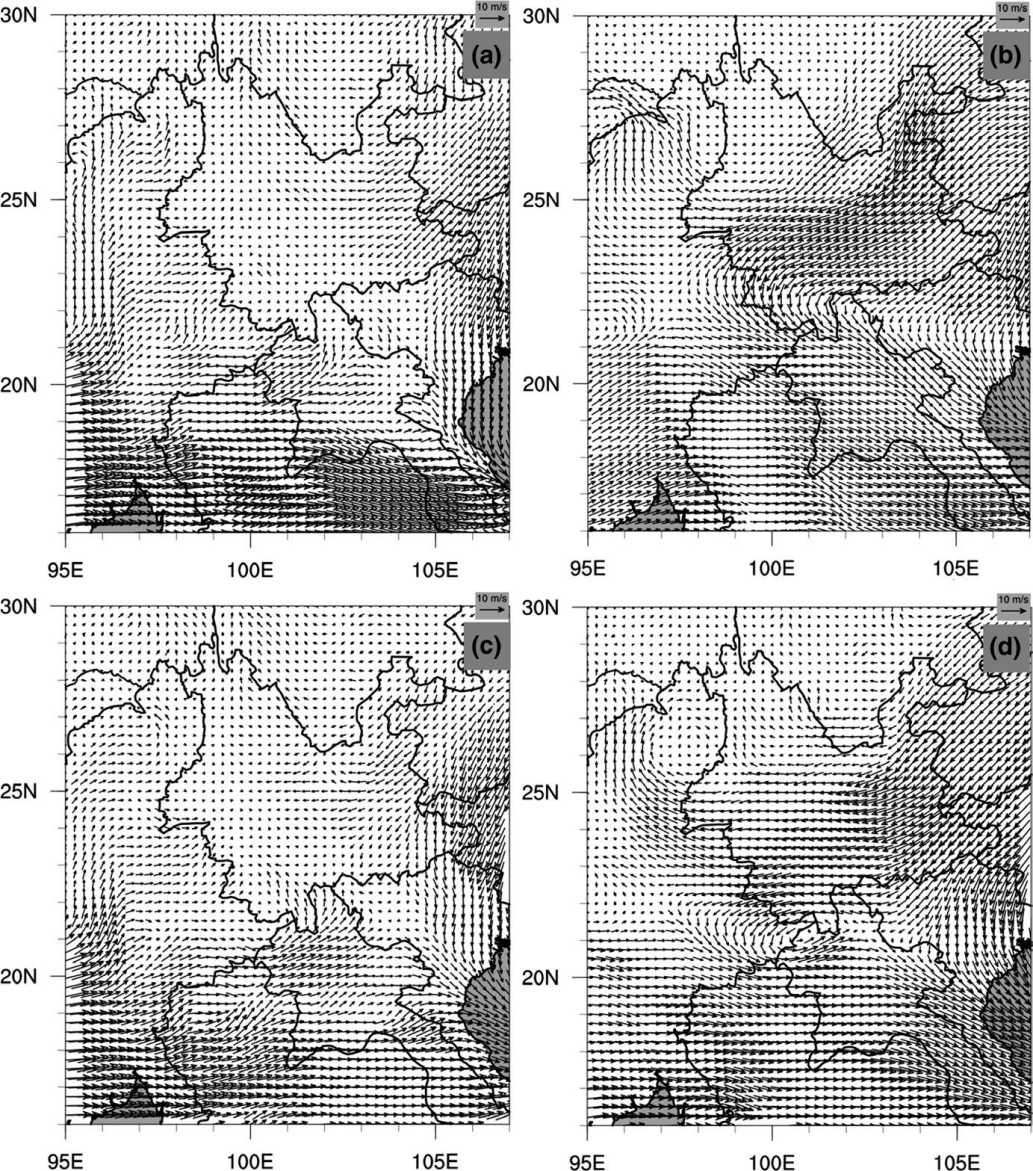
Examples of hourly averaged wind fields carrying southward (return) and westward migrations of *S. furcifera*. (a and b) show northerly winds carrying southward (return) migration from June to September (850 mb and 700 mb), and (c and d) show easterly winds supporting westward migration from June to September (850 mb and 700 mb). All four subplots are from the data for year 2009. (a) hourly averaged 850mb wind filed at hour 9 on August 08; (b) represents hourly averaged 700 mb wind filed at hour 19 on August 05; (c) hourly averaged 850 mb wind filed at hour 9 on August 06; (d) represents hourly averaged 700 mb wind filed at hour 13 on August 06

**FIGURE 9:**
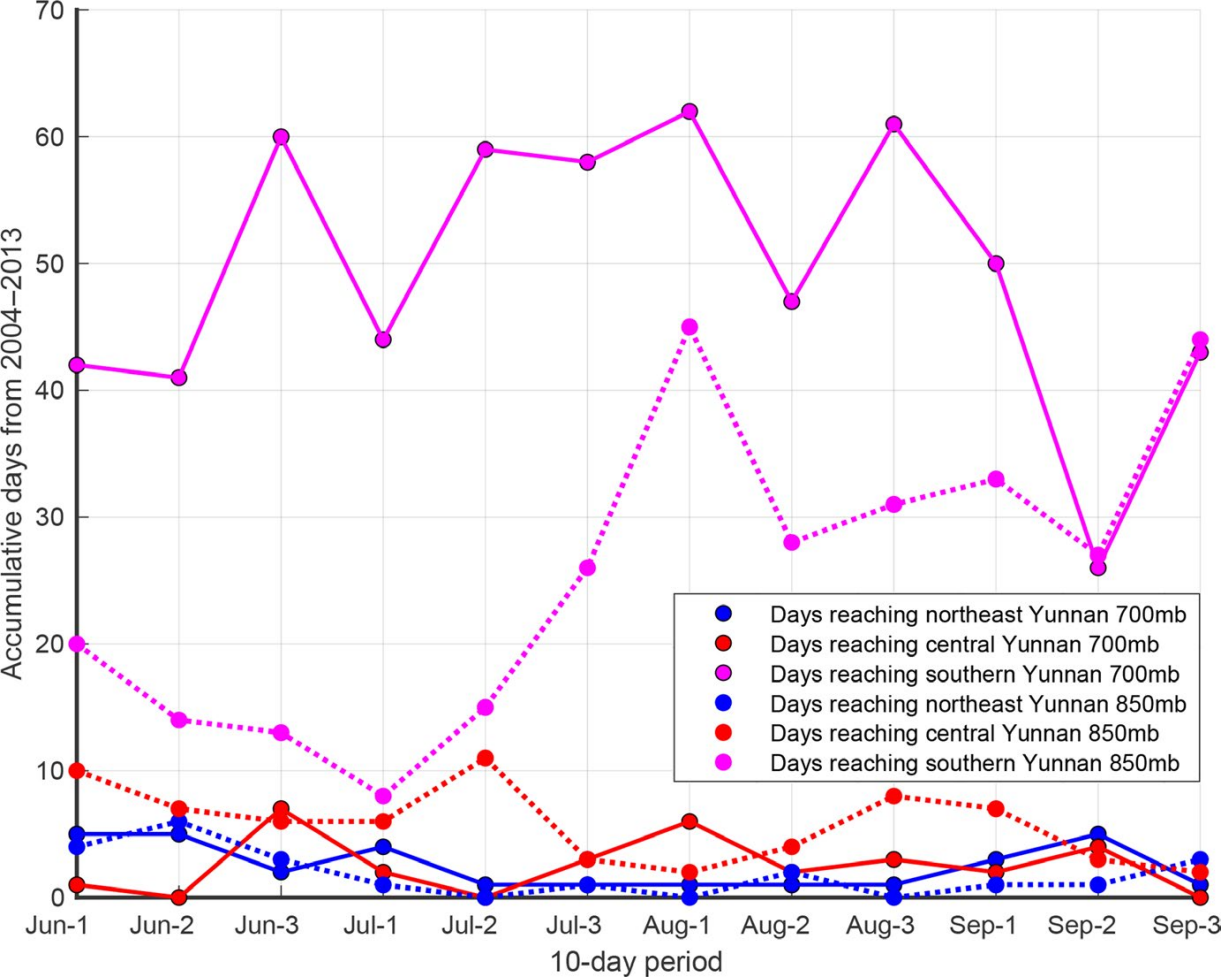
During the 1st, the 2nd and the 3rd 10 days from June to September in 10 years (2004–2013) at 700 mb and 850 mb, the accumulative northerly windy days reaching northeast Yunnan (areas in row F in Figure 2) are shown in blue filled circles, reaching central Yunnan (areas in row E in Figure 2) in red filled circles, and reaching southern Yunnan (areas in rows C and D in Figure 2) in purple filled circles. Solid lines represent accumulative windy days at 700 mb, and dashed lines represent accumulative windy days at 850 mb

**FIGURE 10:**
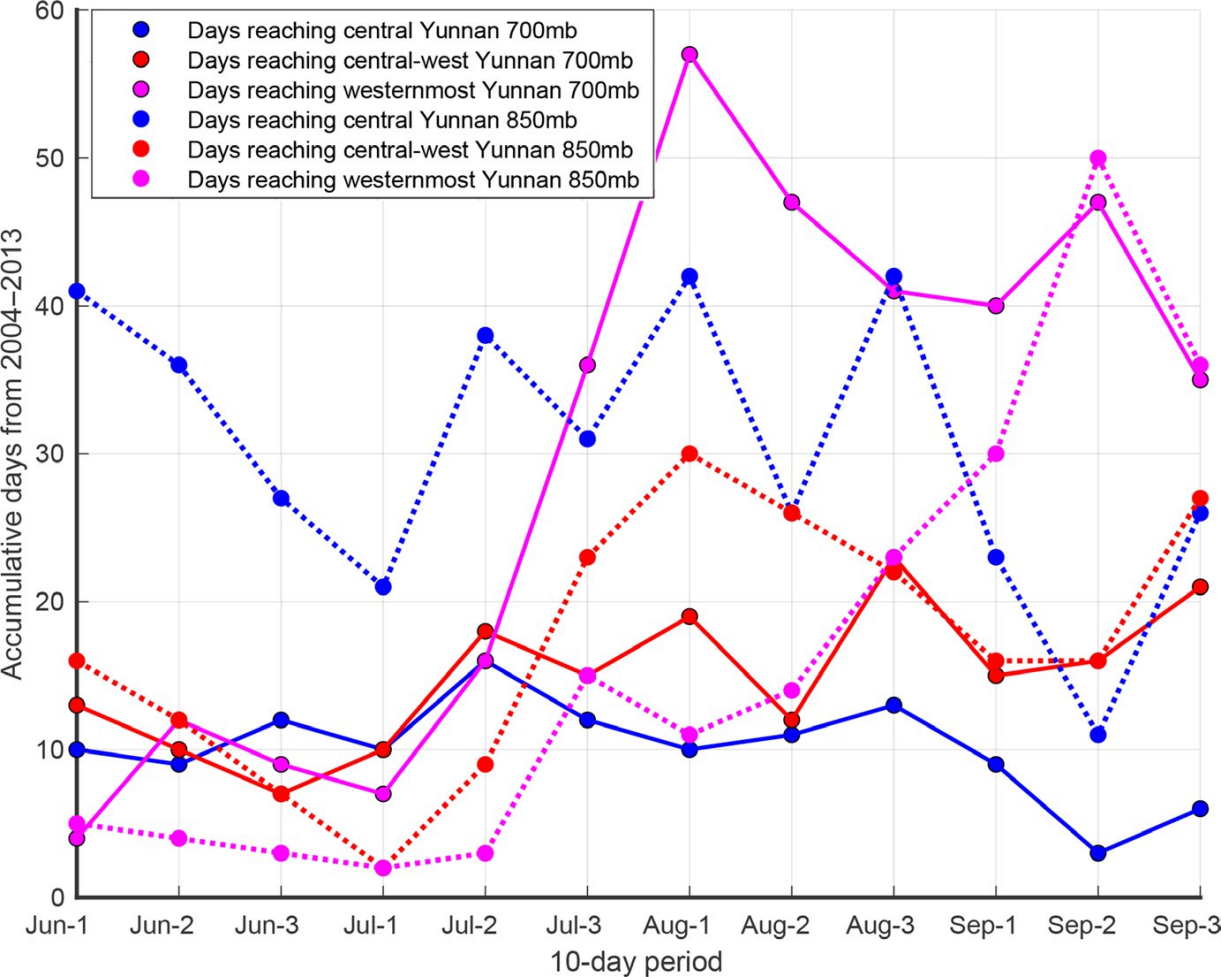
During the 1st, the 2nd and the 3rd 10 days from June to September in 10 years (2004–2013) at 700 mb and 850 mb, the accumulative easterly windy days reaching central Yunnan (areas in column 4) are shown in blue filled circles, reaching central‐west Yunnan (areas in column 3) in red filled circles, and reaching westernmost Yunnan (areas in column 2 and westward of Figure 2) in purple filled circles. Solid lines represent accumulative windy days at 700 mb, and dashed lines represent accumulative windy days at 850 mb

Wind analyses based on monthly averaged data (Figures [Link], [Link], [Link], [Link], [Link], [Link], [Link], [Link], [Link], [Link]) demonstrated that the prevailing southwesterly and southerly currents agreed with the aforementioned northeast‐ and northward migration pathways and directions of *S. furcifera* in Yunnan throughout a year (Figure 7a,b); regularly occurring westerly winds detected in summer seasons also agreed with the eastward migration in Yunnan (Figure 7c,d). However, the signals of northerly and easterly winds are obscured by the southerly and westerly winds, which dominate the monthly averages because of their higher frequencies and larger magnitudes. Therefore, our analyses used the hourly averaged ERA5 wind data of every two hours to dig out the bursts of northerly and easterly winds that could well act as the air carriers to facilitate the southward (return) (Figure 8a,b) and westward (Figure 8c,d) migrations, covering different regions of Yunnan from June to September. Overall, the transprovincial winds are the most frequently occurring carrying winds in the 10 years, with the southerly winds most active from late June to early September, and the easterly ones most active from late July to late September (Figures 9 and 10; Figures S11 and S12).

## 4 DISCUSSION

### 4.1 Advantage of combining molecular and meteorological data

Revealing migration pathways is always an important task in rice planthopper research. The geographically complex area centered by our research range is more challenging to reveal the pattern. However, the challenge of such a task can on the one hand largely be attributed to the temporal discrepancy between the molecular characters formed over the evolutionary history and the commonly used meteorological simulation focusing on particular migration events (Bi et al., 2014; Otuka et al., 2010, 2012; Sun et al., 2018).

On the other hand, given the very high genetic connectivity among populations of migratory rice planthoppers, population genetic structure may well be dissolved; hence, routine analyses like phylogenetic trees, structure clustering, and SAMOVA are less effective in concluding a migration pathway (Liu et al., 2010; Sun et al., 2014; Xie et al., 2014). Similar results have also been widely seen in various planthoppers, armyworms, grasshoppers, and plant bugs (Arias et al., 2019; Blanchet et al., 2012; Chapuis et al., 2009; Golikhajeh, Naseri, Razmjou, Hosseini, & Aghbolaghi, 2018; Matsumoto et al., 2013; Mun, Song, Heong, & Roderick, 1999; Nagoshi, Fleischer, et al., 2018; Nagoshi, Goergen, et al., 2018; Nam et al., 2018; Qiu et al., 2016; Sun et al., 2015; Zhang et al., 2017). A logical explanation for this phenomenon is the strong fecundity and mobility of these pests. When a large swarm of pests with strong mobility overcomes most environmental obstacles in a research range (e.g., terrain barriers, climate shifts, and discontinuous host distribution) the genetic divergence between populations would be diminished by high rates of gene flow (Figure 5; Table S2).

Insect migration is an evolutionary pattern gained over time under regular driving forces in a given space, and the evolutionary footprint of such pattern should be retrievable using molecular analysis, which is the internal evidence. When single or conventional analysis cannot fully reveal the pattern, analyses focusing more on the connection among populations, such as haplotype distribution, spatial pattern of diversity indices, and migration rate, need to be involved. Furthermore, external evidence and driving forces of the migration must also be utilized to cross‐validate the possible migration pattern identified by molecular analyses.

By using the aforementioned molecular analytical methods together, the present study was able to summarize a collection of putative migration source areas and pathways. After combining the analysis of 10‐year winds representing long‐term migration driving forces, the migration pathways were not only supported by large‐scale circulation patterns, but can also be explained in terms of likely occurring periods when taking rice plantation and the distribution of *S. furcifera* into consideration. Apart from the findings and conclusions, the present study could also provide new insights to solve the analytical difficulties in other migratory pests.

### 4.2 Migration sources and pathways

Our analyses confirmed the mainstream north and northeastward migrations originated from four source "belts" in the research range, northern Indochina, southern Yunnan, central Yunnan, and northeastern Yunnan (Figure 6a,b), in accordance with the commonly recognized prevailing winds governed by both of the Indian and Pacific monsoons (Hu et al., 2014, 2018; Ma et al., 2018). The transboundary immigration originating from northern Indochina mostly impacts southern Yunnan but can reach as far as the central Yunnan altiplano in the east (Figure 6a), similar to the major findings regarding the possible sources and landing areas reported by Shen et al. (2011) and Li et al. (2016). With the increase of latitude, the migration frequency decreased gradually and noticeably toward the northeastern corner of Yunnan and southern Sichuan (Figure 6b); geographic barriers such as elevating altitudes on the Central Yunnan altiplano and the major mountain ranges may explain such decrease.

It is noteworthy that concurring southward (return) migrations were detected by both migration analyses (Figure 6c,d) and supported by wind field analysis (Figure 8c,d), with the four previously mentioned "belts" being the sources in a reversed order. Numerical experiments have indicated that the frequent bursts of southward winds might be attributable to the Yunnan–Guizhou Plateau's mechanical blocking of the monsoon circulation in June to August (Shi et al., 2017; Shi, Sha, Liu, Xie, & Li, 2019). Therefore, in addition to the northward main flows, the topography‐induced anomalous southward air circulations can facilitate the insects’ migrations in almost all directions in the growing season. Although long‐range transboundary southward (return) migrations are rare, short‐range domestic southward (return) migrations are quite frequent and strong, covering the entire rice growing part of Yunnan (Figure 6d).

Apart from northward and southward migrations, our analyses also detected eastward and westward migrations across Yunnan (Figure 6e,f), with eastward migrations more common than westward ones, which only occurred in certain periods of time. The eastward migrations are carried by a common division of the prevailing winds governing the research range, while the westward air flows are induced by the same mechanism as that of the southward ones (Shi et al., 2017, 2019), Compared to the dominant northward and the regular southward (return) migrations, these two directions are less frequent. However, they are also capable of carrying *S. furcifera* across Yunnan, as reported in a previous study by Shen et al. (2016).

### 4.3 Relationship between migration and outbreaks

The southern part of Yunnan is the earliest and continuously devastated area by *S. furcifera* every year, and the continuous breeding of *S. furcifera* populations in overwintering areas (Hu, Fu, et al., 2015; Hu, Liu, et al., 2015) coupled with high immigration rates from northern Indochina (Figure 6a) may well explain the heavy outbreaks occurring in this area, particularly near the border (Gui et al., 2008; Hu, 2013).

As mentioned above, the northward migrations reduce with the increasing latitudes; however, the eastern and northeastern parts of Yunnan as well as southern Sichuan are not alleviated from infestation of *S. furcifera* (Bi et al., 2014; Hu et al., 2019; Li, Yin, Zhao, Zhai, & Chen, 2017; Liu et al., 1991; Xiang et al., 2015; Xue, Jin, & Yang, 2014; Zhao & Li, 2015). Such contradiction could be logically explained by the presence of concurring southward (return) migrations: this part of Yunnan in question receives *S. furcifera* migrants from both the south and the north in the research range. On a larger scale, the northeast corner of Yunnan and southern Sichuan could highly likely receive migrants from further north carried by the same wind in summer, thus making the impact also heavy compared to south Yunnan.

Our finding of concurring southward (return) migration not only explained the heavy impacts in eastern and northeastern Yunnan, but also is more logical in terms of host availability. If *S. furcifera* could only return to the south after autumn as conventionally understood (Riley et al., 1991; Wu et al., 2019; Zhao & Li, 2015), the host availability in most single‐seasoned planting areas of Yunnan would be rather poor due to deteriorated nutrient quality of rice plants near harvesting (Denno & Roderick, 1990). Given such unfavorable host availability, *S. furcifera* could hardly maintain a population size which can be detected in the present research using molecular techniques.

### 4.4 Management implications

Our analyses showed that the southern part of Yunnan and the southern margin of central Yunnan (e.g., Xishuangbanna, Lincang, Pu'er, Honghe, Yuxi, and Wenshan) are the first impacted areas when a northward transboundary migration occurs (Figure 6a). After reaching, this area *S. furcifera* will continue further north into central Yunnan and Sichuan (Figure 6b). Effective monitoring and forecasting of the population dynamics of *S. furcifera* in this frontier area is essential for subsequent successful pest management. However, the southern margin of this area does not only receive immigrants from northern Indochina, but also harbors overwintering populations of *S. furcifera* in average years (Hu, Fu, et al., 2015; Hu, Liu, et al., 2015). That is to say, the population number of planthoppers in this area is the superimposition of immigrating as well as local populations. Hence, suppressing overwintering *S. furcifera* populations could benefit pest management. With the presence of concurring southward (return) migration, outbreaks and yield loss on the late‐season rice were reported (Hu, Ma, Tang, Pan, & Wang, 1992; Li, Xie, & Lü, 2009; Liu et al., 1991), so a monitoring and management strategy initiated from the north to the south is also necessary to form a better pest management network.

Repeated outbreaks and resurgence are two difficult issues in the management strategies for planthoppers (Catindig et al., 2009). Our haplotype analyses showed that the ratio of private haplotypes was effectively lower at most sampling sites in the four Indochinese countries compared to those in China, while such ratio is much higher in eastern Yunnan compared to the western part (Table S3; Figures 3 and 4c). Most private haplotypes were defined by singletons or a very limited number of nucleotide variations in the sequences, reflecting genetic mutation associated with founder's effect, release of selective stress, and recent expansion (Joy et al., 2003; Lee, 2002), as supported by our results of neutrality tests (Table S4). For *S. furcifera*, periodical pesticide application and agricultural management involving pesticides and herbicides may play an important role in shaping such a genetic profile. With its strong fecundity and rapid growth rate, survivors of *S. furcifera* after eradications would be able to restore the population size in a short period of time, bringing in a large number of mutations (Cheng, 2015; Sogawa, 2015) which might be the potential source of resistance (Liu et al., 2015; Matsumura et al., 2017).

## CONFLICT OF INTEREST

The authors declare no conflict of interest.

## AUTHOR CONTRIBUTIONS

**Shao‐Ji Hu:** Formal analysis (lead); Investigation (lead); Visualization (equal); Writing‐review & editing (equal). **Shan‐Shan Sun:** Formal analysis (equal); Visualization (equal); Writing‐original draft (equal). **Da‐Ying Fu:** Investigation (equal); Resources (equal). **Jian‐Ping Lu:** Investigation (equal); Resources (equal). **Xue‐Ying Wang:** Investigation (equal); Methodology (equal). **Yan‐Ping Yu:** Investigation (equal); Methodology (equal). **Li‐Min Dong:** Investigation (equal); Methodology (equal). **Sui‐Yun Chen:** Conceptualization (equal); Funding acquisition (equal); Resources (equal). **Hui Ye:** Conceptualization (equal); Funding acquisition (equal); Resources (equal).

### OPEN RESEARCH BADGES

This article has earned an Open Data Badge for making publicly available the digitally‐shareable data necessary to reproduce the reported results. The data is available at https://cds.climate.copernicus.eu/.

## Supporting information

Figure S1

Figure S2

Figure S3

Figure S4

Figure S5

Figure S6

Figure S7

Figure S8

Figure S9

Figure S10

Figure S11

Figure S12

Table S1

Table S2

Table S3

Table S4

Table S5

## Data Availability

DNA sequences were deposited on haplotype basis in GenBank, *cox1* gene accession numbers: MN727397–MN727601; *16s* gene accession numbers: MN727602–MN727806. Genetic diversity indices, K2P genetic distances, pairwise *N*m values, neutrality test results, and migration rates were uploaded as supporting information.
