# Supplementary figures and images for: Migration sources and pathways of the pest species Sogatella furcifera in Yunnan, China, and across the border inferred from DNA and wind analyses
Source: Ecol Evol. 2020 Jul 17;10(15):8235–50. doi: 10.1002/ece3.6531 (PMC7417236; doi:10.1002/ece3.6531)

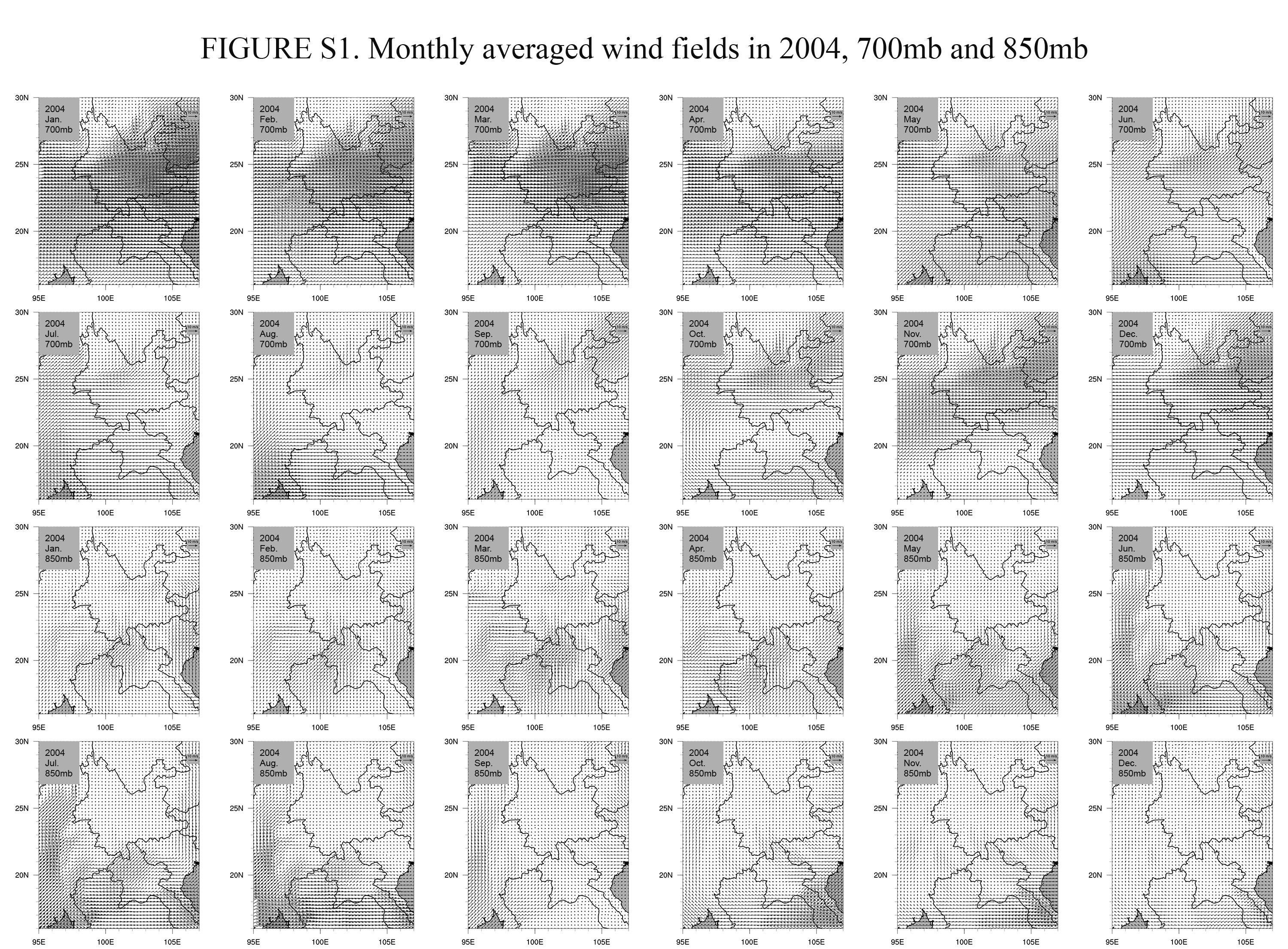

Supplement: Supplementary file 1 — Figure S1 [file ECE3-10-8235-s001.tif]

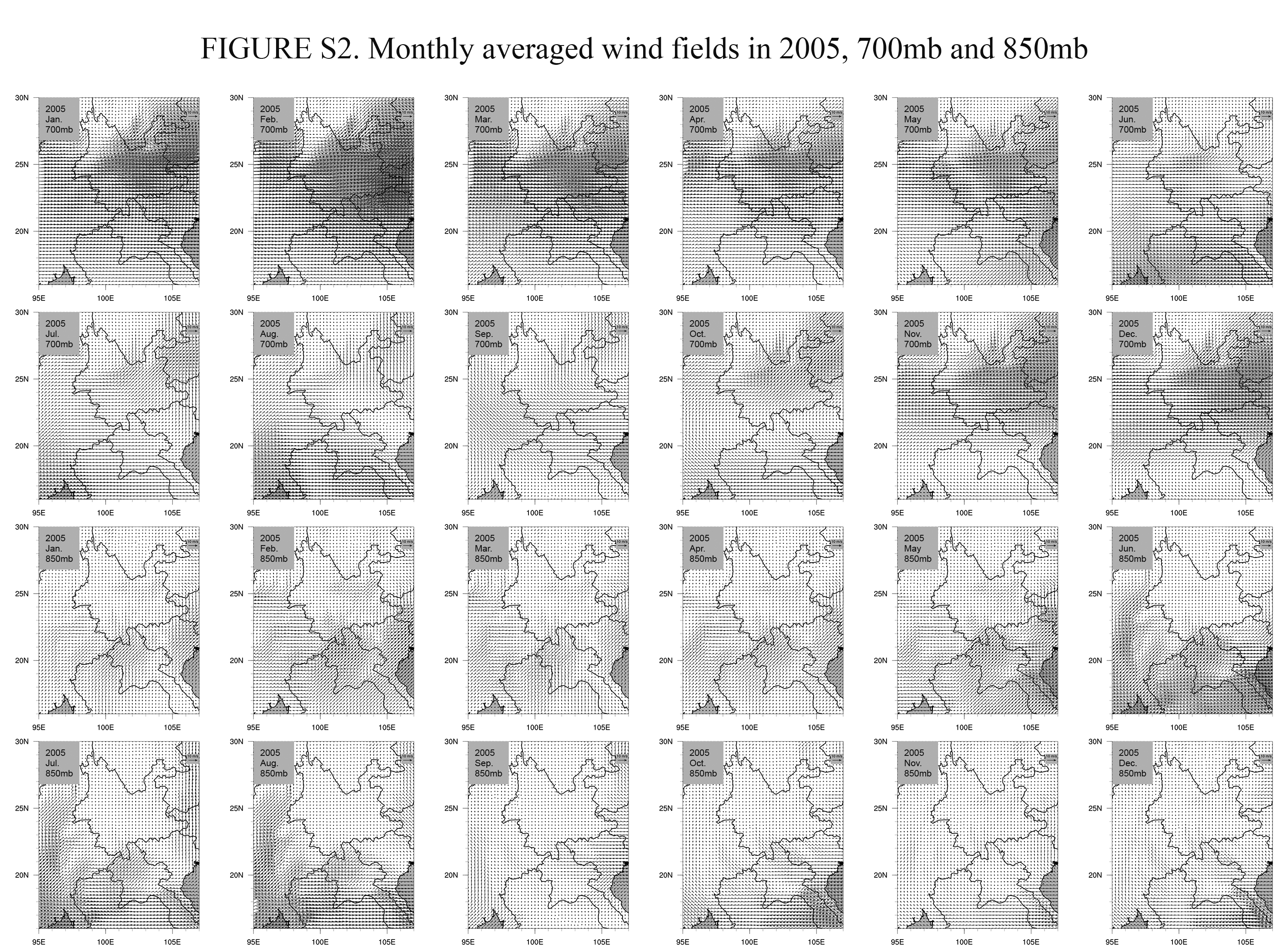

Supplement: Supplementary file 2 — Figure S2 [file ECE3-10-8235-s002.tif]

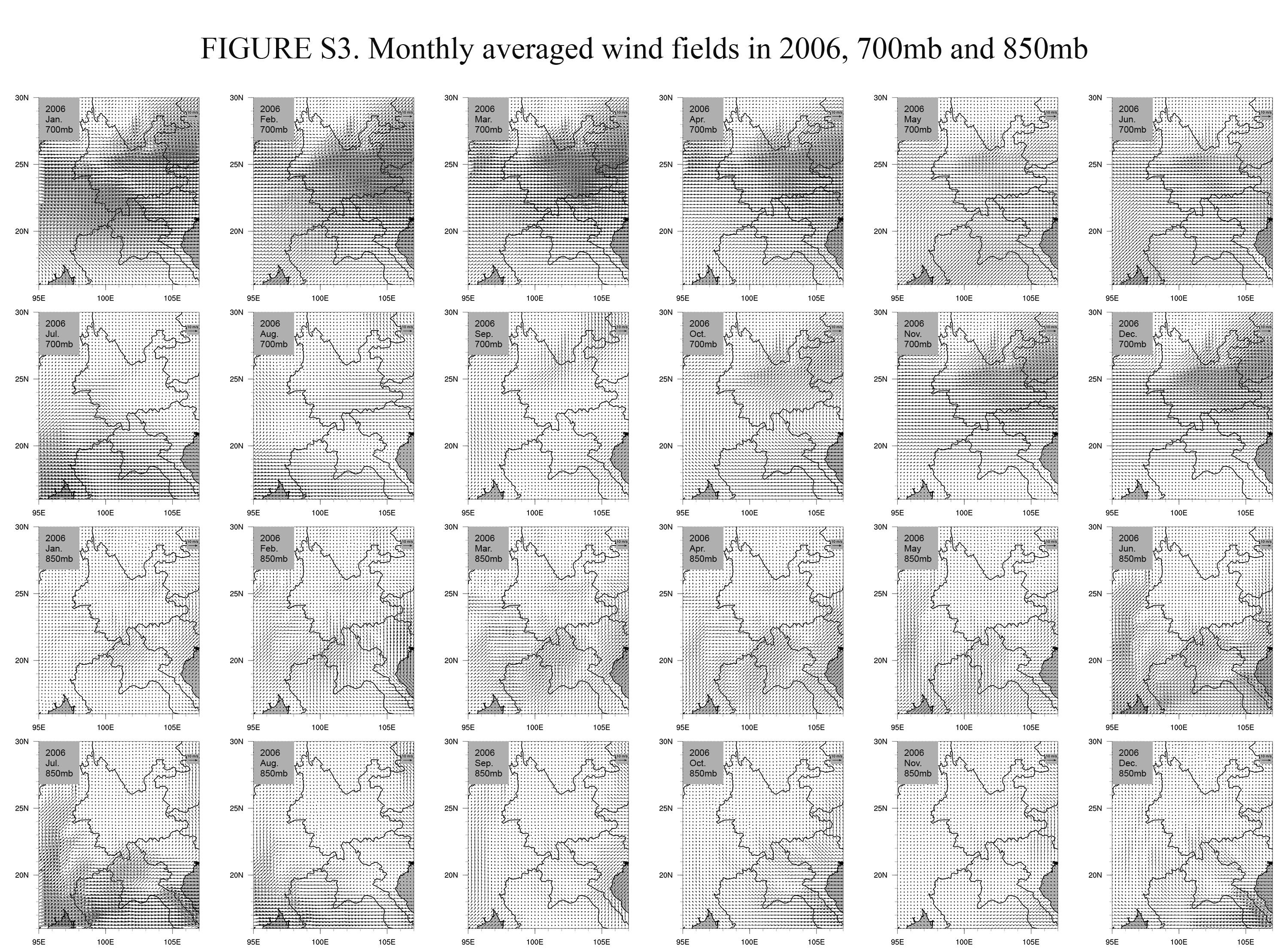

Supplement: Supplementary file 3 — Figure S3 [file ECE3-10-8235-s003.tif]

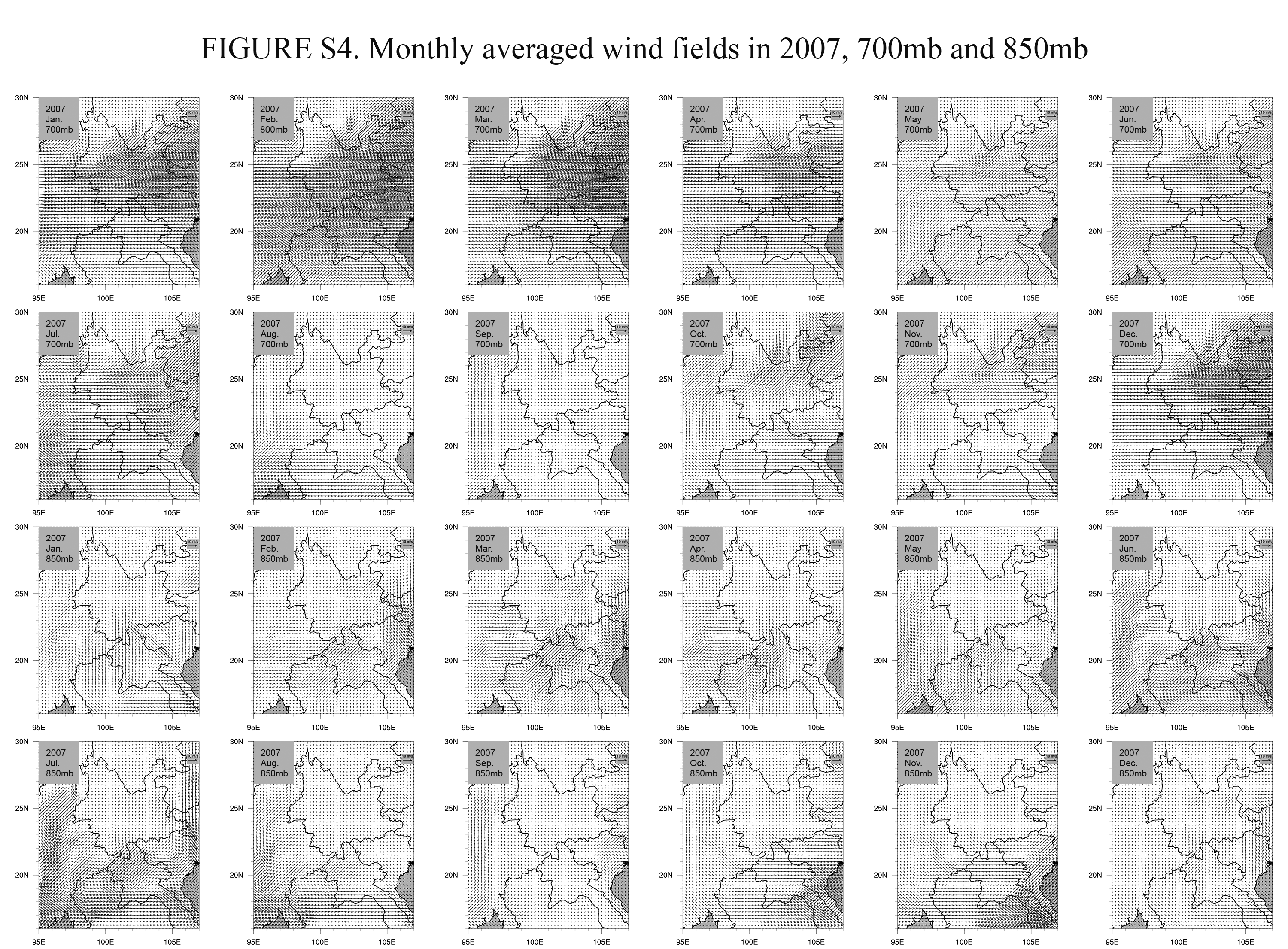

Supplement: Supplementary file 4 — Figure S4 [file ECE3-10-8235-s004.tif]

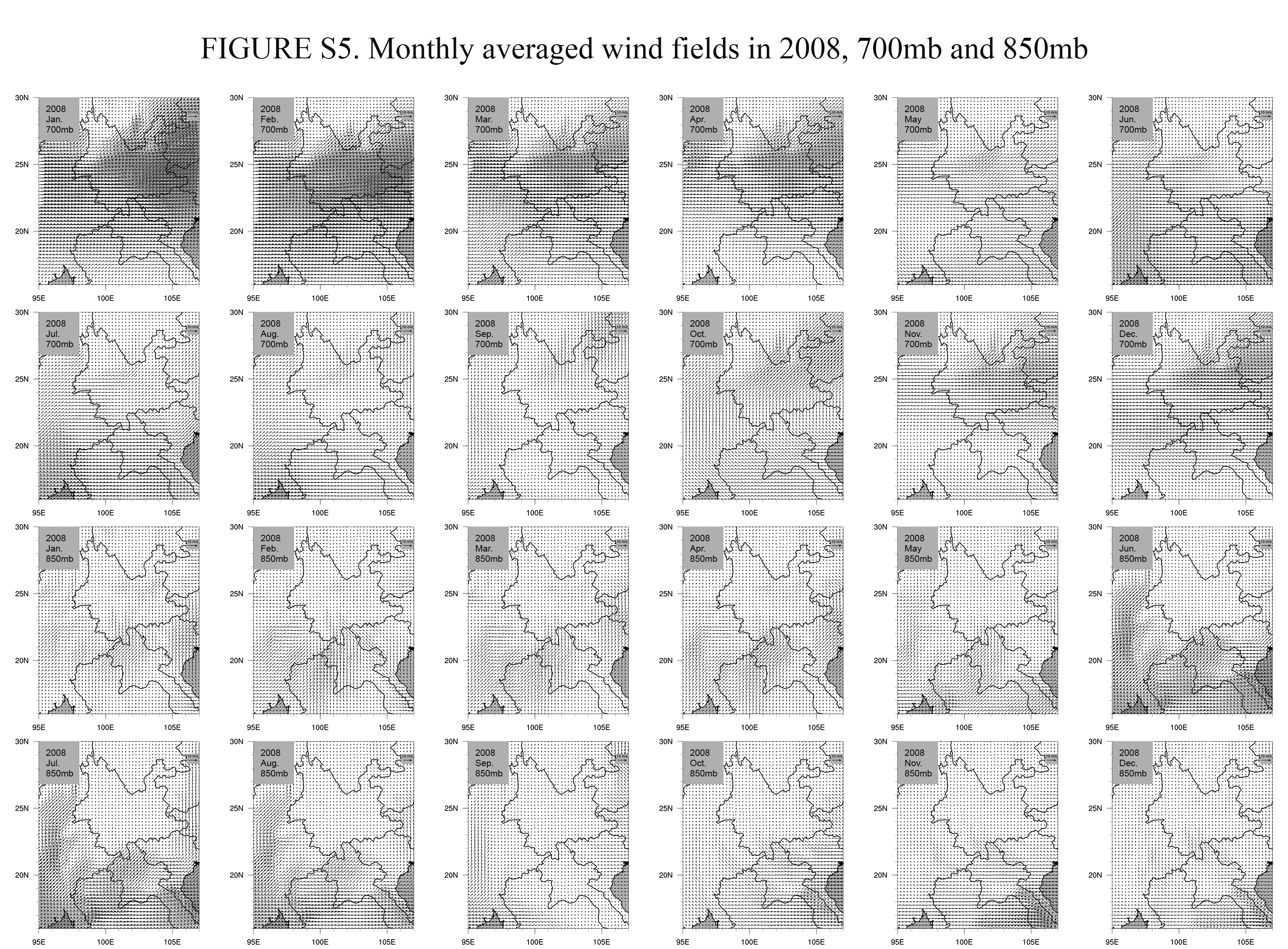

Supplement: Supplementary file 5 — Figure S5 [file ECE3-10-8235-s005.tif]

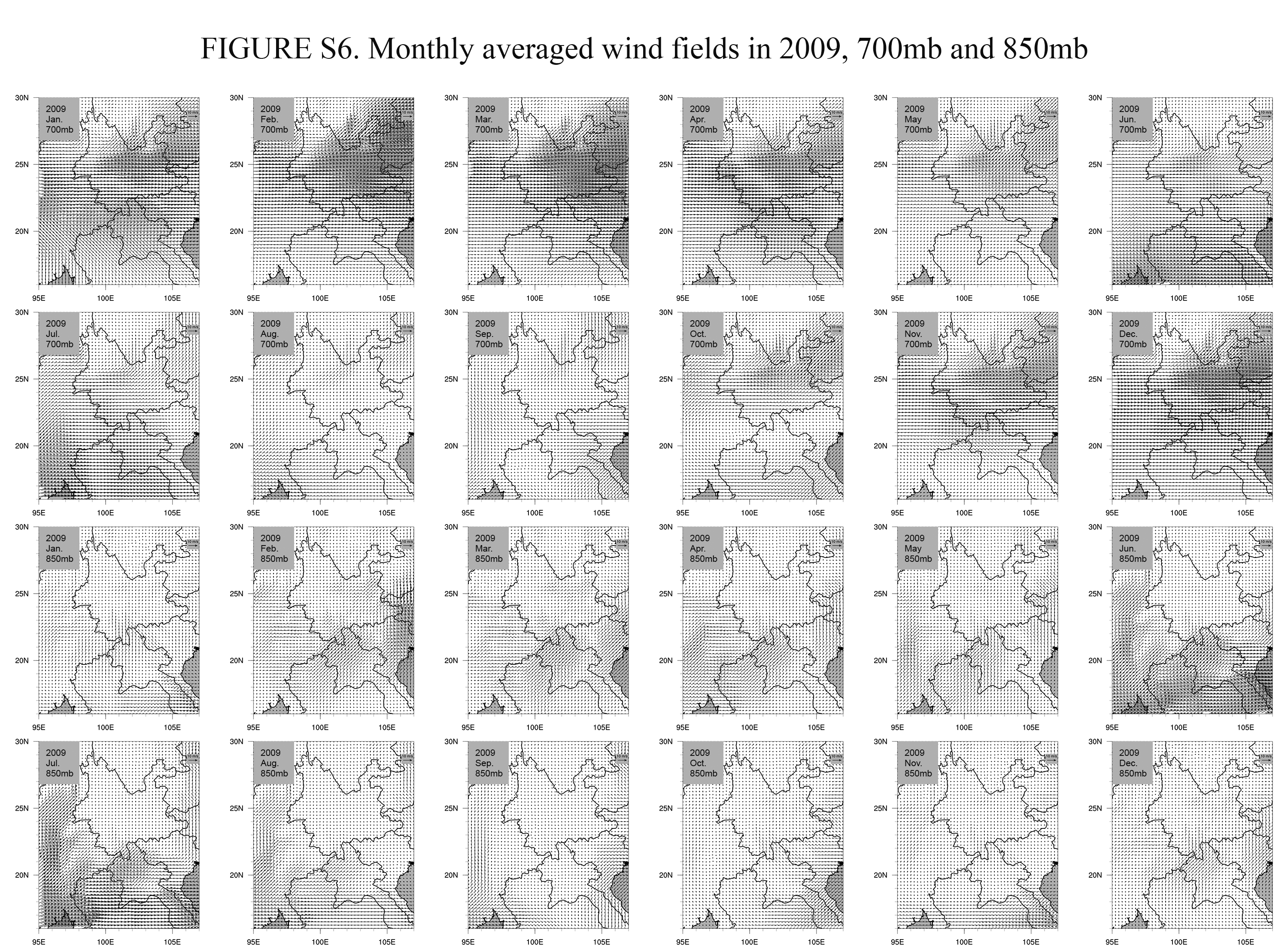

Supplement: Supplementary file 6 — Figure S6 [file ECE3-10-8235-s006.tif]

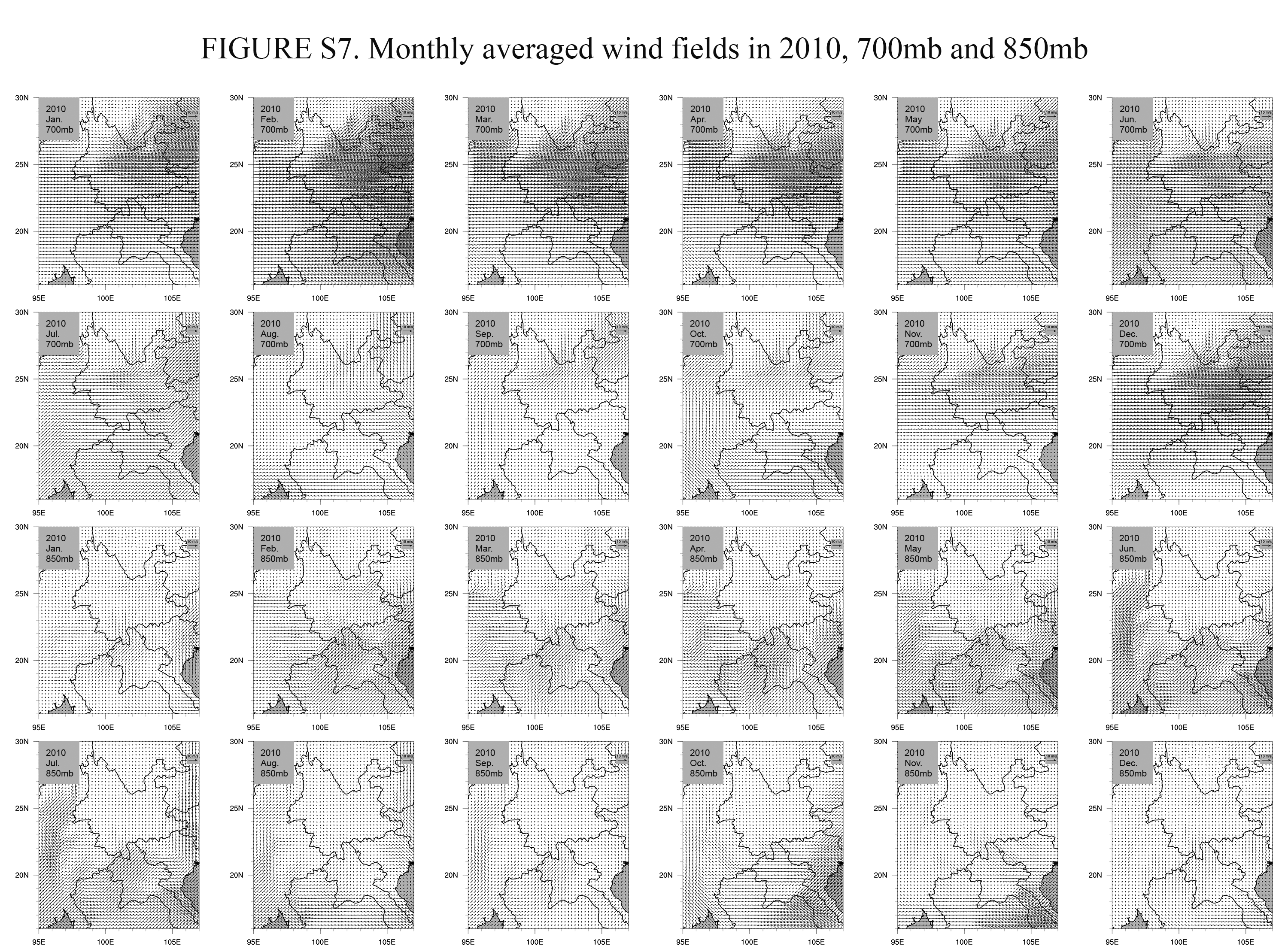

Supplement: Supplementary file 7 — Figure S7 [file ECE3-10-8235-s007.tif]

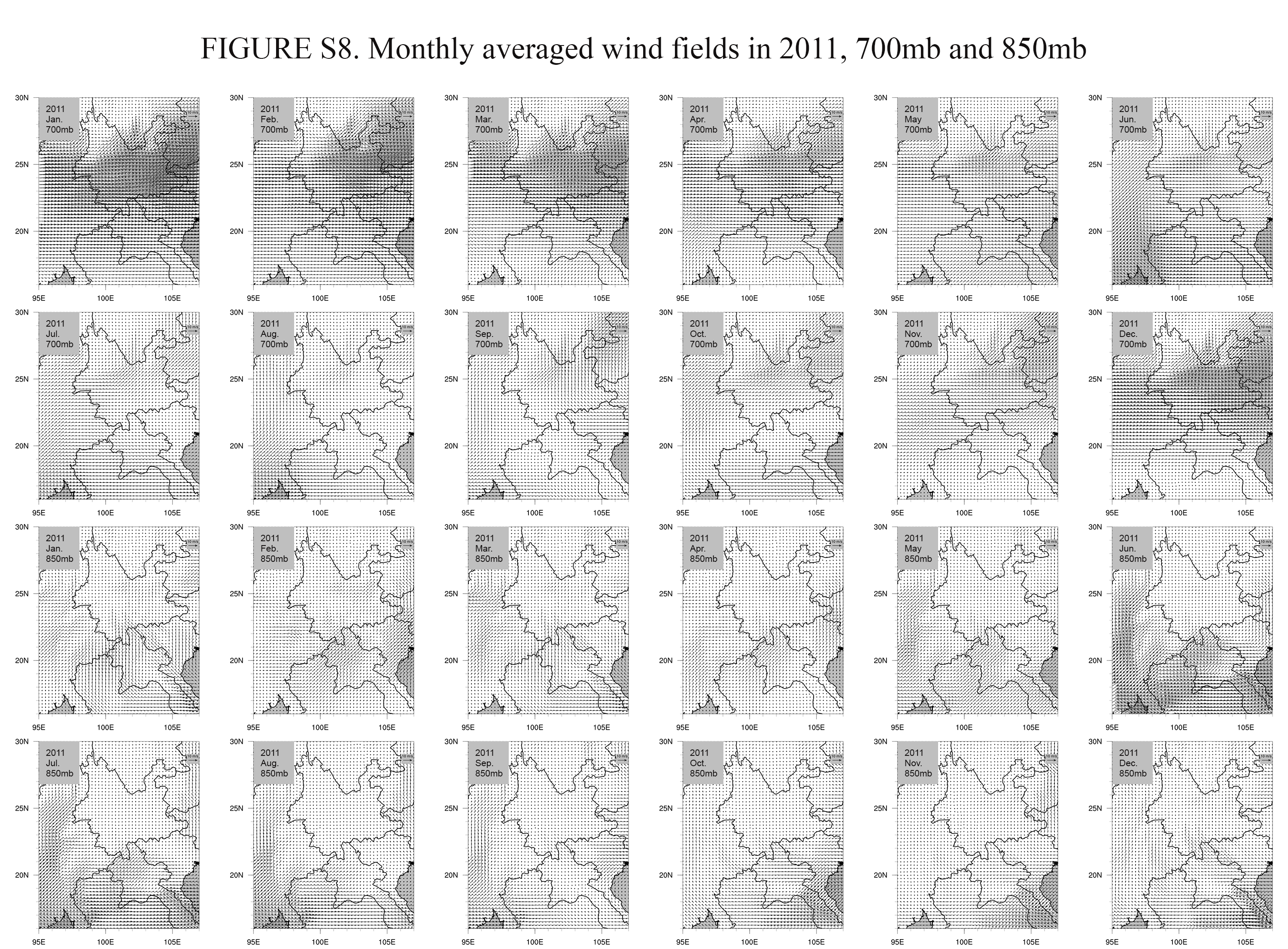

Supplement: Supplementary file 8 — Figure S8 [file ECE3-10-8235-s008.tif]

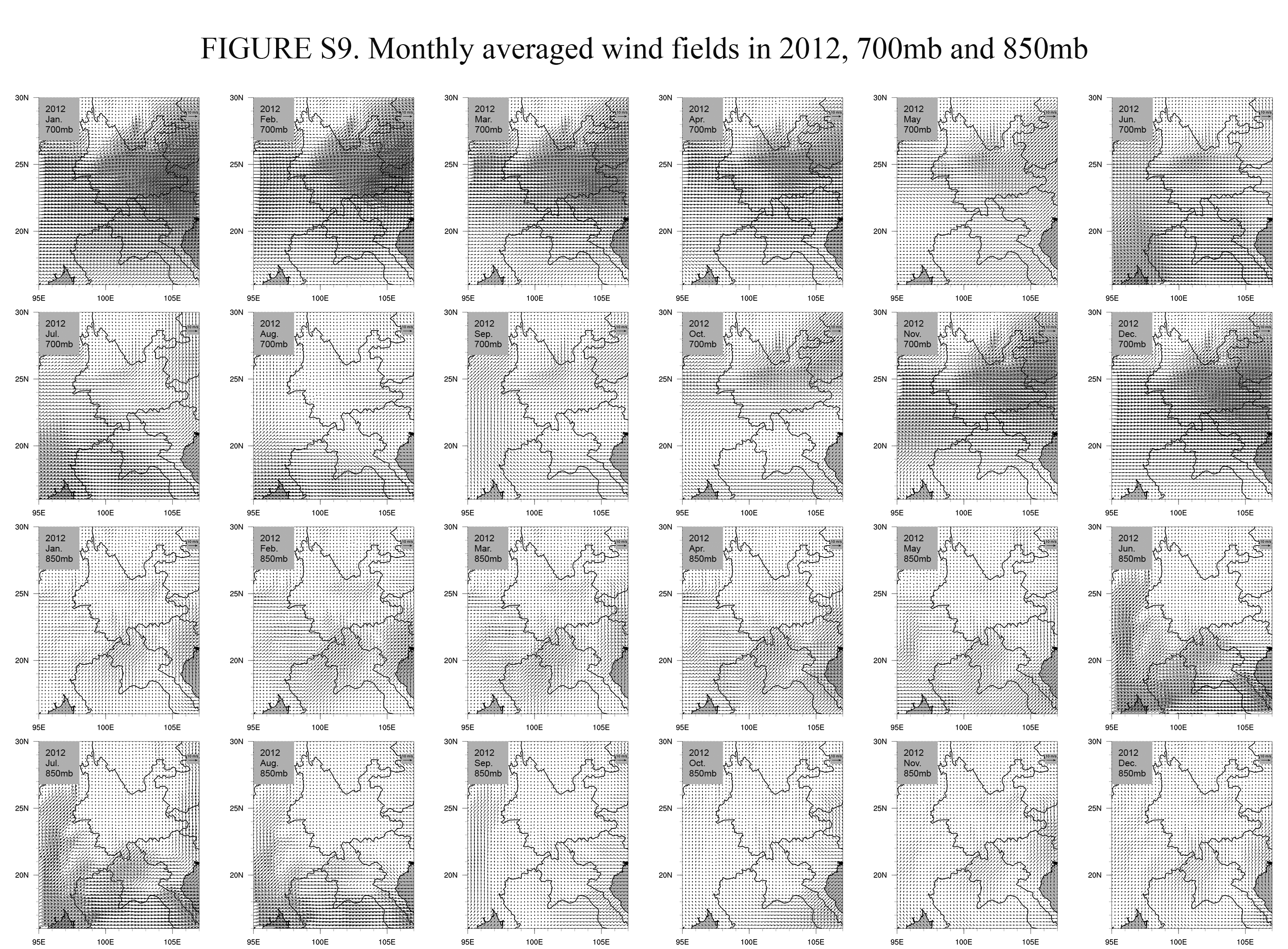

Supplement: Supplementary file 9 — Figure S9 [file ECE3-10-8235-s009.tif]

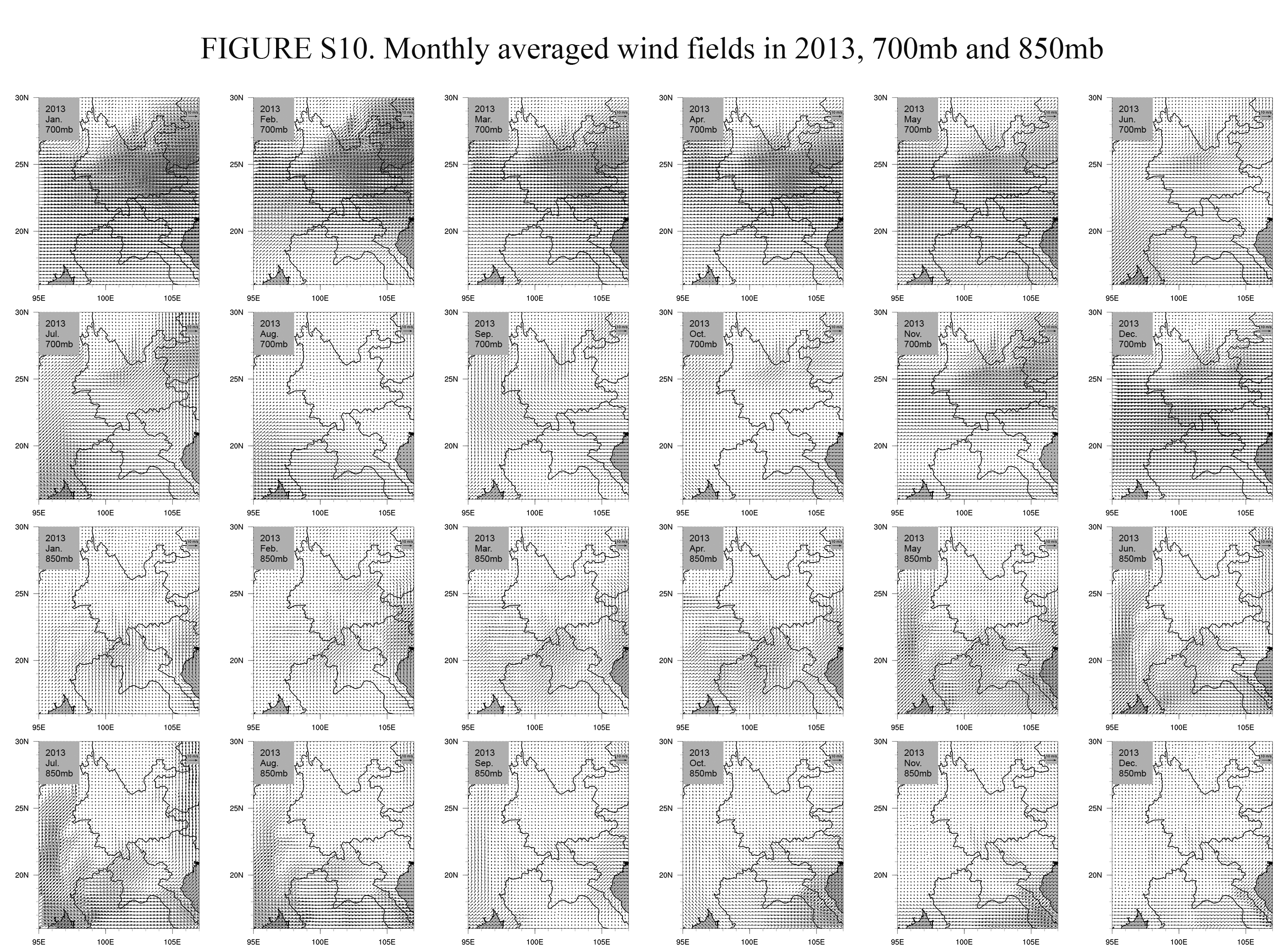

Supplement: Supplementary file 10 — Figure S10 [file ECE3-10-8235-s010.tif]
