# Supplementary material for: Migration sources and pathways of the pest species Sogatella furcifera in Yunnan, China, and across the border inferred from DNA and wind analyses
Source: Ecol Evol. 2020 Jul 17;10(15):8235–50. doi: 10.1002/ece3.6531 (PMC7417236; doi:10.1002/ece3.6531)
Supplement: Supplementary file 11 — Figure S11 [file ECE3-10-8235-s011.pdf]

Year	Baric /mb	Jun			Jul			Aug			Sep		
		10d	20d	30d	10d	20d	30d	10d	20d	30d	10d	20d	30d
2004	700	3	1/3**	10**	8**	7**	2**	3**	1*/6**	9**	6**	1/1**	1**
	850	4**	2*	2/1*	1/1*/3**	1**		3**	6**	5**	1*/4**	1/3**	2/1*
2005	700	6**	2/4**	1*/7**	1/1**	1/5**	5**	7**	6**	7**	10**	2**	1**
	850	1/1*/2**	2/3**	1*	1*	1*/2**	7**	1**	1/1*/3**	1*/3**	8**	6**	3**
2006	700	7**	7**	1/3**	8**	8**	1*/9**	1/1*/7**	1/4**	1*/4**	8**	1*/7**	4**
	850	2**	1/2*		2*	3*	6**	8**	5**	1**	3**	1*/4**	3**
2007	700	1/5**	4**	2*/5**	1/1*/3**	5**	5**	3*/6**	10**	1/1*/4**	2/1*/5**	3/4**	5**
	850	1/3*	2**	1*		1*/1**		4**	1/1*/5**	3*	2*/5**	1*/5**	6**
2008	700	7**	2/7**	3*/5**	2/6**	7**	6**	1*/7**	3**	1/4**	3**		7**
	850	2*/3**	4**	2*/4**	3**	2**	1*	1*/5**	1**	2**	2**		6**
2009	700	6**	4**	1/1*/6**	1*/6**	6**	1/8**	8**	3**	4**		6**	1/6**
	850	1*/3**	1*	1*/1**		1*/4**	1/1*	6**		2**		4**	8**
2010	700	3**	3**	3**	5**	2**	2*/5**	6**	2**	5**	9**	2**	3**
	850	2/3**	1/1**	1**		1*/2**	2**	4**		6**	1/1*/2**		3**
2011	700	2**	1**	9**	4**	7**	5**	7**		8**	3**	3**	2**
	850	2*		1/5**	1*/2**	2*/3**	2**	1*/3**		2*/5**	2*/1**		1/3**
2012	700	3**	5**	6**	3**	5**	10**	8**	1*/6**	7**	1*/3**	1/2*	6**
	850		2/2*/3**	1**	1*	2*	1*/6**	8**	3**	3**	1*/5**	3**	3**
2013	700	1/1*/3**	3**	6**		7**	3**	1*/3**	7**	9**	1/3**	1*/1**	8**
	850	1*/3**	1**	1**			3**	3**	2*/5**	2*/4**	3**	1*/2**	1*/9**

FIGURE S11 Schematic chart for northerly winds favouring southward (return) migration of *S. furcifera* from June to September in 2004–2013. Numbers and symbols indicate the duration (in days) and influencing range of the winds in these days in a 10 d period of each month, two asterisks (**) denote influencing range reached south Yunnan (areas in rows C and D in Figure 2), one asterisk (*) denotes influencing range reached central Yunnan (areas in row E), influencing range only reaching northeast Yunnan (areas in row F) were not denoted. Grey empty cells denote absence of such winds in the 10 d period.
