# Supplementary material for: Migration sources and pathways of the pest species Sogatella furcifera in Yunnan, China, and across the border inferred from DNA and wind analyses
Source: Ecol Evol. 2020 Jul 17;10(15):8235–50. doi: 10.1002/ece3.6531 (PMC7417236; doi:10.1002/ece3.6531)
Supplement: Supplementary file 12 — Figure S12 [file ECE3-10-8235-s012.pdf]

Year	Baric /mb	Jun			Jul			Aug			Sep		
		10d	20d	30d	10d	20d	30d	10d	20d	30d	10d	20d	30d
2004	700	1/2*/1**	1/4*/1**	1/1*	3/1*/1**	2/3*	3/1*/6**	2*/4**	2/3*/4**	4/2*/1**	1/3*/3**	1*/5**	1*/1**
	850	2/4*/1**	3/2*/2**	4	2	3	2/2*/4**	3/2*	5/3*/1**	7/2*	2/2*/3**	1*/6**	2/2*/1**
2005	700	4	1*/3**	2		1/1*/1**	2/1*/6**	6/1**	2*/5**	3*/2**	1/1*/8**	8**	2/1*/3**
	850	7/2*	4/1*	2		3/1*	2/5*/2**	7/1**	2/3*/2**	7/1*/2**	4/2*/4**	2*/7**	4/2*/3**
2006	700	2*	1*/2**	1	4/1*	4/2*/1**	2/6**	2*/8**	10**	1/4*/4**	2*/3**	1/2*/3**	1/7*
	850	4	3/2*	1	6	5/1*	4/3*/2**	4/2*/3**	4/4*/2**	4/4*/1**	1/3**	2/3*/4**	4/3*/3**
2007	700		2**	2	1/1*	2/1*		3*/6**	3/1*/5**	2/1*/5**	1/3*/2**	3*/5**	2*/6**
	850	5	4	3	3	3	1	3/4*/2**	4/1*/2**	1/3*/2**	4/2*/2**	8**	1/5*/3**
2008	700	1/3*/1**	2/2**	1/2*/1**	5**	2/2*/3**	2/3*/2**	1/2*/7**	2/2*/2**	3/1*/2**	1/1*/4**	5**	2/3*/4**
	850	5/1*/1**	4/2*	2/3*	2/2**	5/1*	5/3**	4/5*	2/2**	7/1*	1/3*/4**	5**	3/3*/2**
2009	700	1/2*/1**	3	3/2*	1/3*	2/2*/4**	1*	3*/6**	2*/2**	6**	1/1*/3**	1/4*/2**	1*/8**
	850	4/2*	3/2**	2/1*	3/1*	4/3*/1**	2/1*	5/3*	2/1*/1**	3*/3**	1/2**	1/6*/1**	2*/8**
2010	700	1/1*	1*	1*		3*	3*/3**	1/3*/5**	4**	6*/4**	2/2*/1**	2*/4**	3**
	850	5/3*	3/1*	1/2*		4/1*	4/2*	5/3*/1**	4*	4/1*/5**	6/2*	1/1*/4**	5/1*/3**
2011	700	1*		1*/4**	4*	2/3*/2**	1*/2**	2*/6**	1*/5**	1/2*/7**	1/1*/7**	1/2*/4**	1*/2**
	850	3/1**	2	6/2**	4	3/1*/2**	3/1*/2**	6/1*/2**	1/1*/3**	4/1*/5**	1/2*/4**	3/2*/3**	1/6*/3**
2012	700	1	3/3*	1**	1/1**	1*/2**	2/1*/7**	2/2*/6**	1*/6**	1/3*/3**	7**	1*/2**	1/3*
	850	3/2*/1**	8/2*	4	1/1*	3	5/5*	4/6*	3/5*/1**	5/2*/2**	2*/5**	4/1*/3**	4/2*/3**
2013	700	1/2*/1**	2**	2/3**		1/3**	1/4*/4**	8**	4/4**	1/1*/7**	1/1*/2**	1*/9**	2*/8**
	850	3/2*/1**	2/2**	2/1*/1**		5/1*	3/4*/2**	1/3*/3**	3/4*	3/4*/2**	3/1*/3**	9**	2/1*/7**

FIGURE S12 Schematic chart for easterly winds favouring westward migration of *S. furcifera* from June to September in 2004–2013. Numbers and symbols indicate the duration (days) and influencing range of the winds in these days in a 10 d period of each month, two asterisks (**) denote influencing range reached westernmost Yunnan (areas in column 2 and westward of Figure 2), one asterisk (*) denotes influencing range reached central-west Yunnan (areas in column 3), influencing range only reaching central Yunnan (areas in column 4) were not denoted. Grey empty cells denote absence of such winds in the 10 d period.
