# Supplementary material for: Migration sources and pathways of the pest species Sogatella furcifera in Yunnan, China, and across the border inferred from DNA and wind analyses
Source: Ecol Evol. 2020 Jul 17;10(15):8235–50. doi: 10.1002/ece3.6531 (PMC7417236; doi:10.1002/ece3.6531)
Supplement: Supplementary file 13 — Table S1 [file ECE3-10-8235-s013.pdf]

**TABLE S1** The number of haplotypes ($h$), haplotype diversity ($H$), nucleotide diversity ($\pi$), ratio of shared haplotypes ($R_{shr}$), and ratio of private haplotypes ($R_{prv}$) of each population and the global dataset.

| Population | $h$ | $H$ | $\pi$ | $R_{shr}$ | $R_{prv}$ |
|---|---|---|---|---|---|
| All | 205 | 0.8377 ± 0.0076 | 0.00108 ± 0.00069 | 0.17 | 0.83 |
| 1 | 11 | 0.8947 ± 0.0520 | 0.00092 ± 0.00064 | 0.91 | 0.09 |
| 2 | 8 | 0.7000 ± 0.1092 | 0.00070 ± 0.00053 | 0.75 | 0.25 |
| 3 | 7 | 0.7842 ± 0.0671 | 0.00070 ± 0.00052 | 0.71 | 0.29 |
| 4 | 14 | 0.9474 ± 0.0344 | 0.00115 ± 0.00076 | 0.57 | 0.43 |
| 5 | 9 | 0.8421 ± 0.0607 | 0.00081 ± 0.00058 | 0.67 | 0.33 |
| 6 | 6 | 0.7842 ± 0.0529 | 0.00073 ± 0.00054 | 0.67 | 0.33 |
| 7 | 12 | 0.9421 ± 0.0295 | 0.00093 ± 0.00065 | 0.67 | 0.33 |
| 8 | 11 | 0.9158 ± 0.0377 | 0.00101 ± 0.00069 | 0.55 | 0.45 |
| 9 | 10 | 0.8895 ± 0.0508 | 0.00081 ± 0.00058 | 0.70 | 0.30 |
| 10 | 5 | 0.6000 ± 0.1011 | 0.00061 ± 0.00048 | 1.00 | 0.00 |
| 11 | 10 | 0.8579 ± 0.0623 | 0.00070 ± 0.00053 | 0.60 | 0.40 |
| 12 | 9 | 0.7474 ± 0.0982 | 0.00066 ± 0.00050 | 1.00 | 0.00 |
| 13 | 10 | 0.8316 ± 0.0751 | 0.00057 ± 0.00045 | 0.60 | 0.40 |
| 14 | 4 | 0.5947 ± 0.0977 | 0.00047 ± 0.00040 | 1.00 | 0.00 |
| 15 | 8 | 0.8368 ± 0.0588 | 0.00075 ± 0.00055 | 0.75 | 0.25 |
| 16 | 8 | 0.8316 ± 0.0568 | 0.00047 ± 0.00040 | 1.00 | 0.00 |
| 17 | 9 | 0.7053 ± 0.1114 | 0.00087 ± 0.00062 | 0.56 | 0.44 |
| 18 | 11 | 0.8421 ± 0.0772 | 0.00081 ± 0.00058 | 0.73 | 0.27 |
| 19 | 13 | 0.9263 ± 0.0431 | 0.00093 ± 0.00064 | 0.69 | 0.31 |
| 20 | 9 | 0.8579 ± 0.0537 | 0.00097 ± 0.00067 | 0.56 | 0.44 |
| 21 | 7 | 0.7263 ± 0.0917 | 0.00074 ± 0.00055 | 0.57 | 0.43 |
| 22 | 13 | 0.9158 ± 0.0462 | 0.00084 ± 0.00060 | 0.69 | 0.31 |
| 23 | 11 | 0.8737 ± 0.0642 | 0.00101 ± 0.00069 | 0.64 | 0.36 |
| 24 | 8 | 0.6947 ± 0.1076 | 0.00095 ± 0.00066 | 0.75 | 0.25 |
| 25 | 9 | 0.8474 ± 0.0613 | 0.00073 ± 0.00054 | 0.78 | 0.22 |
| 26 | 11 | 0.9105 ± 0.0419 | 0.00066 ± 0.00050 | 0.91 | 0.09 |
| 27 | 9 | 0.8263 ± 0.0734 | 0.00055 ± 0.00044 | 0.89 | 0.11 |
| 28 | 7 | 0.6789 ± 0.1024 | 0.00068 ± 0.00052 | 0.57 | 0.43 |
| 29 | 7 | 0.7263 ± 0.0917 | 0.00073 ± 0.00054 | 0.71 | 0.29 |
| 30 | 14 | 0.9526 ± 0.0326 | 0.00121 ± 0.00079 | 0.57 | 0.43 |
| 31 | 7 | 0.8211 ± 0.0561 | 0.00077 ± 0.00056 | 0.86 | 0.14 |
| 32 | 10 | 0.8368 ± 0.0757 | 0.00065 ± 0.00050 | 0.80 | 0.20 |
| 33 | 7 | 0.8158 ± 0.0496 | 0.00081 ± 0.00058 | 0.71 | 0.29 |
| 34 | 5 | 0.6263 ± 0.1103 | 0.00065 ± 0.00050 | 0.80 | 0.20 |
| 35 | 12 | 0.8474 ± 0.0786 | 0.00064 ± 0.00049 | 0.67 | 0.33 |
| 36 | 11 | 0.8579 ± 0.0645 | 0.00077 ± 0.00056 | 0.64 | 0.36 |
| 37 | 10 | 0.8526 ± 0.0630 | 0.00077 ± 0.00056 | 0.60 | 0.40 |
| 38 | 13 | 0.9316 ± 0.0386 | 0.00093 ± 0.00065 | 0.69 | 0.31 |
| 39 | 8 | 0.7000 ± 0.1092 | 0.00069 ± 0.00052 | 0.63 | 0.38 |

| | | | | | |
|---|---|---|---|---|---|
| 40 | 8 | 0.8211 ± 0.0598 | 0.00072 ± 0.00054 | 0.75 | 0.25 |
| 41 | 13 | 0.9474 ± 0.0323 | 0.00061 ± 0.00048 | 0.85 | 0.15 |
| 42 | 11 | 0.9105 ± 0.0419 | 0.00082 ± 0.00059 | 0.64 | 0.36 |
| 43 | 8 | 0.7421 ± 0.0961 | 0.00060 ± 0.00047 | 0.50 | 0.50 |
| 44 | 9 | 0.7895 ± 0.0859 | 0.00082 ± 0.00059 | 0.33 | 0.67 |
| 45 | 12 | 0.8478 ± 0.0786 | 0.00105 ± 0.00071 | 0.33 | 0.67 |
| 46 | 10 | 0.8738 ± 0.0520 | 0.00083 ± 0.00059 | 0.90 | 0.10 |
| 47 | 6 | 0.8105 ± 0.0467 | 0.00072 ± 0.00054 | 0.67 | 0.33 |
| 48 | 7 | 0.8105 ± 0.0525 | 0.00075 ± 0.00055 | 0.57 | 0.43 |
| 49 | 8 | 0.8684 ± 0.0444 | 0.00070 ± 0.00052 | 0.75 | 0.25 |
| 50 | 12 | 0.9000 ± 0.0532 | 0.00102 ± 0.00069 | 0.58 | 0.42 |
| 51 | 9 | 0.8895 ± 0.0416 | 0.00060 ± 0.00047 | 0.89 | 0.11 |
| 52 | 7 | 0.8158 ± 0.0496 | 0.00072 ± 0.00053 | 0.71 | 0.29 |
| 53 | 9 | 0.8526 ± 0.0532 | 0.00075 ± 0.00055 | 0.67 | 0.33 |
| 54 | 7 | 0.8368 ± 0.0482 | 0.00065 ± 0.00050 | 0.71 | 0.29 |
| 55 | 8 | 0.8474 ± 0.0512 | 0.00084 ± 0.00060 | 0.63 | 0.38 |
| 56 | 11 | 0.8421 ± 0.0772 | 0.00085 ± 0.00060 | 0.45 | 0.55 |
| 57 | 8 | 0.7737 ± 0.0825 | 0.00072 ± 0.00053 | 0.88 | 0.13 |
| 58 | 11 | 0.9105 ± 0.0419 | 0.00118 ± 0.00077 | 0.55 | 0.45 |
| 59 | 7 | 0.7632 ± 0.0790 | 0.00076 ± 0.00055 | 0.86 | 0.14 |
| 60 | 9 | 0.8316 ± 0.0627 | 0.00079 ± 0.00057 | 0.67 | 0.33 |
