# Supplementary material for: Migration sources and pathways of the pest species Sogatella furcifera in Yunnan, China, and across the border inferred from DNA and wind analyses
Source: Ecol Evol. 2020 Jul 17;10(15):8235–50. doi: 10.1002/ece3.6531 (PMC7417236; doi:10.1002/ece3.6531)
Supplement: Supplementary file 14 — Table S2 [file ECE3-10-8235-s014.pdf]

TABLE S2 The K2P distances between populations (above diagonal) and the Nm values between populations (below diagonal), ∞ denotes infinite values.

Pop.	1	2	3	4	5	6	7	8	9	10	11	12	13	14	15	16	17	18	19	20	21	22	23	24	25	26	27	28	29	30	31	32	33	34	35	36	37	38	39	40	41	42	43	44	45	46	47	48	49	50	51	52	53	54	55	56	57	58	59	60
**1**		0.00062	0.00060	0.00088	0.00062	0.00060	0.00067	0.00075	0.00062	0.00054	0.00059	0.00059	0.00063	0.00050	0.00062	0.00051	0.00066	0.00063	0.00065	0.00070	0.00056	0.00061	0.00080	0.00074	0.00059	0.00053	0.00050	0.00056	0.00060	0.00082	0.00063	0.00059	0.00063	0.00054	0.00065	0.00064	0.00063	0.00069	0.00063	0.00056	0.00057	0.00065	0.00064	0.00065	0.00076	0.00061	0.00056	0.00058	0.00054	0.00079	0.00051	0.00059	0.00060	0.00054	0.00066	0.00073	0.00055	0.00081	0.00057	0.00061
**2**	∞		0.00052	0.00082	0.00052	0.00052	0.00060	0.00069	0.00054	0.00046	0.00050	0.00051	0.00053	0.00042	0.00052	0.00042	0.00059	0.00055	0.00059	0.00063	0.00046	0.00053	0.00072	0.00066	0.00051	0.00045	0.00041	0.00047	0.00052	0.00076	0.00055	0.00051	0.00056	0.00045	0.00056	0.00055	0.00055	0.00061	0.00054	0.00048	0.00050	0.00060	0.00055	0.00054	0.00067	0.00053	0.00048	0.00050	0.00046	0.00071	0.00042	0.00051	0.00053	0.00046	0.00058	0.00062	0.00047	0.00074	0.00049	0.00054
**3**	∞	∞		0.00078	0.00050	0.00049	0.00057	0.00066	0.00051	0.00043	0.00047	0.00048	0.00050	0.00039	0.00049	0.00039	0.00056	0.00052	0.00056	0.00060	0.00044	0.00050	0.00069	0.00063	0.00047	0.00042	0.00038	0.00044	0.00049	0.00073	0.00052	0.00048	0.00053	0.00042	0.00053	0.00052	0.00052	0.00059	0.00051	0.00045	0.00047	0.00057	0.00052	0.00051	0.00064	0.00050	0.00045	0.00047	0.00043	0.00068	0.00039	0.00048	0.00050	0.00043	0.00055	0.00060	0.00044	0.00071	0.00046	0.00051
**4**	∞	∞	∞		0.00081	0.00079	0.00086	0.00094	0.00080	0.00073	0.00079	0.00078	0.00084	0.00070	0.00080	0.00072	0.00085	0.00081	0.00083	0.00089	0.00076	0.00079	0.00099	0.00093	0.00079	0.00071	0.00070	0.00076	0.00079	0.00100	0.00082	0.00078	0.00082	0.00073	0.00084	0.00084	0.00082	0.00088	0.00083	0.00074	0.00076	0.00083	0.00084	0.00084	0.00096	0.00080	0.00074	0.00076	0.00074	0.00098	0.00070	0.00077	0.00079	0.00074	0.00085	0.00094	0.00074	0.00100	0.00077	0.00079
**5**	∞	∞	∞	62.389		0.00050	0.00059	0.00068	0.00053	0.00044	0.00046	0.00050	0.00048	0.00039	0.00048	0.00038	0.00057	0.00052	0.00058	0.00062	0.00043	0.00051	0.00071	0.00064	0.00048	0.00044	0.00037	0.00043	0.00050	0.00076	0.00053	0.00050	0.00054	0.00042	0.00054	0.00052	0.00053	0.00060	0.00050	0.00047	0.00048	0.00060	0.00052	0.00050	0.00064	0.00051	0.00047	0.00048	0.00044	0.00068	0.00039	0.00050	0.00051	0.00044	0.00056	0.00058	0.00045	0.00072	0.00047	0.00053
**6**	∞	∞	∞	∞	210.742		0.00057	0.00066	0.00051	0.00043	0.00047	0.00048	0.00050	0.00039	0.00049	0.00039	0.00056	0.00052	0.00056	0.00060	0.00044	0.00050	0.00069	0.00063	0.00048	0.00042	0.00038	0.00044	0.00049	0.00073	0.00052	0.00048	0.00053	0.00042	0.00053	0.00052	0.00052	0.00059	0.00051	0.00045	0.00047	0.00057	0.00052	0.00051	0.00064	0.00050	0.00045	0.00047	0.00043	0.00068	0.00039	0.00048	0.00050	0.00043	0.00055	0.00060	0.00044	0.00071	0.00046	0.00051
**7**	∞	∞	∞	∞	∞	∞		0.00073	0.00059	0.00051	0.00056	0.00057	0.00061	0.00048	0.00059	0.00049	0.00064	0.00060	0.00062	0.00067	0.00053	0.00058	0.00077	0.00072	0.00057	0.00050	0.00047	0.00053	0.00057	0.00080	0.00060	0.00057	0.00061	0.00051	0.00062	0.00062	0.00060	0.00067	0.00061	0.00053	0.00054	0.00063	0.00062	0.00062	0.00074	0.00058	0.00053	0.00055	0.00051	0.00077	0.00048	0.00056	0.00058	0.00051	0.00063	0.00072	0.00052	0.00079	0.00054	0.00058
**8**	3.463	4.478	6.688	3.956	13.512	3.874	5.561		0.00068	0.00060	0.00065	0.00065	0.00069	0.00056	0.00068	0.00057	0.00072	0.00069	0.00071	0.00076	0.00062	0.00067	0.00086	0.00080	0.00065	0.00059	0.00056	0.00062	0.00066	0.00088	0.00069	0.00065	0.00069	0.00060	0.00071	0.00071	0.00069	0.00075	0.00069	0.00062	0.00063	0.00071	0.00070	0.00071	0.00083	0.00067	0.00062	0.00064	0.00060	0.00085	0.00057	0.00065	0.00066	0.00060	0.00072	0.00080	0.00061	0.00087	0.00063	0.00067
**9**	∞	∞	∞	∞	∞	∞	∞	5.424		0.00045	0.00050	0.00051	0.00055	0.00042	0.00053	0.00044	0.00058	0.00054	0.00056	0.00062	0.00047	0.00052	0.00072	0.00066	0.00051	0.00044	0.00041	0.00047	0.00051	0.00074	0.00054	0.00051	0.00055	0.00045	0.00057	0.00056	0.00054	0.00061	0.00056	0.00047	0.00049	0.00057	0.00056	0.00056	0.00068	0.00052	0.00047	0.00049	0.00045	0.00072	0.00042	0.00050	0.00052	0.00045	0.00057	0.00066	0.00046	0.00073	0.00048	0.00053
**10**	∞	∞	∞	∞	∞	∞	∞	4.708	∞		0.00041	0.00042	0.00044	0.00033	0.00043	0.00033	0.00050	0.00046	0.00050	0.00054	0.00038	0.00044	0.00063	0.00057	0.00042	0.00037	0.00032	0.00038	0.00043	0.00067	0.00046	0.00042	0.00047	0.00036	0.00047	0.00046	0.00046	0.00053	0.00045	0.00040	0.00041	0.00051	0.00046	0.00045	0.00058	0.00044	0.00040	0.00041	0.00037	0.00062	0.00033	0.00042	0.00044	0.00037	0.00049	0.00054	0.00038	0.00065	0.00040	0.00045
**11**	∞	∞	∞	∞	∞	∞	∞	4.430	∞	∞		0.00047	0.00045	0.00036	0.00046	0.00035	0.00054	0.00050	0.00055	0.00059	0.00040	0.00048	0.00068	0.00062	0.00045	0.00041	0.00034	0.00040	0.00047	0.00073	0.00050	0.00047	0.00051	0.00039	0.00051	0.00049	0.00050	0.00057	0.00047	0.00044	0.00045	0.00057	0.00049	0.00047	0.00061	0.00048	0.00044	0.00045	0.00041	0.00066	0.00036	0.00047	0.00048	0.00041	0.00053	0.00056	0.00042	0.00069	0.00044	0.00050
**12**	∞	∞	∞	∞	∞	∞	∞	6.216	∞	∞	∞		0.00050	0.00038	0.00049	0.00038	0.00056	0.00051	0.00056	0.00060	0.00044	0.00050	0.00068	0.00063	0.00047	0.00042	0.00038	0.00044	0.00048	0.00072	0.00051	0.00048	0.00053	0.00042	0.00053	0.00052	0.00051	0.00058	0.00050	0.00045	0.00046	0.00056	0.00051	0.00051	0.00063	0.00050	0.00045	0.00047	0.00043	0.00067	0.00039	0.00048	0.00050	0.00043	0.00054	0.00057	0.00044	0.00071	0.00046	0.00051
**13**	36.171	∞	∞	1282.111	∞	848.501	∞	10.144	∞	∞	∞	∞		0.00039	0.00048	0.00037	0.00058	0.00053	0.00060	0.00063	0.00042	0.00053	0.00070	0.00064	0.00048	0.00046	0.00037	0.00042	0.00050	0.00077	0.00053	0.00049	0.00055	0.00042	0.00053	0.00051	0.00053	0.00061	0.00048	0.00049	0.00049	0.00063	0.00051	0.00049	0.00063	0.00052	0.00049	0.00050	0.00044	0.00068	0.00039	0.00052	0.00052	0.00044	0.00056	0.00056	0.00047	0.00073	0.00047	0.00055
**14**	2.715	3.751	7.401	5.411	7.309	4.272	13.985	10.298	6.366	3.916	7.159	12.386	32.223		0.00039	0.00028	0.00046	0.00042	0.00046	0.00051	0.00033	0.00040	0.00059	0.00053	0.00037	0.00033	0.00027	0.00033	0.00039	0.00064	0.00042	0.00038	0.00043	0.00031	0.00043	0.00042	0.00042	0.00049	0.00040	0.00036	0.00037	0.00048	0.00041	0.00040	0.00054	0.00040	0.00036	0.00037	0.00033	0.00057	0.00028	0.00039	0.00040	0.00033	0.00045	0.00049	0.00034	0.00061	0.00036	0.00042
**15**	339.794	∞	∞	∞	∞	∞	∞	6.588	∞	∞	∞	∞	∞	15.144		0.00038	0.00057	0.00052	0.00057	0.00062	0.00043	0.00051	0.00070	0.00064	0.00048	0.00044	0.00037	0.00043	0.00049	0.00075	0.00052	0.00049	0.00054	0.00042	0.00053	0.00052	0.00052	0.00060	0.00050	0.00047	0.00048	0.00059	0.00051	0.00050	0.00064	0.00051	0.00047	0.00048	0.00044	0.00068	0.00039	0.00050	0.00051	0.00044	0.00055	0.00057	0.00045	0.00072	0.00047	0.00053
**16**	4.891	7.490	25.321	16.766	11.718	10.267	∞	5.916	15.182	7.573	50.420	∞	∞	∞	∞		0.00047	0.00042	0.00049	0.00052	0.00032	0.00042	0.00059	0.00053	0.00037	0.00035	0.00026	0.00032	0.00039	0.00066	0.00042	0.00038	0.00044	0.00031	0.00042	0.00041	0.00042	0.00050	0.00038	0.00038	0.00037	0.00050	0.00040	0.00039	0.00053	0.00042	0.00038	0.00039	0.00034	0.00057	0.00028	0.00041	0.00041	0.00034	0.00045	0.00046	0.00036	0.00062	0.00037	0.00043
**17**	∞	∞	∞	23.775	∞	∞	31.874	4.880	∞	∞	36.079	33.436	21.195	2.668	19.618	4.023		0.00059	0.00062	0.00066	0.00051	0.00057	0.00076	0.00070	0.00055	0.00049	0.00045	0.00051	0.00056	0.00079	0.00058	0.00055	0.00060	0.00049	0.00061	0.00060	0.00059	0.00066	0.00059	0.00052	0.00054	0.00063	0.00060	0.00060	0.00072	0.00056	0.00052	0.00054	0.00050	0.00075	0.00046	0.00055	0.00057	0.00050	0.00062	0.00069	0.00051	0.00078	0.00053	0.00058
**18**	6.822	12.819	∞	17.689	108.202	13.140	∞	11.214	27.408	12.406	462.093	∞	∞	∞	∞	∞	6.133		0.00058	0.00063	0.00047	0.00053	0.00072	0.00066	0.00050	0.00045	0.00041	0.00047	0.00052	0.00076	0.00055	0.00051	0.00056	0.00045	0.00056	0.00055	0.00055	0.00062	0.00054	0.00048	0.00050	0.00059	0.00055	0.00054	0.00067	0.00053	0.00048	0.00050	0.00046	0.00071	0.00042	0.00051	0.00053	0.00046	0.00058	0.00063	0.00047	0.00074	0.00049	0.00054
**19**	5.361	8.369	28.858	6.730	248.710	6.523	22.761	73.897	13.150	8.691	10.601	30.871	∞	82.980	16.518	27.187	6.716	∞		0.00065	0.00052	0.00056	0.00077	0.00071	0.00055	0.00047	0.00046	0.00052	0.00056	0.00077	0.00059	0.00056	0.00059	0.00049	0.00061	0.00061	0.00059	0.00065	0.00061	0.00050	0.00053	0.00060	0.00061	0.00060	0.00073	0.00056	0.00050	0.00053	0.00050	0.00076	0.00046	0.00053	0.00056	0.00050	0.00062	0.00072	0.00050	0.00077	0.00053	0.00056
**20**	∞	∞	∞	∞	∞	∞	∞	3.697	∞	∞	∞	∞	∞	4.772	∞	15.612	109.907	23.197	7.264		0.00056	0.00061	0.00080	0.00074	0.00060	0.00053	0.00050	0.00056	0.00060	0.00082	0.00063	0.00060	0.00064	0.00054	0.00065	0.00065	0.00063	0.00069	0.00064	0.00056	0.00057	0.00066	0.00064	0.00065	0.00077	0.00060	0.00056	0.00058	0.00054	0.00080	0.00051	0.00058	0.00061	0.00054	0.00066	0.00075	0.00055	0.00081	0.00057	0.00061
**21**	∞	∞	∞	115.381	∞	∞	∞	12.125	∞	∞	∞	∞	∞	12.024	∞	40.412	13306.105	∞	∞	∞		0.00045	0.00065	0.00058	0.00042	0.00038	0.00031	0.00037	0.00044	0.00070	0.00046	0.00044	0.00048	0.00036	0.00048	0.00046	0.00047	0.00054	0.00044	0.00041	0.00042	0.00054	0.00046	0.00044	0.00058	0.00045	0.00041	0.00042	0.00038	0.00063	0.00033	0.00044	0.00045	0.00038	0.00050	0.00053	0.00040	0.00066	0.00041	0.00047
**22**	5.788	9.743	42.465	11.997	30.908	9.606	∞	12.452	15.792	9.086	29.469	∞	∞	∞	∞	∞	5.403	∞	∞	13.350	∞		0.00071	0.00065	0.00049	0.00043	0.00040	0.00045	0.00050	0.00074	0.00053	0.00050	0.00054	0.00043	0.00055	0.00054	0.00053	0.00060	0.00054	0.00046	0.00048	0.00057	0.00054	0.00053	0.00066	0.00051	0.00046	0.00048	0.00044	0.00070	0.00040	0.00049	0.00051	0.00044	0.00056	0.00063	0.00045	0.00072	0.00047	0.00052
**23**	7.444	13.880	∞	18.725	112.322	13.996	∞	11.558	28.842	13.344	432.621	∞	∞	∞	∞	∞	6.666	∞	∞	25.051	∞	∞		0.00083	0.00068	0.00063	0.00059	0.00065	0.00068	0.00092	0.00072	0.00068	0.00073	0.00063	0.00073	0.00073	0.00072	0.00079	0.00070	0.00066	0.00066	0.00077	0.00072	0.00072	0.00084	0.00070	0.00066	0.00068	0.00064	0.00087	0.00060	0.00069	0.00070	0.00064	0.00075	0.00080	0.00065	0.00091	0.00067	0.00072
**24**	154.576	∞	∞	13.041	∞	22.627	31.891	12.250	90.008	∞	31.265	∞	∞	4.656	30.457	6.551	∞	23.212	350.313	25.060	∞	13.216	25.620		0.00062	0.00057	0.00053	0.00059	0.00063	0.00086	0.00066	0.00063	0.00068	0.00057	0.00068	0.00067	0.00066	0.00073	0.00065	0.00060	0.00061	0.00071	0.00066	0.00066	0.00078	0.00064	0.00060	0.00062	0.00058	0.00082	0.00054	0.00063	0.00065	0.00058	0.00069	0.00075	0.00059	0.00085	0.00061	0.00066
**25**	∞	∞	∞	∞	∞	∞	∞	7.092	∞	∞	∞	∞	∞	7.420	∞	25.337	∞	∞	29.332	∞	∞	42.860	∞	∞		0.00042	0.00036	0.00042	0.00048	0.00073	0.00051	0.00047	0.00052	0.00040	0.00052	0.00051	0.00051	0.00058	0.00049	0.00045	0.00046	0.00057	0.00050	0.00049	0.00063	0.00049	0.00045	0.00046	0.00042	0.00066	0.00037	0.00048	0.00049	0.00042	0.00054	0.00058	0.00043	0.00070	0.00045	0.00051
**26**	8.089	14.830	∞	29.795	41.147	18.149	∞	8.585	35.432	14.197	∞	∞	∞	∞	∞	∞	6.517	∞	∞	43.004	∞	∞	∞	15.685	∞		0.00032	0.00038	0.00042	0.00065	0.00045	0.00042	0.00046	0.00036	0.00048	0.00047	0.00045	0.00052	0.00047	0.00038	0.00040	0.00048	0.00047	0.00047	0.00059	0.00043	0.00038	0.00040	0.00037	0.00063	0.00033	0.00041	0.00043	0.00037	0.00048	0.00057	0.00037	0.00064	0.00040	0.00044
**27**	∞	∞	∞	∞	∞	∞	∞	6.220	∞	∞	∞	∞	∞	7.979	∞	41.712	1076.964	∞	17.363	∞	∞	41.599	∞	∞	∞	∞		0.00031	0.00038	0.00064	0.00041	0.00038	0.00042	0.00030	0.00042	0.00040	0.00041	0.00048	0.00038	0.00035	0.00037	0.00048	0.00040	0.00038	0.00052	0.00040	0.00035	0.00037	0.00032	0.00057	0.00027	0.00038	0.00040	0.00032	0.00044	0.00047	0.00034	0.00060	0.00035	0.00041
**28**	2.879	4.214	10.039	5.543	10.495	4.526	20.867	13.003	7.062	4.416	7.912	18.023	∞	∞	14.835	∞	2.979	∞	∞	5.020	25.918	∞	∞	6.549	10.089	∞	9.927		0.00044	0.00070	0.00047	0.00044	0.00048	0.00036	0.00048	0.00046	0.00047	0.00054	0.00044	0.00041	0.00042	0.00054	0.00046	0.00044	0.00058	0.00045	0.00041	0.00043	0.00038	0.00063	0.00033	0.00044	0.00045	0.00038	0.00050	0.00053	0.00040	0.00066	0.00041	0.00047
**29**	∞	195.923	10.759	11.436	11.148	32.101	6.756	2.128	18.203	76.631	8.506	6.259	4.776	1.441	6.336	2.114	∞	2.451	2.460	13.112	6.870	2.467	2.680	7.281	10.727	2.878	10.554	1.433		0.00073	0.00052	0.00048	0.00053	0.00042	0.00053	0.00052	0.00052	0.00059	0.00051	0.00045	0.00047	0.00057	0.00052	0.00051	0.00064	0.00050	0.00045	0.00047	0.00043	0.00068	0.00039	0.00048	0.00050	0.00043	0.00055	0.00060	0.00044	0.00071	0.00046	0.00051
**30**	13.737	40.604	∞	96.267	∞	31.930	∞	12.762	186.331	32.614	∞	∞	∞	∞	∞	∞	11.090	∞	∞	636.892	∞	∞	∞	337.912	∞	∞	∞	∞	3.653		0.00075	0.00072	0.00076	0.00067	0.00078	0.00078	0.00076	0.00081	0.00077	0.00068	0.00070	0.00077	0.00078	0.00079	0.00090	0.00073	0.00068	0.00071	0.00068	0.00092	0.00064	0.00071	0.00073	0.00068	0.00079	0.00088	0.00068	0.00094	0.00071	0.00074
**31**	∞	∞	∞	∞	635.767	∞	∞	3.144	∞	∞	∞	∞	∞	3.446	∞	8.039	∞	12.691	5.550	∞	∞	8.956	13.045	15.673	∞	14.802	∞	3.641	29.768	23.283		0.00051	0.00056	0.00045	0.00056	0.00055	0.00055	0.00062	0.00054	0.00048	0.00050	0.00060	0.00055	0.00054	0.00067	0.00052	0.00048	0.00050	0.00046	0.00071	0.00042	0.00051	0.00053	0.00046	0.00058	0.00063	0.00047	0.00074	0.00049	0.00054
**32**	∞	∞	∞	∞	∞	∞	∞	5.483	∞	∞	∞	∞	∞	7.497	∞	31.640	109.218	∞	20.136	∞	∞	71.391	∞	1859.677	∞	∞	∞	9.078	9.599	∞	∞		0.00053	0.00042	0.00053	0.00052	0.00051	0.00058	0.00050	0.00045	0.00046	0.00056	0.00051	0.00051	0.00064	0.00050	0.00045	0.00047	0.00043	0.00067	0.00039	0.00048	0.00050	0.00043	0.00054	0.00060	0.00044	0.00071	0.00046	0.00051
**33**	∞	∞	∞	∞	∞	∞	∞	4.763	∞	∞	∞	∞	∞	4.442	∞	9.367	∞	14.254	8.230	∞	∞	11.440	15.240	101.239	∞	20.459	∞	4.670	152.445	41.773	∞	∞		0.00046	0.00058	0.00057	0.00056	0.00063	0.00056	0.00049	0.00051	0.00060	0.00057	0.00057	0.00069	0.00054	0.00049	0.00051	0.00047	0.00072	0.00043	0.00052	0.00054	0.00047	0.00059	0.00066	0.00048	0.00075	0.00050	0.00055
**34**	16.995	56.494	∞	27.651	∞	18.857	∞	28.088	55.610	40.884	77.206	∞	∞	16.481	1215.924	28.528	22.570	∞	∞	28.918	∞	∞	∞	∞	∞	∞	∞	80.973	4.445	∞	17.279	∞	31.302		0.00046	0.00045	0.00044	0.00052	0.00043	0.00039	0.00040	0.00051	0.00045	0.00043	0.00057	0.00043	0.00039	0.00040	0.00036	0.00061	0.00031	0.00042	0.00043	0.00036	0.00048	0.00052	0.00037	0.00064	0.00039	0.00045
**35**	1.650	2.129	3.208	2.443	4.206	2.178	3.652	12.274	3.055	2.267	2.674	3.621	5.854	45.283	3.543	7.409	1.896	10.794	69.470	2.115	4.924	23.999	11.908	3.322	3.218	9.668	3.154	290.056	1.042	8.504	1.694	3.234	2.310	7.610		0.00056	0.00056	0.00063	0.00054	0.00051	0.00051	0.00062	0.00056	0.00055	0.00068	0.00055	0.00051	0.00052	0.00048	0.00072	0.00043	0.00054	0.00055	0.00048	0.00059	0.00063	0.00049	0.00076	0.00051	0.00056
**36**	4.686	7.463	34.263	10.694	21.012	8.137	∞	11.297	13.674	7.559	24.293	∞	∞	∞	324.890	∞	4.412	∞	∞	10.915	∞	∞	∞	10.893	34.565	∞	34.435	∞	2.016	∞	6.600	47.732	8.953	∞	21.136		0.00055	0.00063	0.00053	0.00050	0.00051	0.00063	0.00054	0.00053	0.00066	0.00054	0.00050	0.00051	0.00047	0.00071	0.00042	0.00053	0.00054	0.00047	0.00058	0.00061	0.00048	0.00075	0.00050	0.00056
**37**	6.650	11.873	∞	16.147	56.643	12.294	∞	28.571	23.618	11.587	92.614	∞	∞	∞	∞	∞	6.019	∞	∞	19.755	∞	∞	∞	19.087	∞	∞	∞	∞	2.492	∞	10.603	128.704	14.630	∞	10.105	∞		0.00062	0.00054	0.00048	0.00050	0.00060	0.00055	0.00054	0.00067	0.00053	0.00048	0.00050	0.00046	0.00071	0.00042	0.00051	0.00053	0.00046	0.00058	0.00063	0.00047	0.00074	0.00049	0.00054
**38**	∞	∞	∞	∞	∞	∞	∞	4.000	∞	∞	∞	∞	∞	3.936	∞	8.764	∞	13.197	7.202	∞	∞	9.382	14.217	91.251	∞	16.893	∞	4.280	63.136	39.296	∞	∞	∞	29.251	2.091	7.773	12.243		0.00062	0.00055	0.00057	0.00066	0.00063	0.00063	0.00075	0.00060	0.00055	0.00057	0.00053	0.00078	0.00049	0.00058	0.00060	0.00053	0.00065	0.00071	0.00054	0.00080	0.00056	0.00061
**39**	46.687	∞	∞	∞	59.244	∞	∞	4.587	∞	765.952	∞	∞	∞	10.211	∞	∞	13.178	∞	9.961	∞	∞	55.283	∞	12.673	∞	∞	∞	10.420	5.456	∞	∞	∞	∞	55.224	3.016	46.766	756.387	∞		0.00050	0.00049	0.00062	0.00052	0.00051	0.00065	0.00053	0.00050	0.00051	0.00045	0.00069	0.00040	0.00053	0.00053	0.00045	0.00057	0.00058	0.00048	0.00073	0.00048	0.00055
**40**	∞	∞	∞	∞	∞	∞	∞	3.694	∞	∞	∞	∞	∞	4.765	∞	15.604	111.470	23.045	7.252	∞	∞	12.693	24.934	25.028	∞	42.737	∞	5.009	13.170	586.816	∞	∞	∞	28.795	2.113	10.893	19.661	∞	∞		0.00043	0.00051	0.00050	0.00050	0.00062	0.00046	0.00041	0.00043	0.00040	0.00066	0.00036	0.00044	0.00046	0.00040	0.00051	0.00060	0.00040	0.00067	0.00042	0.00047
**41**	∞	∞	∞	∞	∞	∞	∞	8.216	∞	∞	∞	∞	∞	6.263	∞	11.512	∞	36.264	23.102	∞	∞	19.418	38.152	∞	∞	30.858	∞	7.870	29.224	∞	∞	∞	∞	∞	3.487	14.710	29.074	∞	684.696	∞		0.00053	0.00050	0.00051	0.00063	0.00048	0.00043	0.00045	0.00041	0.00066	0.00037	0.00046	0.00047	0.00041	0.00053	0.00060	0.00042	0.00069	0.00044	0.00048
**42**	∞	∞	∞	∞	47.400	∞	∞	4.085	∞	∞	∞	∞	2973.167	5.918	∞	19.442	24.792	18.494	7.655	∞	126.447	16.364	19.515	13.661	∞	48.515	∞	5.775	11.859	46.343	∞	∞	∞	16.889	2.622	12.997	18.906	∞	∞	∞	∞		0.00062	0.00063	0.00074	0.00057	0.00051	0.00054	0.00051	0.00077	0.00048	0.00054	0.00057	0.00051	0.00063	0.00074	0.00051	0.00077	0.00054	0.00057
**43**	∞	∞	44.907	∞	11.010	∞	369.208	2.232	∞	1241.310	∞	49.227	12.081	2.580	3597.686	5.552	13.693	5.174	3.104	∞	11.607	4.796	5.593	5.339	43.678	7.291	∞	2.441	13.777	8.780	∞	514.566	∞	6.372	1.358	3.981	5.131	∞	∞	∞	29.451	∞		0.00053	0.00066	0.00054	0.00050	0.00051	0.00047	0.00070	0.00042	0.00053	0.00054	0.00047	0.00058	0.00060	0.00048	0.00075	0.00050	0.00056
**44**	9.846	20.977	∞	12.549	∞	12.624	∞	29.745	47.041	19.072	39.467	∞	∞	61.524	234.677	118.785	11.798	∞	∞	17.583	∞	∞	∞	∞	∞	∞	∞	∞	3.299	∞	11.539	158.511	17.050	∞	13.002	∞	∞	15.846	27.027	17.535	∞	13.046	4.661		0.00065	0.00054	0.00050	0.00051	0.00045	0.00070	0.00040	0.00053	0.00054	0.00045	0.00057	0.00059	0.00048	0.00074	0.00048	0.00056
**45**	∞	∞	∞	∞	1487.447	∞	∞	3.051	∞	∞	∞	∞	32.146	2.619	∞	5.113	∞	6.484	4.489	∞	136.493	5.344	7.095	22.931	∞	8.255	∞	2.672	∞	12.935	∞	∞	∞	12.344	1.494	4.485	6.335	∞	1145.071	∞	∞	∞	∞	7.941		0.00066	0.00062	0.00063	0.00059	0.00083	0.00054	0.00065	0.00066	0.00059	0.00070	0.00072	0.00060	0.00087	0.00061	0.00068
**46**	∞	∞	∞	24.501	∞	∞	24.375	4.971	∞	∞	37.451	34.768	22.019	2.781	23.772	4.180	∞	6.412	7.002	114.590	7035.316	5.705	7.352	∞	∞	6.758	1099.828	3.131	∞	11.519	∞	112.339	∞	23.482	1.985	4.606	6.283	∞	12.444	116.688	∞	25.483	14.240	12.290	∞		0.00046	0.00048	0.00044	0.00070	0.00040	0.00049	0.00051	0.00044	0.00056	0.00064	0.00045	0.00072	0.00047	0.00052
**47**	∞	∞	∞	∞	∞	∞	∞	3.791	∞	∞	∞	∞	39.594	2.959	416.625	5.302	∞	7.496	5.843	∞	∞	5.962	8.108	174.952	∞	8.746	∞	3.193	∞	14.918	∞	∞	∞	18.482	1.813	5.127	7.278	∞	51.008	∞	∞	∞	∞	10.740	∞	∞		0.00043	0.00040	0.00066	0.00036	0.00044	0.00046	0.00040	0.00051	0.00060	0.00040	0.00067	0.00042	0.00047
**48**	10.269	21.770	∞	12.916	∞	13.037	∞	30.855	36.724	19.727	40.778	∞	∞	135.636	∞	121.912	12.283	∞	∞	18.266	∞	∞	∞	∞	∞	∞	∞	∞	3.447	∞	11.419	161.375	20.173	∞	13.637	∞	∞	16.424	27.858	18.207	∞	14.631	4.838	∞	8.292	12.777	11.164		0.00041	0.00067	0.00037	0.00046	0.00048	0.00041	0.00053	0.00061	0.00042	0.00069	0.00044	0.00048
**49**	∞	46.740	8.927	8.206	11.083	16.727	5.652	2.199	13.407	35.358	6.541	5.284	4.339	1.372	5.230	1.935	∞	2.286	2.461	8.895	6.393	2.358	2.507	7.779	8.913	2.644	8.312	1.371	∞	3.381	17.784	7.699	27.600	4.366	1.035	1.898	2.330	25.203	4.448	8.919	24.547	8.515	8.128	3.232	73.525	∞	∞	3.380		0.00063	0.00033	0.00042	0.00044	0.00037	0.00049	0.00055	0.00038	0.00065	0.00040	0.00045
**50**	∞	∞	∞	∞	∞	∞	∞	3.748	∞	∞	∞	∞	39.728	2.963	378.401	5.308	∞	7.507	6.164	∞	∞	6.835	8.124	189.293	∞	10.848	∞	3.198	∞	14.951	∞	∞	∞	18.587	1.847	5.373	7.289	∞	50.336	∞	∞	∞	∞	10.790	∞	∞	∞	12.325	∞		0.00058	0.00069	0.00069	0.00063	0.00074	0.00077	0.00064	0.00090	0.00066	0.00071
**51**	∞	∞	∞	∞	∞	∞	∞	3.712	∞	∞	∞	∞	∞	3.634	∞	7.844	∞	11.196	6.451	∞	∞	8.434	12.114	43.836	∞	14.354	∞	3.880	38.703	28.538	∞	∞	∞	22.328	1.948	6.921	10.522	∞	∞	∞	∞	∞	∞	13.236	∞	∞	∞	13.751	19.491	∞		0.00039	0.00040	0.00033	0.00045	0.00049	0.00034	0.00061	0.00036	0.00042
**52**	8.649	15.190	342.695	10.180	∞	9.459	51.574	126.491	17.975	14.479	16.773	110.691	∞	26.945	26.518	22.811	11.896	∞	∞	11.120	∞	∞	∞	∞	407.555	∞	51.134	∞	3.547	∞	8.756	29.330	12.363	∞	17.037	∞	∞	11.574	14.741	11.112	96.036	9.365	4.168	∞	6.826	12.343	9.338	∞	3.483	9.388	10.111		0.00049	0.00042	0.00054	0.00063	0.00043	0.00070	0.00045	0.00050
**53**	7.432	12.143	80.706	15.494	35.156	12.374	∞	10.650	20.795	11.858	43.421	∞	∞	∞	∞	∞	6.764	∞	∞	18.060	∞	∞	∞	17.606	81.772	∞	72.486	∞	3.003	∞	11.572	1314.643	14.323	∞	11.799	∞	∞	12.420	69.015	18.031	23.980	19.390	5.803	∞	7.145	6.995	7.967	∞	2.829	8.953	10.973	∞		0.00044	0.00056	0.00063	0.00045	0.00072	0.00047	0.00052
**54**	∞	∞	173.761	∞	17.683	∞	112.384	2.349	∞	∞	∞	34.708	11.484	2.195	47.829	4.068	765.880	4.456	3.229	∞	15.146	4.146	4.844	8.237	159.942	5.730	∞	2.160	∞	7.350	∞	1166.508	∞	6.658	1.293	3.395	4.444	∞	41.859	∞	2632.561	∞	∞	4.924	∞	861.565	∞	5.126	44.557	∞	∞	4.478	5.125		0.00049	0.00055	0.00038	0.00065	0.00040	0.00045
**55**	25.076	6490.792	∞	21.191	∞	25.841	∞	219.018	225.837	74.980	291.065	∞	∞	33.993	∞	44.473	34.821	∞	∞	48.118	∞	∞	∞	∞	∞	∞	∞	746.591	5.695	∞	23.380	∞	89.672	∞	10.075	∞	∞	47.174	48.157	47.878	∞	26.597	7.321	∞	17.414	47.018	26.767	∞	5.622	26.948	33.993	∞	∞	8.612		0.00066	0.00050	0.00077	0.00052	0.00057
**56**	∞	∞	∞	∞	∞	∞	∞	3.946	∞	∞	∞	∞	∞	3.764	∞	8.403	∞	12.494	6.847	∞	∞	8.719	13.516	78.946	∞	16.156	∞	4.054	60.883	36.999	∞	∞	∞	27.423	1.991	7.399	11.629	∞	∞	∞	∞	∞	∞	14.959	∞	∞	∞	15.540	24.082	∞	∞	11.008	12.981	∞	44.297		0.00058	0.00084	0.00058	0.00066
**57**	10.289	23.637	∞	17.855	∞	16.440	∞	14.582	42.548	21.032	318.465	∞	∞	97.098	∞	∞	10.222	∞	∞	29.199	∞	∞	∞	∞	∞	∞	∞	∞	3.197	∞	15.700	∞	21.446	∞	8.996	∞	∞	19.907	107.141	29.038	∞	18.636	5.543	∞	8.889	10.666	13.937	∞	3.039	11.303	16.073	∞	∞	5.444	∞	18.753		0.00066	0.00041	0.00046
**58**	27.579	597.386	∞	∞	∞	∞	∞	6.242	∞	115.448	∞	∞	∞	31.639	∞	∞	12.252	∞	30.185	∞	∞	∞	∞	22.784	∞	∞	∞	36.679	4.799	∞	∞	∞	∞	∞	4.688	∞	∞	∞	∞	∞	∞	∞	32.637	∞	41.080	12.690	29.761	∞	4.204	50.472	∞	37.544	∞	19.093	∞	∞	∞		0.00068	0.00073
**59**	∞	∞	∞	∞	∞	∞	∞	6.702	∞	∞	∞	∞	∞	4.513	∞	8.833	∞	26.000	17.105	∞	∞	12.913	28.329	∞	∞	23.823	∞	5.579	22.148	∞	∞	∞	∞	∞	2.554	10.864	21.271	∞	579.310	∞	∞	∞	22.948	∞	∞	∞	∞	∞	18.350	∞	∞	72.438	19.032	6363.914	∞	∞	2965.539	∞		0.00048
**60**	5.563	9.465	91.104	8.412	∞	7.733	60.125	34.538	14.485	9.426	16.510	∞	∞	∞	37.865	∞	6.357	∞	∞	9.359	∞	∞	∞	185.627	95.524	∞	33.586	∞	2.296	∞	6.148	25.980	9.120	∞	25.512	∞	∞	8.400	16.136	9.335	30.551	8.764	3.396	∞	4.768	6.658	6.132	∞	2.239	6.151	7.362	∞	∞	3.361	∞	7.939	∞	179.830	21.576	
