# Supplementary material for: Migration sources and pathways of the pest species Sogatella furcifera in Yunnan, China, and across the border inferred from DNA and wind analyses
Source: Ecol Evol. 2020 Jul 17;10(15):8235–50. doi: 10.1002/ece3.6531 (PMC7417236; doi:10.1002/ece3.6531)
Supplement: Supplementary file 15 — Table S3 [file ECE3-10-8235-s015.pdf]

TABLE S3 The pairwise *F* st values (below diagonal), Nei's average number of differences within populations (diagonal elements), and Nei's average number of differences between populations (above diagonal), * denotes significance at 0.05 level, ** denotes significa

Pop.	1	2	3	4	5	6	7	8	9	10	11	12	13	14	15	16	17	18	19	20	21	22	23	24	25	26	27	28	29	30	31	32	33	34	35	36	37	38	39	40	41	42	43	44	45	46	47	48	49	50	51	52	53	54	55	56	57	58	59	60
**1**	**1.0911**	1.1173	1.1072	1.5014	1.2187	1.3610	1.3292	1.4401	1.5122	1.2185	1.1980	1.1476	1.1980	1.3488	1.3193	1.3497	1.1072	1.1372	1.2279	1.1275	1.1577	1.4610	1.2383	0.9454	1.1071	1.3597	1.2283	1.1067	1.0670	1.2686	1.0667	1.2288	1.2890	1.2079	1.3389	1.2077	1.1874	1.1887	1.3393	1.1274	1.3705	1.5518	1.2283	1.1779	1.0162	1.1580	1.1479	1.2283	1.0468	1.1483	1.1376	1.3089	1.4912	1.1176	1.4601	1.1378	1.1475	1.3902	1.0160	1.0967
**2**	-0.0384	**1.2294**	1.1629	1.5622	1.2745	1.4267	1.3696	1.4758	1.5730	1.2843	1.2536	1.1982	1.2436	1.3693	1.3649	1.3800	1.1780	1.1728	1.2635	1.1881	1.2083	1.4866	1.2738	1.0010	1.1627	1.3952	1.2840	1.1322	1.1528	1.3092	1.1223	1.2844	1.3548	1.2535	1.3543	1.2381	1.2230	1.2544	1.3899	1.1881	1.4312	1.6126	1.2991	1.2185	1.0868	1.2237	1.2186	1.2689	1.1328	1.2190	1.2033	1.3495	1.5268	1.1934	1.5008	1.2035	1.1881	1.4358	1.0767	1.1323
**3**	-0.0216	-0.0322	**1.1713**	1.5320	1.2344	1.4063	1.3269	1.3956	1.5427	1.2641	1.2134	1.1480	1.1834	1.2588	1.3196	1.2896	1.1679	1.0925	1.1834	1.1579	1.1530	1.4013	1.1934	0.9610	1.1226	1.3149	1.2438	1.0320	1.1729	1.2389	1.1097	1.2441	1.3346	1.1933	1.2341	1.1478	1.1427	1.2342	1.3395	1.1578	1.4009	1.5823	1.2889	1.1484	1.0767	1.2188	1.2084	1.1988	1.1528	1.2088	1.1831	1.2793	1.4462	1.1933	1.4457	1.1834	1.1180	1.3753	1.0467	1.0521
**4**	-0.0069	-0.0120	-0.0130	**1.9325**	1.6639	1.7760	1.7114	1.8914	1.9328	1.6635	1.5876	1.5472	1.6030	1.7039	1.7042	1.6943	1.5925	1.5221	1.6585	1.5320	1.5878	1.8391	1.6232	1.4153	1.5320	1.7298	1.6280	1.4867	1.5671	1.6533	1.4785	1.6387	1.7192	1.6229	1.7447	1.5826	1.5723	1.6186	1.6939	1.5319	1.8209	1.9395	1.6126	1.6031	1.4556	1.6434	1.6027	1.6536	1.5620	1.6033	1.5570	1.7395	1.8766	1.5521	1.8960	1.5674	1.5575	1.7652	1.4658	1.5118
**5**	-0.0092	-0.0194	-0.0289	0.0080	**1.3689**	1.5382	1.4587	1.4490	1.6593	1.3758	1.3452	1.2698	1.2950	1.3654	1.4464	1.4216	1.2541	1.2040	1.2646	1.2897	1.2595	1.5078	1.3050	1.0422	1.2341	1.4367	1.3605	1.1334	1.2744	1.3455	1.2415	1.3658	1.4512	1.2744	1.3051	1.2594	1.2543	1.3558	1.4764	1.2897	1.4923	1.7194	1.4359	1.2398	1.1984	1.3101	1.3201	1.2851	1.2442	1.3203	1.3048	1.3554	1.5580	1.3251	1.5322	1.3052	1.2195	1.5021	1.1481	1.1434
**6**	-0.0254	-0.0267	-0.0209	-0.0227	0.0024	**1.7003**	1.5984	1.7647	1.8071	1.5280	1.4772	1.4315	1.4872	1.6126	1.5935	1.6036	1.4419	1.4161	1.5372	1.4117	1.4620	1.7353	1.5172	1.2745	1.4013	1.6288	1.5175	1.3854	1.4069	1.5526	1.3507	1.5181	1.5888	1.5171	1.6379	1.4816	1.4663	1.4882	1.6035	1.4115	1.6853	1.8267	1.5026	1.4820	1.3204	1.4928	1.4624	1.5325	1.3967	1.4629	1.4369	1.6235	1.7706	1.4170	1.7697	1.4370	1.4415	1.6595	1.3302	1.3956
**7**	-0.0008	-0.0218	-0.0328	-0.0231	-0.0072	-0.0229	**1.5696**	1.6323	1.7372	1.4709	1.3925	1.3395	1.3749	1.4276	1.5064	1.4536	1.3998	1.2687	1.3923	1.3445	1.3698	1.5803	1.3723	1.2001	1.3267	1.4915	1.4355	1.2105	1.4177	1.4328	1.3115	1.4359	1.5367	1.4048	1.4403	1.3267	1.3240	1.4386	1.5087	1.3445	1.6255	1.7568	1.4758	1.3522	1.2911	1.4584	1.4228	1.4052	1.4051	1.4283	1.3849	1.4909	1.6253	1.3952	1.6600	1.3851	1.3118	1.5445	1.2684	1.2559
**8**	0.1261	0.1004	0.0696	0.1122	0.0357	0.1143	0.0825	**1.4258**	1.8608	1.5747	1.5366	1.4260	1.4155	1.3698	1.5974	1.5095	1.4402	1.2816	1.2992	1.5012	1.3823	1.5729	1.3851	1.1549	1.3898	1.5322	1.5320	1.1530	1.5414	1.4459	1.4707	1.5419	1.6551	1.3646	1.2436	1.3143	1.2940	1.5721	1.6552	1.5012	1.6658	1.9416	1.7159	1.3100	1.4273	1.4937	1.5391	1.3603	1.4961	1.5416	1.5215	1.4103	1.6380	1.5974	1.6025	1.5170	1.3050	1.6555	1.3064	1.1906
**9**	-0.0160	-0.0208	-0.0220	-0.0126	-0.0097	-0.0188	-0.0222	0.0844	**1.9818**	1.6743	1.6236	1.5680	1.6135	1.7089	1.7299	1.7249	1.5830	1.5324	1.6281	1.5630	1.5881	1.8469	1.6335	1.3954	1.5426	1.7503	1.6589	1.4866	1.5680	1.6739	1.5045	1.6595	1.7350	1.6333	1.7140	1.5928	1.5826	1.6395	1.7501	1.5629	1.8164	1.9784	1.6743	1.5830	1.4766	1.6339	1.6136	1.6387	1.5528	1.6141	1.5882	1.7295	1.8869	1.5834	1.8811	1.5885	1.5578	1.7908	1.4663	1.5018
**10**	-0.0310	-0.0320	-0.0255	-0.0080	-0.0141	-0.0215	-0.0167	0.0960	-0.0163	**1.4213**	1.3549	1.2994	1.3449	1.4707	1.4712	1.4813	1.2792	1.2740	1.3623	1.2893	1.3096	1.5930	1.3751	1.1022	1.2639	1.4966	1.3852	1.2335	1.2540	1.4105	1.2234	1.3856	1.4561	1.3548	1.4557	1.3394	1.3243	1.3556	1.4912	1.2893	1.5325	1.7139	1.4003	1.3198	1.1879	1.3300	1.3198	1.3702	1.2339	1.3202	1.3045	1.4509	1.6282	1.2945	1.6073	1.3047	1.2894	1.5371	1.1779	1.2335
**11**	-0.0128	-0.0231	-0.0331	-0.0293	-0.0053	-0.0276	-0.0432	0.1014	-0.0217	-0.0175	**1.3358**	1.2286	1.2741	1.3495	1.3904	1.3602	1.2788	1.1832	1.3042	1.2285	1.2589	1.4921	1.2842	1.0817	1.2133	1.3957	1.3244	1.1326	1.2738	1.3295	1.1803	1.3249	1.4155	1.3041	1.3649	1.2386	1.2334	1.3150	1.4003	1.2285	1.5019	1.6432	1.3395	1.2591	1.1574	1.3297	1.2992	1.3095	1.2637	1.2997	1.2536	1.4002	1.5371	1.2640	1.5566	1.2640	1.2186	1.4462	1.1474	1.1628
**12**	-0.0043	-0.0196	-0.0388	-0.0168	-0.0170	-0.0178	-0.0390	0.0745	-0.0190	-0.0140	-0.0376	**1.2138**	1.1936	1.2489	1.3299	1.2795	1.2183	1.0977	1.2037	1.1780	1.1834	1.4015	1.1960	1.0013	1.1479	1.3151	1.2640	1.0322	1.2334	1.2490	1.1375	1.2643	1.3649	1.2186	1.2393	1.1480	1.1479	1.2645	1.3447	1.1781	1.4362	1.5976	1.3091	1.1686	1.1120	1.2692	1.2487	1.2190	1.2183	1.2491	1.2133	1.3046	1.4437	1.2286	1.4685	1.2136	1.1332	1.3805	1.0820	1.0674
**13**	0.0136	-0.0058	-0.0324	0.0004	-0.0198	0.0006	-0.0335	0.0470	-0.0084	-0.0014	-0.0235	-0.0415	**1.2723**	1.2488	1.3703	1.2998	1.2586	1.1077	1.2035	1.2286	1.2085	1.4115	1.2111	1.0213	1.1831	1.3353	1.3045	1.0320	1.2940	1.2691	1.1881	1.3047	1.4155	1.2335	1.2137	1.1580	1.1629	1.3149	1.3902	1.2286	1.4715	1.6534	1.3800	1.1786	1.1675	1.3095	1.2992	1.2290	1.2738	1.2995	1.2638	1.3095	1.4587	1.2944	1.4836	1.2643	1.1483	1.4158	1.1172	1.0722
**14**	0.1555	0.1176	0.0633	0.0846	0.0640	0.1048	0.0345	0.0463	0.0728	0.1132	0.0653	0.0388	0.0153	**1.1871**	1.4104	1.2492	1.4094	1.0874	1.1782	1.3290	1.2585	1.3585	1.1881	1.0966	1.2584	1.3100	1.3800	0.9564	1.5204	1.2688	1.3164	1.3799	1.5337	1.2584	1.0874	1.1074	1.1275	1.4404	1.4404	1.3291	1.5667	1.7466	1.5311	1.1784	1.3184	1.4603	1.4500	1.2238	1.5004	1.4501	1.3895	1.3092	1.4407	1.4706	1.5013	1.3901	1.1482	1.4357	1.2179	1.0468
**15**	0.0015	-0.0159	-0.0287	-0.0199	-0.0068	-0.0179	-0.0333	0.0705	-0.0190	-0.0076	-0.0355	-0.0368	-0.0275	0.0320	**1.5436**	1.4412	1.4000	1.2693	1.3902	1.3398	1.3601	1.5632	1.3702	1.1878	1.3195	1.4818	1.4307	1.2086	1.4102	1.4206	1.3043	1.4311	1.5243	1.4002	1.4308	1.3195	1.3093	1.4313	1.5016	1.3398	1.6080	1.7470	1.4611	1.3501	1.2787	1.4459	1.4204	1.3955	1.4000	1.4209	1.3800	1.4912	1.6231	1.3904	1.6377	1.3803	1.3097	1.5373	1.2586	1.2488
**16**	0.0928	0.0626	0.0194	0.0290	0.0409	0.0464	-0.0070	0.0779	0.0319	0.0619	0.0098	-0.0050	-0.0118	-0.0187	-0.0067	**1.3579**	1.4305	1.1584	1.2796	1.3296	1.3097	1.4421	1.2593	1.1578	1.2893	1.3711	1.4006	1.0575	1.5013	1.3299	1.3118	1.4009	1.5421	1.3297	1.2393	1.1887	1.2086	1.4412	1.4510	1.3296	1.6030	1.7396	1.4912	1.2596	1.3091	1.4815	1.4509	1.3100	1.4912	1.4511	1.3902	1.4008	1.5121	1.4409	1.5826	1.3906	1.2192	1.4717	1.2487	1.1381
**17**	-0.0287	-0.0256	-0.0096	0.0206	-0.0189	-0.0012	0.0154	0.0929	-0.0009	-0.0194	0.0137	0.0147	0.0231	0.1578	0.0249	0.1105	**1.1869**	1.1977	1.2582	1.2083	1.2082	1.5216	1.2988	0.9757	1.1677	1.4304	1.2991	1.1572	1.1176	1.3293	1.1374	1.2995	1.3598	1.2483	1.3590	1.2682	1.2480	1.2593	1.4251	1.2082	1.4211	1.6429	1.3293	1.2184	1.0869	1.1985	1.2086	1.2688	1.0874	1.2089	1.2084	1.3393	1.5519	1.1984	1.5007	1.2086	1.1981	1.4710	1.0665	1.1371
**18**	0.0683	0.0375	-0.0065	0.0275	0.0046	0.0367	-0.0237	0.0427	0.0179	0.0387	0.0011	-0.0212	-0.0383	-0.0185	-0.0131	-0.0299	0.0754	**1.0281**	1.0723	1.1477	1.1074	1.2702	1.0824	0.9304	1.0922	1.2041	1.2136	0.8809	1.2633	1.1479	1.1122	1.2137	1.3446	1.1224	1.0422	1.0168	1.0318	1.2439	1.2892	1.1478	1.3903	1.5724	1.3193	1.0575	1.1068	1.2486	1.2383	1.1079	1.2432	1.2386	1.1930	1.1882	1.3349	1.2437	1.3751	1.1935	1.0273	1.3047	1.0364	0.9411
**19**	0.0853	0.0564	0.0170	0.0692	0.0020	0.0712	0.0215	0.0067	0.0366	0.0544	0.0450	0.0159	-0.0085	0.0060	0.0294	0.0181	0.0693	-0.0180	**1.1552**	1.2687	1.1829	1.3558	1.1731	0.9758	1.1828	1.3049	1.3196	0.9564	1.3390	1.2388	1.2358	1.3094	1.4453	1.1828	1.0672	1.1024	1.1225	1.3497	1.4253	1.2688	1.4660	1.6987	1.4707	1.1130	1.2126	1.3091	1.3292	1.1684	1.3038	1.3242	1.2990	1.2336	1.4207	1.3648	1.4357	1.2995	1.1030	1.4206	1.1120	1.0015
**20**	-0.0243	-0.0303	-0.0321	-0.0285	-0.0033	-0.0339	-0.0370	0.1192	-0.0239	-0.0239	-0.0397	-0.0325	-0.0138	0.0948	-0.0310	0.0310	0.0045	0.0211	0.0644	**1.2188**	1.2084	1.4615	1.2486	1.0263	1.1578	1.3601	1.2689	1.1071	1.1883	1.2890	1.1122	1.2694	1.3499	1.2586	1.3495	1.2080	1.1978	1.2495	1.3497	1.1679	1.4413	1.5824	1.2689	1.2185	1.0869	1.2592	1.2287	1.2690	1.1782	1.2292	1.1982	1.3598	1.5015	1.1884	1.5110	1.1984	1.1781	1.4006	1.0869	1.1273
**21**	-0.0022	-0.0175	-0.0411	0.0043	-0.0315	-0.0020	-0.0217	0.0396	-0.0110	-0.0121	-0.0188	-0.0323	-0.0351	0.0399	-0.0195	0.0122	0.0000	-0.0193	-0.0079	-0.0130	**1.2294**	1.4111	1.2083	0.9809	1.1578	1.3350	1.2843	1.0317	1.2336	1.2538	1.1679	1.2845	1.3852	1.2081	1.2081	1.1577	1.1576	1.2846	1.3851	1.2084	1.4361	1.6382	1.3599	1.1582	1.1323	1.2591	1.2590	1.2086	1.2084	1.2592	1.2337	1.2839	1.4611	1.2592	1.4607	1.2341	1.1330	1.4106	1.0819	1.0568
**22**	0.0795	0.0488	0.0116	0.0400	0.0159	0.0495	-0.0024	0.0386	0.0307	0.0522	0.0167	-0.0034	-0.0170	-0.0253	-0.0051	-0.0251	0.0847	-0.0340	-0.0156	0.0361	-0.0021	**1.5986**	1.3710	1.2413	1.4009	1.4828	1.5225	1.1592	1.6049	1.4416	1.4310	1.5099	1.6535	1.4210	1.3080	1.2952	1.3229	1.5629	1.5932	1.4642	1.6993	1.8718	1.6435	1.3511	1.4356	1.5701	1.5675	1.3965	1.5822	1.5524	1.5119	1.4820	1.6137	1.5728	1.6640	1.5150	1.3209	1.5884	1.3526	1.2320
**23**	0.0629	0.0348	-0.0059	0.0260	0.0044	0.0345	-0.0199	0.0415	0.0170	0.0361	0.0012	-0.0215	-0.0329	-0.0170	-0.0120	-0.0274	0.0698	-0.0429	-0.0164	0.0196	-0.0175	-0.0314	**1.2296**	1.0313	1.1931	1.3050	1.3145	0.9815	1.3645	1.2488	1.2157	1.3147	1.4457	1.2233	1.1429	1.1175	1.1326	1.3450	1.3901	1.2487	1.4914	1.6736	1.4204	1.1583	1.2078	1.3447	1.3395	1.2087	1.3444	1.3397	1.2940	1.2892	1.4207	1.3448	1.4710	1.2945	1.1281	1.4057	1.1374	1.0418
**24**	0.0032	-0.0105	-0.0223	0.0369	-0.0375	0.0216	0.0154	0.0392	0.0055	-0.0048	0.0157	-0.0024	-0.0114	0.0970	0.0162	0.0709	-0.0149	0.0211	0.0014	0.0196	-0.0312	0.0365	0.0191	**0.7936**	0.9606	1.1679	1.0971	0.8548	0.9960	1.0769	0.9832	1.0972	1.1876	1.0009	1.0111	0.9857	0.9807	1.0872	1.2228	1.0263	1.2236	1.4655	1.1875	0.9561	0.9301	1.0266	1.0466	1.0065	0.9609	1.0468	1.0364	1.0717	1.2839	1.0617	1.2532	1.0368	0.9410	1.2434	0.8697	0.8598
**25**	-0.0215	-0.0321	-0.0432	-0.0128	-0.0290	-0.0245	-0.0328	0.0659	-0.0219	-0.0254	-0.0329	-0.0387	-0.0325	0.0631	-0.0286	0.0194	-0.0096	-0.0067	0.0168	-0.0320	-0.0366	0.0115	-0.0060	-0.0225	**1.1708**	1.3147	1.2436	1.0315	1.1728	1.2385	1.1097	1.2440	1.3345	1.1929	1.2333	1.1475	1.1423	1.2341	1.3395	1.1578	1.3981	1.5824	1.2890	1.1479	1.0767	1.2185	1.2083	1.1983	1.1527	1.2087	1.1830	1.2788	1.4459	1.1933	1.4453	1.1833	1.1176	1.3752	1.0464	1.0516
**26**	0.0582	0.0326	-0.0043	0.0165	0.0120	0.0268	-0.0190	0.0550	0.0139	0.0340	-0.0052	-0.0204	-0.0268	-0.0141	-0.0169	-0.0313	0.0713	-0.0373	-0.0059	0.0115	-0.0110	-0.0347	-0.0343	0.0309	-0.0044	**1.4700**	1.4311	1.1083	1.4909	1.3705	1.3347	1.4160	1.5571	1.3550	1.2800	1.2342	1.2543	1.4615	1.4967	1.3601	1.6182	1.7700	1.5217	1.2901	1.3241	1.4813	1.4609	1.3354	1.4758	1.4459	1.4104	1.4261	1.5476	1.4561	1.6079	1.4109	1.2548	1.5072	1.2638	1.1736
**27**	-0.0182	-0.0279	-0.0378	-0.0266	-0.0213	-0.0248	-0.0379	0.0744	-0.0223	-0.0220	-0.0367	-0.0380	-0.0282	0.0590	-0.0323	0.0119	0.0005	-0.0046	0.0280	-0.0360	-0.0277	0.0119	-0.0041	-0.0044	-0.0377	-0.0063	**1.4102**	1.1580	1.2991	1.3548	1.2181	1.3602	1.4508	1.3143	1.3753	1.2690	1.2638	1.3503	1.4405	1.2688	1.5272	1.6885	1.3848	1.2795	1.1927	1.3500	1.3295	1.3299	1.2840	1.3299	1.2991	1.4106	1.5675	1.3043	1.5770	1.2994	1.2440	1.4865	1.1727	1.1832
**28**	0.1480	0.1061	0.0474	0.0827	0.0455	0.0995	0.0234	0.0370	0.0661	0.1017	0.0594	0.0270	-0.0015	-0.0361	0.0326	-0.0178	0.1437	-0.0346	-0.0194	0.0906	0.0189	-0.0324	-0.0312	0.0709	0.0472	-0.0217	0.0480	**0.7948**	1.2580	1.0572	1.0844	1.1580	1.3090	1.0316	0.8810	0.9059	0.9310	1.2083	1.2335	1.1073	1.3345	1.5369	1.3090	0.9568	1.0813	1.2081	1.2078	1.0071	1.2329	1.2080	1.1575	1.0823	1.2338	1.2334	1.2843	1.1581	0.9316	1.2339	0.9810	0.8303
**29**	-0.0129	0.0026	0.0444	0.0419	0.0429	0.0153	0.0689	0.1903	0.0267	0.0065	0.0555	0.0740	0.0948	0.2576	0.0731	0.1913	-0.0099	0.1695	0.1689	0.0367	0.0679	0.1685	0.1572	0.0643	0.0445	0.1480	0.0452	0.2586	**1.0704**	1.3798	1.1096	1.2997	1.3245	1.2938	1.5055	1.3490	1.3135	1.2294	1.4353	1.1880	1.4164	1.6176	1.2687	1.2789	1.0416	1.1684	1.1684	1.3294	1.0119	1.1688	1.1782	1.4000	1.6178	1.1277	1.5460	1.1783	1.2535	1.4963	1.0615	1.2128
**30**	0.0351	0.0122	-0.0204	0.0052	-0.0130	0.0154	-0.0213	0.0377	0.0027	0.0151	-0.0127	-0.0292	-0.0359	-0.0026	-0.0210	-0.0208	0.0431	-0.0389	-0.0140	0.0008	-0.0315	-0.0251	-0.0357	0.0015	-0.0205	-0.0314	-0.0213	-0.0178	0.1204	**1.3571**	1.2611	1.3601	1.4810	1.2689	1.2288	1.1881	1.1981	1.3804	1.4354	1.2890	1.5320	1.7138	1.4455	1.2189	1.2381	1.3802	1.3698	1.2693	1.3598	1.3701	1.3294	1.3448	1.5015	1.3700	1.5317	1.3298	1.1886	1.4611	1.1778	1.1075
**31**	-0.0327	-0.0432	-0.0288	-0.0296	0.0008	-0.0411	-0.0224	0.1372	-0.0282	-0.0354	-0.0370	-0.0224	-0.0035	0.1267	-0.0181	0.0586	-0.0107	0.0379	0.0827	-0.0479	-0.0025	0.0529	0.0369	0.0309	-0.0286	0.0327	-0.0353	0.1207	0.0165	0.0210	**1.1121**	1.2186	1.2890	1.2181	1.3444	1.1852	1.1675	1.1912	1.3065	1.1122	1.3931	1.5238	1.2130	1.1805	1.0286	1.1958	1.1602	1.2335	1.0920	1.1658	1.1374	1.3191	1.4686	1.1173	1.4729	1.1376	1.1401	1.3574	1.0362	1.0995
**32**	-0.0137	-0.0237	-0.0335	-0.0169	-0.0137	-0.0212	-0.0342	0.0836	-0.0190	-0.0181	-0.0325	-0.0338	-0.0242	0.0625	-0.0286	0.0156	0.0046	-0.0004	0.0242	-0.0317	-0.0237	0.0070	-0.0002	0.0003	-0.0334	-0.0135	-0.0331	0.0522	0.0495	-0.0137	-0.0308	**1.4003**	1.4411	1.3246	1.3649	1.2589	1.2639	1.3508	1.4461	1.2693	1.5276	1.6689	1.3905	1.2796	1.1932	1.3504	1.3301	1.3300	1.2845	1.3152	1.2996	1.4157	1.5524	1.3049	1.5771	1.2999	1.2442	1.4716	1.1730	1.1832
**33**	-0.0322	-0.0331	-0.0270	-0.0186	-0.0126	-0.0292	-0.0216	0.0950	-0.0236	-0.0272	-0.0264	-0.0198	-0.0040	0.1012	-0.0214	0.0507	-0.0137	0.0339	0.0573	-0.0330	-0.0105	0.0419	0.0318	0.0049	-0.0269	0.0239	-0.0271	0.0967	0.0033	0.0118	-0.0404	-0.0305	**1.5700**	1.4354	1.5413	1.4050	1.3898	1.4213	1.5469	1.3498	1.6031	1.7495	1.4509	1.4003	1.2535	1.4106	1.3904	1.4457	1.3144	1.3857	1.3700	1.5365	1.6938	1.3551	1.6803	1.3702	1.3649	1.5877	1.2534	1.3140
**34**	0.0286	0.0088	-0.0169	0.0178	-0.0297	0.0258	-0.0055	0.0175	0.0089	0.0121	0.0064	-0.0133	-0.0247	0.0294	0.0004	0.0172	0.0217	-0.0173	-0.0191	0.0170	-0.0285	-0.0043	-0.0158	-0.0237	-0.0171	-0.0058	-0.0141	0.0061	0.1011	-0.0296	0.0281	-0.0026	0.0157	**1.2556**	1.1828	1.1676	1.1726	1.3347	1.4202	1.2587	1.4712	1.6935	1.4202	1.1682	1.1875	1.2992	1.3092	1.2186	1.2637	1.3093	1.2839	1.2787	1.4760	1.3245	1.4758	1.2843	1.1480	1.4457	1.1170	1.0617
**35**	0.2325	0.1902	0.1348	0.1699	0.1062	0.1867	0.1204	0.0391	0.1407	0.1807	0.1575	0.1213	0.0787	0.0109	0.1237	0.0632	0.2087	0.0443	0.0072	0.1912	0.0922	0.0204	0.0403	0.1308	0.1345	0.0492	0.1369	0.0017	0.3243	0.0555	0.2279	0.1339	0.1779	0.0617	**0.9641**	1.0520	1.0924	1.4455	1.4708	1.3496	1.5314	1.7849	1.6021	1.0981	1.3287	1.4100	1.4402	1.1484	1.4653	1.4350	1.3949	1.2083	1.3902	1.5063	1.4359	1.3957	1.0881	1.4408	1.1777	0.9612
**36**	0.0964	0.0628	0.0144	0.0447	0.0232	0.0579	-0.0028	0.0424	0.0353	0.0621	0.0202	-0.0040	-0.0206	-0.0287	0.0015	-0.0303	0.1018	-0.0423	-0.0190	0.0438	-0.0024	-0.0385	-0.0384	0.0439	0.0143	-0.0377	0.0143	-0.0411	0.1987	-0.0304	0.0704	0.0104	0.0529	-0.0051	0.0231	**1.0914**	1.0669	1.3093	1.3395	1.2081	1.4507	1.6228	1.3898	1.0977	1.1773	1.3191	1.3089	1.1480	1.3289	1.3039	1.2584	1.2285	1.3650	1.3192	1.4204	1.2589	1.0675	1.3449	1.0968	0.9762
**37**	0.0699	0.0404	-0.0016	0.0300	0.0088	0.0391	-0.0148	0.0172	0.0207	0.0414	0.0054	-0.0156	-0.0276	-0.0221	-0.0163	-0.0241	0.0767	-0.0398	-0.0124	0.0247	-0.0138	-0.0267	-0.0362	0.0255	-0.0017	-0.0316	-0.0001	-0.0271	0.1671	-0.0328	0.0450	0.0039	0.0331	-0.0120	0.0472	-0.0352	**1.1177**	1.2941	1.3393	1.1979	1.4406	1.6175	1.3695	1.1077	1.1570	1.2989	1.2886	1.1581	1.2935	1.2888	1.2432	1.2385	1.3852	1.2939	1.4152	1.2436	1.0774	1.3549	1.0867	0.9913
**38**	-0.0347	-0.0357	-0.0292	-0.0198	-0.0097	-0.0312	-0.0213	0.1111	-0.0219	-0.0292	-0.0284	-0.0213	-0.0044	0.1127	-0.0175	0.0540	-0.0148	0.0365	0.0649	-0.0356	-0.0114	0.0506	0.0340	0.0055	-0.0290	0.0288	-0.0291	0.1046	0.0079	0.0126	-0.0414	-0.0251	-0.0339	0.0168	0.1930	0.0604	0.0392	**1.3690**	1.4463	1.2494	1.5076	1.6640	1.3505	1.2997	1.1532	1.3102	1.2900	1.3501	1.2142	1.2905	1.2696	1.4358	1.5980	1.2548	1.5872	1.2698	1.2643	1.4971	1.1530	1.2134
**39**	0.0106	-0.0032	-0.0192	-0.0307	0.0084	-0.0163	-0.0369	0.0983	-0.0117	0.0007	-0.0337	-0.0311	-0.0184	0.0467	-0.0332	-0.0052	0.0366	-0.0035	0.0478	-0.0292	-0.0067	0.0090	-0.0031	0.0380	-0.0190	-0.0120	-0.0307	0.0458	0.0840	-0.0158	-0.0223	-0.0233	-0.0114	0.0090	0.1422	0.0106	0.0007	-0.0123	**1.5592**	1.3497	1.6382	1.7595	1.4558	1.3801	1.2937	1.4811	1.4405	1.4306	1.4302	1.4410	1.3950	1.5213	1.6431	1.4003	1.6829	1.3952	1.3346	1.5523	1.2836	1.2788
**40**	-0.0244	-0.0303	-0.0321	-0.0286	-0.0032	-0.0340	-0.0370	0.1192	-0.0239	-0.0239	-0.0397	-0.0325	-0.0138	0.0950	-0.0309	0.0311	0.0045	0.0212	0.0645	-0.0436	-0.0130	0.0379	0.0197	0.0196	-0.0319	0.0116	-0.0360	0.0908	0.0366	0.0009	-0.0479	-0.0317	-0.0330	0.0171	0.1913	0.0439	0.0248	-0.0356	-0.0291	**1.2188**	1.4412	1.5823	1.2688	1.2186	1.0868	1.2590	1.2285	1.2690	1.1780	1.2291	1.1981	1.3597	1.5016	1.1883	1.5110	1.1983	1.1781	1.4006	1.0868	1.1274
**41**	-0.0236	-0.0286	-0.0300	-0.0015	-0.0332	-0.0132	-0.0102	0.0574	-0.0175	-0.0232	-0.0156	-0.0196	-0.0150	0.0739	-0.0132	0.0416	-0.0209	0.0136	0.0212	-0.0177	-0.0250	0.0251	0.0129	-0.0249	-0.0320	0.0159	-0.0231	0.0597	0.0168	-0.0025	-0.0146	-0.0196	-0.0245	-0.0095	0.1254	0.0329	0.0169	-0.0227	0.0007	-0.0177	**1.7147**	1.8664	1.5727	1.4312	1.3451	1.4720	1.4720	1.4766	1.3912	1.4722	1.4566	1.5573	1.7447	1.4618	1.7189	1.4568	1.4059	1.6688	1.3146	1.3397
**42**	-0.0069	-0.0119	-0.0128	-0.0225	0.0104	-0.0222	-0.0256	0.1091	-0.0149	-0.0080	-0.0254	-0.0165	0.0002	0.0779	-0.0239	0.0251	0.0198	0.0263	0.0613	-0.0278	0.0039	0.0297	0.0250	0.0353	-0.0127	0.0102	-0.0199	0.0797	0.0405	0.0107	-0.0323	-0.0289	-0.0300	0.0288	0.1601	0.0370	0.0258	-0.0226	-0.0211	-0.0279	-0.0043	**2.0340**	1.6732	1.6534	1.5060	1.6939	1.6532	1.6989	1.6125	1.6436	1.6177	1.8001	1.9168	1.6026	1.9388	1.6179	1.6078	1.8004	1.5161	1.5620
**43**	-0.0051	-0.0036	0.0110	-0.0265	0.0434	-0.0243	0.0014	0.1830	-0.0034	0.0004	-0.0130	0.0101	0.0397	0.1623	0.0001	0.0826	0.0352	0.0881	0.1387	-0.0233	0.0413	0.0944	0.0821	0.0856	0.0113	0.0642	-0.0068	0.1700	0.0350	0.0539	-0.0264	0.0010	-0.0160	0.0728	0.2691	0.1116	0.0888	-0.0170	-0.0088	-0.0234	0.0167	-0.0196	**1.3781**	1.4001	1.1776	1.3801	1.3294	1.4507	1.2686	1.3302	1.2990	1.5465	1.6735	1.2588	1.6826	1.2991	1.3495	1.5320	1.2179	1.3191
**44**	0.0483	0.0233	-0.0110	0.0383	-0.0162	0.0381	-0.0059	0.0165	0.0105	0.0256	0.0125	-0.0118	-0.0280	0.0081	0.0021	0.0042	0.0407	-0.0302	-0.0360	0.0277	-0.0276	-0.0175	-0.0275	-0.0168	-0.0112	-0.0158	-0.0008	-0.0168	0.1316	-0.0287	0.0415	0.0031	0.0285	-0.0300	0.0370	-0.0213	-0.0240	0.0306	0.0182	0.0277	-0.0011	0.0369	0.0969	**1.1508**	1.1575	1.2693	1.2792	1.1587	1.2488	1.2793	1.2488	1.2291	1.4110	1.2994	1.4209	1.2493	1.0882	1.3906	1.0771	0.9969
**45**	-0.0422	-0.0381	-0.0208	-0.0166	0.0003	-0.0327	-0.0056	0.1408	-0.0188	-0.0305	-0.0207	-0.0076	0.0153	0.1603	-0.0052	0.0891	-0.0185	0.0716	0.1002	-0.0331	0.0037	0.0856	0.0658	0.0213	-0.0206	0.0571	-0.0217	0.1576	-0.0068	0.0372	-0.0398	-0.0171	-0.0359	0.0389	0.2507	0.1003	0.0732	-0.0388	0.0004	-0.0332	-0.0192	-0.0163	-0.0212	0.0592	**1.0270**	1.1377	1.1174	1.2079	1.0265	1.1178	1.1020	1.2936	1.4607	1.0769	1.4397	1.0971	1.1221	1.3496	0.9906	1.0764
**46**	-0.0275	-0.0288	-0.0091	0.0200	-0.0142	-0.0011	0.0201	0.0914	-0.0008	-0.0187	0.0132	0.0142	0.0222	0.1524	0.0206	0.1068	-0.0327	0.0723	0.0667	0.0043	0.0001	0.0806	0.0637	-0.0141	-0.0091	0.0689	0.0005	0.1377	-0.0095	0.0416	-0.0038	0.0044	-0.0132	0.0209	0.2012	0.0979	0.0737	-0.0142	0.0386	0.0043	-0.0201	0.0192	0.0339	0.0391	-0.0177	**1.2885**	1.2595	1.3197	1.1382	1.2597	1.2592	1.3902	1.6029	1.2492	1.5466	1.2595	1.2490	1.5218	1.1174	1.1880
**47**	-0.0388	-0.0353	-0.0199	-0.0065	-0.0085	-0.0237	-0.0062	0.1165	-0.0150	-0.0286	-0.0120	-0.0041	0.0125	0.1446	0.0012	0.0862	-0.0262	0.0625	0.0788	-0.0225	-0.0021	0.0774	0.0581	0.0029	-0.0198	0.0541	-0.0169	0.1354	-0.0117	0.0324	-0.0369	-0.0127	-0.0298	0.0263	0.2162	0.0889	0.0643	-0.0321	0.0097	-0.0226	-0.0219	-0.0064	-0.0049	0.0445	-0.0385	-0.0252	**1.2938**	1.3296	1.1483	1.2496	1.2388	1.4104	1.5926	1.2187	1.5615	1.2390	1.2386	1.4914	1.1172	1.1979
**48**	0.0464	0.0225	-0.0105	0.0373	-0.0196	0.0369	-0.0038	0.0160	0.0134	0.0247	0.0121	-0.0112	-0.0268	0.0037	-0.0015	0.0041	0.0391	-0.0288	-0.0299	0.0266	-0.0264	-0.0205	-0.0263	-0.0159	-0.0107	-0.0190	-0.0007	-0.0160	0.1267	-0.0275	0.0420	0.0031	0.0242	-0.0287	0.0354	-0.0204	-0.0229	0.0295	0.0176	0.0267	-0.0044	0.0331	0.0937	-0.0366	0.0569	0.0377	0.0429	**1.2515**	1.2992	1.3246	1.2993	1.2794	1.4614	1.3499	1.4663	1.2998	1.1385	1.4360	1.1275	1.0472
**49**	-0.0045	0.0106	0.0530	0.0574	0.0432	0.0290	0.0813	0.1853	0.0360	0.0139	0.0710	0.0864	0.1033	0.2671	0.0873	0.2053	-0.0111	0.1795	0.1688	0.0532	0.0725	0.1750	0.1663	0.0604	0.0531	0.1590	0.0567	0.2672	-0.0291	0.1288	0.0274	0.0610	0.0178	0.1028	0.3257	0.2085	0.1767	0.0195	0.1011	0.0531	0.0200	0.0555	0.0580	0.1340	0.0068	-0.0106	-0.0040	0.1289	**1.0121**	1.1486	1.1631	1.3698	1.5977	1.1227	1.5159	1.1632	1.2284	1.4836	1.0364	1.1827
**50**	-0.0387	-0.0352	-0.0199	-0.0064	-0.0086	-0.0236	-0.0026	0.1177	-0.0149	-0.0286	-0.0119	-0.0040	0.0124	0.1444	0.0013	0.0861	-0.0263	0.0625	0.0750	-0.0224	-0.0022	0.0682	0.0580	0.0026	-0.0198	0.0441	-0.0168	0.1352	-0.0116	0.0324	-0.0322	-0.0245	-0.0336	0.0262	0.2131	0.0851	0.0642	-0.0320	0.0098	-0.0224	-0.0220	-0.0126	-0.0046	0.0443	-0.0384	-0.0252	-0.0356	0.0390	-0.0041	**1.2945**	1.2393	1.4104	1.5826	1.2194	1.5617	1.2395	1.2490	1.4816	1.1176	1.1980
**51**	-0.0316	-0.0328	-0.0258	-0.0239	-0.0058	-0.0287	-0.0201	0.1187	-0.0193	-0.0262	-0.0337	-0.0178	-0.0002	0.1209	-0.0143	0.0599	-0.0108	0.0428	0.0719	-0.0327	-0.0073	0.0560	0.0396	0.0113	-0.0257	0.0337	-0.0261	0.1142	0.0128	0.0172	-0.0410	-0.0219	-0.0313	0.0219	0.2043	0.0674	0.0454	-0.0338	-0.0090	-0.0327	-0.0198	-0.0169	-0.0139	0.0364	-0.0358	-0.0104	-0.0291	0.0351	0.0250	-0.0290	**1.2559**	1.3850	1.5470	1.2034	1.5362	1.2186	1.2134	1.4459	1.1020	1.1626
**52**	0.0547	0.0319	0.0015	0.0468	-0.0154	0.0502	0.0096	0.0039	0.0271	0.0334	0.0290	0.0045	-0.0141	0.0182	0.0185	0.0215	0.0403	-0.0148	-0.0291	0.0430	-0.0176	-0.0062	-0.0135	-0.0158	0.0012	-0.0005	0.0097	-0.0065	0.1235	-0.0190	0.0540	0.0168	0.0389	-0.0320	0.0285	-0.0074	-0.0099	0.0414	0.0328	0.0431	0.0052	0.0507	0.1071	-0.0311	0.0683	0.0389	0.0508	-0.0298	0.1255	0.0506	0.0471	**1.3836**	1.5420	1.4408	1.5418	1.3855	1.2139	1.5318	1.2029	1.1174
**53**	0.0630	0.0396	0.0062	0.0313	0.0140	0.0388	-0.0069	0.0448	0.0235	0.0405	0.0114	-0.0103	-0.0200	-0.0031	-0.0003	-0.0123	0.0688	-0.0231	-0.0060	0.0269	-0.0036	-0.0231	-0.0322	0.0276	0.0061	-0.0253	0.0069	-0.0124	0.1427	-0.0191	0.0414	0.0004	0.0337	-0.0023	0.0407	-0.0237	-0.0183	0.0387	0.0072	0.0270	0.0204	0.0251	0.0793	-0.0114	0.0654	0.0667	0.0591	-0.0109	0.1502	0.0529	0.0436	-0.0009	**1.7033**	1.5979	1.7139	1.5424	1.3807	1.6485	1.3901	1.2943
**54**	-0.0288	-0.0214	0.0029	-0.0118	0.0275	-0.0264	0.0044	0.1755	-0.0074	-0.0157	-0.0064	0.0142	0.0417	0.1856	0.0104	0.1095	0.0007	0.1009	0.1341	-0.0213	0.0320	0.1076	0.0936	0.0572	0.0031	0.0803	-0.0039	0.1879	-0.0104	0.0637	-0.0384	0.0004	-0.0252	0.0699	0.2789	0.1284	0.1011	-0.0270	0.0118	-0.0213	0.0002	-0.0116	-0.0274	0.0922	-0.0379	0.0006	-0.0265	0.0889	0.0111	-0.0263	-0.0239	0.1005	0.0889	**1.2084**	1.5767	1.2035	1.2589	1.4614	1.1072	1.2234
**55**	0.0196	0.0001	-0.0179	0.0231	-0.0250	0.0190	-0.0065	0.0023	0.0022	0.0066	0.0017	-0.0166	-0.0260	0.0145	-0.0123	0.0111	0.0142	-0.0182	-0.0195	0.0103	-0.0274	-0.0128	-0.0203	-0.0236	-0.0181	-0.0082	-0.0089	0.0007	0.0807	-0.0215	0.0209	-0.0057	0.0055	-0.0258	0.0473	-0.0080	-0.0210	0.0105	0.0103	0.0103	-0.0143	0.0185	0.0639	-0.0285	0.0279	0.0105	0.0183	-0.0310	0.0817	0.0182	0.0145	-0.0234	-0.0139	0.0549	**1.7720**	1.5367	1.4007	1.6935	1.3693	1.3143
**56**	-0.0363	-0.0372	-0.0302	-0.0207	-0.0098	-0.0324	-0.0240	0.1125	-0.0226	-0.0303	-0.0296	-0.0221	-0.0043	0.1173	-0.0181	0.0562	-0.0152	0.0385	0.0681	-0.0372	-0.0115	0.0542	0.0357	0.0063	-0.0301	0.0300	-0.0302	0.1098	0.0082	0.0133	-0.0457	-0.0260	-0.0352	0.0179	0.2007	0.0633	0.0412	-0.0380	-0.0128	-0.0372	-0.0234	-0.0202	-0.0181	0.0323	-0.0455	-0.0145	-0.0334	0.0312	0.0203	-0.0333	-0.0352	0.0435	0.0371	-0.0284	0.0112	**1.2670**	1.2139	1.4462	1.1023	1.1632
**57**	0.0463	0.0207	-0.0147	0.0272	-0.0113	0.0295	-0.0167	0.0332	0.0116	0.0232	0.0016	-0.0199	-0.0319	0.0051	-0.0083	-0.0070	0.0466	-0.0346	-0.0212	0.0168	-0.0270	-0.0206	-0.0315	-0.0049	-0.0149	-0.0232	-0.0079	-0.0157	0.1352	-0.0326	0.0309	-0.0038	0.0228	-0.0249	0.0527	-0.0253	-0.0281	0.0245	0.0047	0.0169	-0.0002	0.0261	0.0827	-0.0331	0.0533	0.0448	0.0346	-0.0316	0.1413	0.0424	0.0302	-0.0220	-0.0143	0.0841	-0.0244	0.0260	**1.0976**	1.3502	1.0519	0.9768
**58**	0.0178	0.0008	-0.0220	-0.0119	-0.0015	-0.0063	-0.0389	0.0742	-0.0112	0.0043	-0.0288	-0.0336	-0.0284	0.0156	-0.0354	-0.0184	0.0392	-0.0224	0.0163	-0.0205	-0.0170	-0.0194	-0.0206	0.0215	-0.0219	-0.0317	-0.0259	0.0135	0.0944	-0.0255	-0.0136	-0.0329	-0.0108	-0.0013	0.0964	-0.0154	-0.0176	-0.0049	-0.0304	-0.0205	-0.0051	-0.0203	0.0151	-0.0034	0.0120	0.0379	0.0165	-0.0067	0.1063	0.0098	-0.0013	0.0131	-0.0140	0.0255	-0.0073	-0.0050	-0.0137	**1.6397**	1.3193	1.2842
**59**	-0.0319	-0.0380	-0.0400	-0.0022	-0.0342	-0.0171	-0.0152	0.0694	-0.0187	-0.0302	-0.0204	-0.0257	-0.0195	0.0998	-0.0128	0.0536	-0.0279	0.0189	0.0284	-0.0234	-0.0330	0.0373	0.0173	-0.0344	-0.0400	0.0206	-0.0301	0.0823	0.0221	-0.0030	-0.0219	-0.0256	-0.0275	-0.0122	0.1637	0.0440	0.0230	-0.0298	0.0009	-0.0234	-0.0347	-0.0025	0.0213	-0.0011	-0.0260	-0.0266	-0.0291	-0.0009	0.0265	-0.0291	-0.0262	0.0069	0.0256	0.0001	-0.0143	-0.0309	0.0002	-0.0026	**1.0057**	0.9859
**60**	0.0825	0.0502	0.0055	0.0561	-0.0016	0.0607	0.0083	0.0143	0.0334	0.0504	0.0294	-0.0003	-0.0230	-0.0071	0.0130	-0.0014	0.0729	-0.0358	-0.0368	0.0507	-0.0177	-0.0227	-0.0323	0.0027	0.0052	-0.0189	0.0147	-0.0335	0.1788	-0.0287	0.0752	0.0189	0.0520	-0.0253	0.0192	-0.0310	-0.0286	0.0562	0.0301	0.0508	0.0161	0.0540	0.1284	-0.0394	0.0949	0.0699	0.0754	-0.0375	0.1825	0.0752	0.0636	-0.0315	-0.0140	0.1295	-0.0247	0.0593	-0.0336	0.0028	0.0227	**0.9215**
