# Supplementary material for: Migration sources and pathways of the pest species Sogatella furcifera in Yunnan, China, and across the border inferred from DNA and wind analyses
Source: Ecol Evol. 2020 Jul 17;10(15):8235–50. doi: 10.1002/ece3.6531 (PMC7417236; doi:10.1002/ece3.6531)
Supplement: Supplementary file 16 — Table S4 [file ECE3-10-8235-s016.pdf]

TABLE S4 Tajima's *D*, Fu's F_s, the sum of squared deviations (SSD), and the raggedness index (RI) of each population and the global dataset with *P* values in parentheses.

Population	Tajima's *D*	Fu's F_s	SSD	RI
All	-2.6343 (0.000)	-26.6597 (0.000)	0.0002 (0.596)	0.0387 (0.585)
1	-0.7357 (0.262)	-4.7018 (0.004)	0.0032 (0.781)	0.0229 (0.916)
2	-0.9653 (0.161)	-2.5466 (0.049)	0.0078 (0.820)	0.0331 (0.938)
3	0.0916 (0.571)	-2.2687 (0.036)	0.0017 (0.857)	0.0613 (0.575)
4	-1.1202 (0.133)	-9.6972 (0.000)	0.0015 (0.868)	0.0436 (0.564)
5	-1.0251 (0.171)	-3.4995 (0.016)	0.0044 (0.608)	0.0609 (0.557)
6	-0.4088 (0.396)	-1.6632 (0.096)	0.0129 (0.264)	0.1268 (0.158)
7	-0.6919 (0.266)	-5.9186 (0.003)	0.0028 (0.705)	0.0402 (0.587)
8	-1.1558 (0.104)	-5.3954 (0.000)	0.0067 (0.422)	0.0690 (0.272)
9	-0.6479 (0.325)	-4.3346 (0.007)	0.0026 (0.713)	0.0482 (0.581)
10	0.5541 (0.716)	-0.8228 (0.276)	0.0323 (0.305)	0.1338 (0.491)
11	-0.9653 (0.194)	-5.7301 (0.000)	0.0070 (0.407)	0.0851 (0.306)
12	0.8367 (0.840)	-4.6124 (0.000)	0.0037 (0.761)	0.0360 (0.921)
13	-1.6917 (0.030)	-6.5686 (0.000)	0.0116 (0.313)	0.1123 (0.221)
14	0.9389 (0.849)	0.1681 (0.514)	0.0294 (0.366)	0.1327 (0.532)
15	-0.3517 (0.407)	-3.1808 (0.015)	0.0065 (0.448)	0.0922 (0.269)
16	0.9389 (0.825)	-3.3189 (0.012)	0.0079 (0.407)	0.1032 (0.241)
17	-0.8585 (0.224)	-3.1859 (0.023)	0.0482 (0.219)	0.1535 (0.178)
18	-0.6479 (0.302)	-6.5804 (0.000)	0.0020 (0.784)	0.0436 (0.724)
19	-0.7094 (0.242)	-8.4495 (0.000)	0.0021 (0.769)	0.0461 (0.522)
20	-0.9396 (0.177)	-3.7076 (0.009)	0.0014 (0.867)	0.0563 (0.567)
21	-0.8463 (0.229)	-2.0452 (0.081)	0.0101 (0.347)	0.0593 (0.690)
22	-0.5488 (0.322)	-9.5026 (0.000)	0.0033 (0.630)	0.0652 (0.395)
23	-0.8452 (0.241)	-6.1463 (0.001)	0.0035 (0.659)	0.0526 (0.518)
24	-1.2914 (0.087)	-2.6113 (0.040)	0.0345 (0.295)	0.1075 (0.378)
25	-0.4088 (0.380)	-3.3273 (0.020)	0.0450 (0.053)	0.0458 (0.820)
26	-0.0570 (0.491)	-6.0059 (0.000)	0.0038 (0.596)	0.0642 (0.413)
27	0.2217 (0.632)	-4.6633 (0.001)	0.0046 (0.551)	0.0780 (0.393)
28	-0.5802 (0.320)	-2.4869 (0.036)	0.1620 (0.015)	0.0475 (0.998)
29	-0.4088 (0.381)	-2.2934 (0.053)	0.0155 (0.370)	0.0758 (0.574)
30	-1.2408 (0.117)	-10.6042 (0.000)	0.0082 (0.341)	0.0688 (0.258)
31	-0.7669 (0.230)	-1.6315 (0.116)	0.0043 (0.606)	0.0541 (0.652)
32	-0.1111 (0.472)	-5.1153 (0.000)	0.0059 (0.521)	0.0560 (0.621)
33	-0.6281 (0.327)	-1.6602 (0.117)	0.0076 (0.434)	0.0800 (0.408)
34	0.7703 (0.802)	-0.4666 (0.356)	0.0021 (0.924)	0.0350 (0.975)
35	-1.1835 (0.105)	-8.6411 (0.000)	0.0024 (0.784)	0.0472 (0.661)
36	-1.1567 (0.151)	-6.0367 (0.001)	0.0422 (0.050)	0.0456 (0.797)
37	-0.7669 (0.229)	-5.2241 (0.002)	0.0029 (0.721)	0.0578 (0.548)
38	-1.0419 (0.153)	-9.2486 (0.000)	0.0180 (0.135)	0.1044 (0.095)
39	-1.0248 (0.175)	-3.7126 (0.008)	0.0110 (0.526)	0.0635 (0.713)

40	0.2132 (0.624)	-3.0237 (0.018)	0.0045 (0.599)	0.0606 (0.541)
41	0.5541 (0.740)	-8.8079 (0.000)	0.0166 (0.136)	0.0844 (0.164)
42	-0.6083 (0.288)	-5.7967 (0.001)	0.0088 (0.304)	0.0663 (0.311)
43	-1.2923 (0.101)	-3.3761 (0.013)	0.0043 (0.733)	0.0390 (0.881)
44	-1.3171 (0.088)	-5.0421 (0.000)	0.0196 (0.151)	0.1093 (0.200)
45	-1.5358 (0.042)	-6.7954 (0.001)	0.0035 (0.796)	0.0261 (0.903)
46	-0.0432 (0.541)	-4.9505 (0.003)	0.0028 (0.758)	0.0651 (0.425)
47	0.2132 (0.643)	-0.9102 (0.270)	0.0025 (0.777)	0.0574 (0.576)
48	0.3078 (0.675)	-1.8505 (0.120)	0.0045 (0.637)	0.0665 (0.508)
49	0.0916 (0.582)	-2.9733 (0.023)	0.0132 (0.224)	0.0955 (0.223)
50	-1.3879 (0.076)	-6.6081 (0.000)	0.0065 (0.532)	0.0408 (0.651)
51	0.4877 (0.717)	-4.0139 (0.004)	0.0113 (0.268)	0.0993 (0.185)
52	-0.4545 (0.373)	-2.1369 (0.070)	0.0039 (0.616)	0.0701 (0.437)
53	-0.3288 (0.404)	-3.4759 (0.015)	0.0066 (0.458)	0.0617 (0.481)
54	-0.1111 (0.475)	-1.7286 (0.130)	0.0032 (0.655)	0.0656 (0.424)
55	-0.5289 (0.335)	-2.9984 (0.023)	0.0044 (0.573)	0.0821 (0.346)
56	-1.2543 (0.098)	-5.9602 (0.000)	0.0046 (0.646)	0.0344 (0.811)
57	0.1997 (0.602)	-2.9237 (0.025)	0.0075 (0.543)	0.0461 (0.788)
58	-1.0614 (0.153)	-5.1535 (0.004)	0.0044 (0.569)	0.0537 (0.458)
59	-0.3174 (0.425)	-1.8195 (0.110)	0.0550 (0.056)	0.0390 (0.966)
60	-0.1803 (0.460)	-3.8107 (0.007)	0.0149 (0.189)	0.0790 (0.270)
