# Supplementary material for: Migration sources and pathways of the pest species Sogatella furcifera in Yunnan, China, and across the border inferred from DNA and wind analyses
Source: Ecol Evol. 2020 Jul 17;10(15):8235–50. doi: 10.1002/ece3.6531 (PMC7417236; doi:10.1002/ece3.6531)
Supplement: Supplementary file 17 — Table S5 [file ECE3-10-8235-s017.pdf]

TABLE S5 Migration rates between the 15 population groups of *S. furcifera* .

Group	TL	LA	MY	VN	C34	D3	D4	D56	E2	E3	E4	E5	F4	F5	G56
TL		0.0	0.0	0.0	0.0	0.0	1199.2	0.0	0.0	0.0	0.0	0.0	3683.3	0.0	0.0
LA	0.0		4348.8	1200.7	0.0	0.0	221.9	0.0	0.0	0.0	0.0	110.9	0.0	3702.9	110.9
MY	0.0	0.0		0.0	0.0	0.0	0.0	0.0	669.1	133.8	0.0	0.0	5353.8	0.0	0.0
VN	0.0	0.0	0.0		0.0	0.0	441.2	0.0	588.6	515.2	0.0	0.0	4427.7	0.0	735.7
C34	0.0	0.0	0.0	0.0		2153.0	20475.3	14893.3	1077.9	0.0	0.0	0.0	37725.4	7542.8	2155.9
D3	9030.3	17139.2	0.0	21447.1	6769.8		57156.8	9027.3	7901.5	0.0	34269.5	2257.5	0.0	0.0	0.0
D4	9849.4	8206.2	10772.2	2955.4	12352.4	17070.8		0.0	0.0	0.0	0.0	0.0	0.0	0.0	0.0
D56	0.0	0.0	0.0	3243.2	0.0	1473.5	0.0		232.6	0.0	1085.6	0.0	2806.5	0.0	465.1
E2	851.3	0.0	1418.8	378.1	0.0	0.0	283.7	0.0		3121.3	1324.0	0.0	283.7	0.0	0.0
E3	0.0	0.0	0.0	0.0	0.0	0.0	0.0	0.0	0.0		1845.6	21394.6	47987.1	7385.0	11073.9
E4	230509.9	9186.6	0.0	162441.4	27559.6	0.0	64318.6	0.0	0.0	0.0		125646.8	73493.1	0.0	0.0
E5	0.0	6155.5	7865.3	4723.8	4102.7	3481.6	5749.8	6155.4	10041.9	8674.9	2051.8		0.0	0.0	0.0
F4	27.9	0.0	167.5	0.0	0.0	0.0	83.7	0.0	0.0	0.0	0.0	0.0		0.0	0.0
F5	0.0	0.0	0.0	0.0	0.0	0.0	0.0	0.0	189.5	2291.6	0.0	0.0	3584.2		378.7
G56	0.0	0.0	0.0	841.8	0.0	0.0	210.4	0.0	0.0	0.0	714.4	280.6	2722.8	1460.3	
